# Gene Expression Profiling Reveals Functional Specialization along the Intestinal Tract of a Carnivorous Teleostean Fish (*Dicentrarchus labrax*)

**DOI:** 10.3389/fphys.2016.00359

**Published:** 2016-08-25

**Authors:** Josep A. Calduch-Giner, Ariadna Sitjà-Bobadilla, Jaume Pérez-Sánchez

**Affiliations:** ^1^Nutrigenomics and Fish Growth Endocrinology Group, Biology, Culture and Pathology of Marine Species, Institute of Aquaculture Torre de la Sal (IATS-CSIC)Castellón, Spain; ^2^Fish Pathology Group, Biology, Culture and Pathology of Marine Species, Institute of Aquaculture Torre de la Sal (IATS-CSIC)Castellón, Spain

**Keywords:** European sea bass, intestine, transcriptome, next-generation sequencing, microarray, immune response

## Abstract

High-quality sequencing reads from the intestine of European sea bass were assembled, annotated by similarity against protein reference databases and combined with nucleotide sequences from public and private databases. After redundancy filtering, 24,906 non-redundant annotated sequences encoding 15,367 different gene descriptions were obtained. These annotated sequences were used to design a custom, high-density oligo-microarray (8 × 15 K) for the transcriptomic profiling of anterior (AI), middle (MI), and posterior (PI) intestinal segments. Similar molecular signatures were found for AI and MI segments, which were combined in a single group (AI-MI) whereas the PI outstood separately, with more than 1900 differentially expressed genes with a fold-change cutoff of 2. Functional analysis revealed that molecular and cellular functions related to feed digestion and nutrient absorption and transport were over-represented in AI-MI segments. By contrast, the initiation and establishment of immune defense mechanisms became especially relevant in PI, although the microarray expression profiling validated by qPCR indicated that these functional changes are gradual from anterior to posterior intestinal segments. This functional divergence occurred in association with spatial transcriptional changes in nutrient transporters and the mucosal chemosensing system via G protein-coupled receptors. These findings contribute to identify key indicators of gut functions and to compare different fish feeding strategies and immune defense mechanisms acquired along the evolution of teleosts.

## Introduction

Teleost fish constitute the most abundant vertebrate group, exhibiting an amazing level of biodiversity affecting their morphology, ecology, and behavior as well as many other aspects of their biology. This huge variability makes fish extremely attractive for the study of many biological questions. They show a high assortment of morphology, anatomy and histology of their gastrointestinal (GI) tract in association with their numerous specialized functions (Wilson and Castro, 2010), as it is involved not only in digestion and feed absorption but also in water and electrolyte balance, nutrient sensing, and immunity (Cain and Swan, 2010). This diversity is now starting to be elucidated and molecular approaches are helping to understand the many vital functions conducted by the GI tract in the context of integrative and comparative physiology. Thus, fish GI tract is an important immunological site acting as a physical and chemical barrier against invading organisms, and the cells within the intestine produce a range of chemical substances to enhance barrier function, innate immunity, and humoral immune responses (Rombout et al., 2011; Xia et al., 2013). This is particularly important at the posterior intestine, where the uptake of macromolecules and foreign antigens mainly occurs (Fuglem et al., 2010; Løkka et al., 2014). Nutrient availability is also essential for the generation and maintenance of a protective effector immune system in humans and other model species (Fox et al., 2005; Michalek and Rathmell, 2010). For instance, a deficiency in glucose uptake negatively affects T cell function with impairment of both proliferation and cytokine expression. Similarly, deficiencies in amino acids, such as tryptophan, arginine, glutamine and cysteine, reduce immune cell activation. Furthermore, short-chain fatty acids provide one of the clearest examples of how host diet and nutrient processing by the gut microbiota shape immune responses (Kau et al., 2011). This is extensive to fish, as dietary butyrate was able to revert most of the changes induced in the intestinal transcriptome of gilthead sea bream (*Sparus aurata*) by the replacement of fish meal and fish oil with plant ingredients (Estensoro et al., 2014). However, experimental evidence in Atlantic halibut (*Hippoglossus hippoglossus*) (Murray et al., 2010), Atlantic salmon (*Salmo salar*) (Skugor et al., 2011), and Atlantic cod (*Gadus morhua*) (Morais et al., 2012a; De Santis et al., 2015) suggests that the nutritionally mediated changes in the intestinal transcriptome and function are highly fish species-specific.

Changes in the intestinal transcriptome also reflect the effect of nutritional background on the disease outcome of gilthead sea bream infected with the intestinal parasite *Enteromyxum leei* (Estensoro et al., 2011; Calduch-Giner et al., 2012). Furthermore, the high expression level of an intestinal mucin specific to the fish lineage has been proven a suitable marker of diagnostic and prognostic value of this parasitic enteritis (Pérez-Sánchez et al., 2013). In this context, the functional outline of the intestinal transcriptome of Mediterranean, Perciform, carnivorous, and gastric fish, such as gilthead sea bream and European sea bass (*Dicentrarchus labrax*), will allow comparative studies with other earlier teleostean groups, such as Salmoniformes (salmon, trout) and Cypriniformes (zebrafish, carp), which have different feeding behaviors or are stomachless (agastric fish). It would also help to understand the impact of dietary restriction, intermittent feeding, or compensatory growth (Inness and Metcalfe, 2008). In addition, this transcriptomic profiling can be a valuable tool to define the nutritional value of novel fish feeds and their effect on fish immune system.

In European sea bass, massive gene expression analysis have undertaken for gills, liver, brain, eggs, and larvae (Geay et al., 2011; Magnanou et al., 2014; Nuñez Ortiz et al., 2014; Kaitetzidou et al., 2015). A high-quality chromosome-scale genome assembly is also available (Tine et al., 2014) for this species, but in-depth analysis of the intestinal transcriptome remains to be fully addressed. Thus, the main goal of this work was to compare three different intestinal segments of European sea bass in order to generate a precise functional map of the intestine of this valuable fish species. For such purpose, transcriptomic data were generated through the construction of next-generation sequencing libraries. These sequences, combined with others from public and private repositories, were used to construct a reliable assembly nucleotide database (http://www.nutrigroup-iats.org/seabassdb) enriched on intestinal sequences, being used the annotated assembled sequences to design a custom oligo-microarray.

## Materials and methods

### Animals and tissue sampling

European sea bass of Mediterranean origin were reared under standard conditions at densities lower than 15 kg/m^3^ at indoor experimental facilities of the Institute of Aquaculture Torre de la Sal (IATS-CSIC). Photoperiod and temperature followed the natural changes at our latitude (40°5′N, 0°10′E), and fish were fed to visual satiety on 5 days per week with a commercial diet (Biomar, Palencia, Spain). At midsummer (fast growing fish), overnight fasted fish of 200 g average weight were anesthetized with 100 mg/L of 3-aminobenzoic acid ethyl ester (MS-222, Sigma-Aldrich, Madrid, Spain), bled, and samples of anterior (AI), middle (MI), and posterior (PI) intestinal segments were rapidly excised, frozen in liquid nitrogen and stored at −80°C until RNA extraction. AI was taken as a segment immediately after the pyloric caeca. MI was taken as a representative portion at the middle of the intestine. PI was taken as a segment including the rectum. All procedures were approved by the Ethics and Animal Welfare Committee of the Institute of Aquaculture Torre de la Sal according to national (Royal Decree RD53/2013) and EU legislation (2010/63/EU) on the handling of experimental animals.

### RNA extraction

Total RNA was isolated by means of the Ambion MagMax-96 for Microarray kit (Applied Biosystems, Foster City, CA, USA) after tissue homogenization in TRI reagent at a concentration of 100 mg/ml following the manufacturers' instructions. RNA quantity and purity was determined by Nanodrop (Thermo Scientific, Wilgminton, DE, USA) and Agilent 2100 bioanalyzer (Agilent Technologies, Palo Alto, CA, USA) measurements with absorbance ratios at 260 nm/280 nm above 1.9 and RNA integrity numbers between 9.2 and 10, which are indicative of clean and intact RNA.

### Transcriptome libraries sequencing and asssembly

Tissue samples from four fish were used for the construction of next-generation sequencing libraries of AI and PI segments. cDNA synthesis was performed with 700 ng total RNA by means of the MINT kit (Evrogen, Heidelberg, Germany). To increase the recovery rate of rare and unique transcripts, amplified cDNAs were normalized by duplex-specific nuclease with the Trimmer kit (Evrogen) following the manufacturer's instructions (Zhulidov et al., 2004). Normalized cDNA samples were quantified with the Quant-iT PicoGreen dsDNA quantification Kit (Life Technologies, Carlsbad, CA, USA) using a VersaFluorTM Fluorometer system (Bio-Rad, Hercules, CA, USA). Normalized cDNA (500 ng) was sheared into small fragments (250–600 nt) by nebulization with compressed nitrogen. Then, sequencing adapters were ligated to the blunt ends of the fragments, and an emulsion PCR (emPCR) was performed. After emPCR, the enriched beads were loaded onto the 454 microtiter plate and the amplicons were sequenced with a Titanium GS FLX 454 platform (Roche, Mannheim, Germany). The sff files containing all reads for each library have been deposited to NCBI Short Read Archive under accession SRR1560808.

The quality of the reads was assessed with PERL scripts developed at Lifesequencing S.L. (Valencia, Spain) for trimming of adaptors and validation of high-quality sequences. All sequences were edited to remove vector and adaptor sequences, and cleaned and filtered before assembly. Cleaning involved masking of poor-quality bases and low-complexity sequences, such as polyA tails. Filtering removed contaminating sequences (bacteria, yeast) and only high-quality sequences (*q* > 25) of more than 100 bases in length were retained. Assembly of high-quality reads was performed by means of Newbler version 2.8 program (454 Life Science-Roche) using the default parameters and the cDNA option.

### European sea bass transcriptome database construction

**Figure 1:**
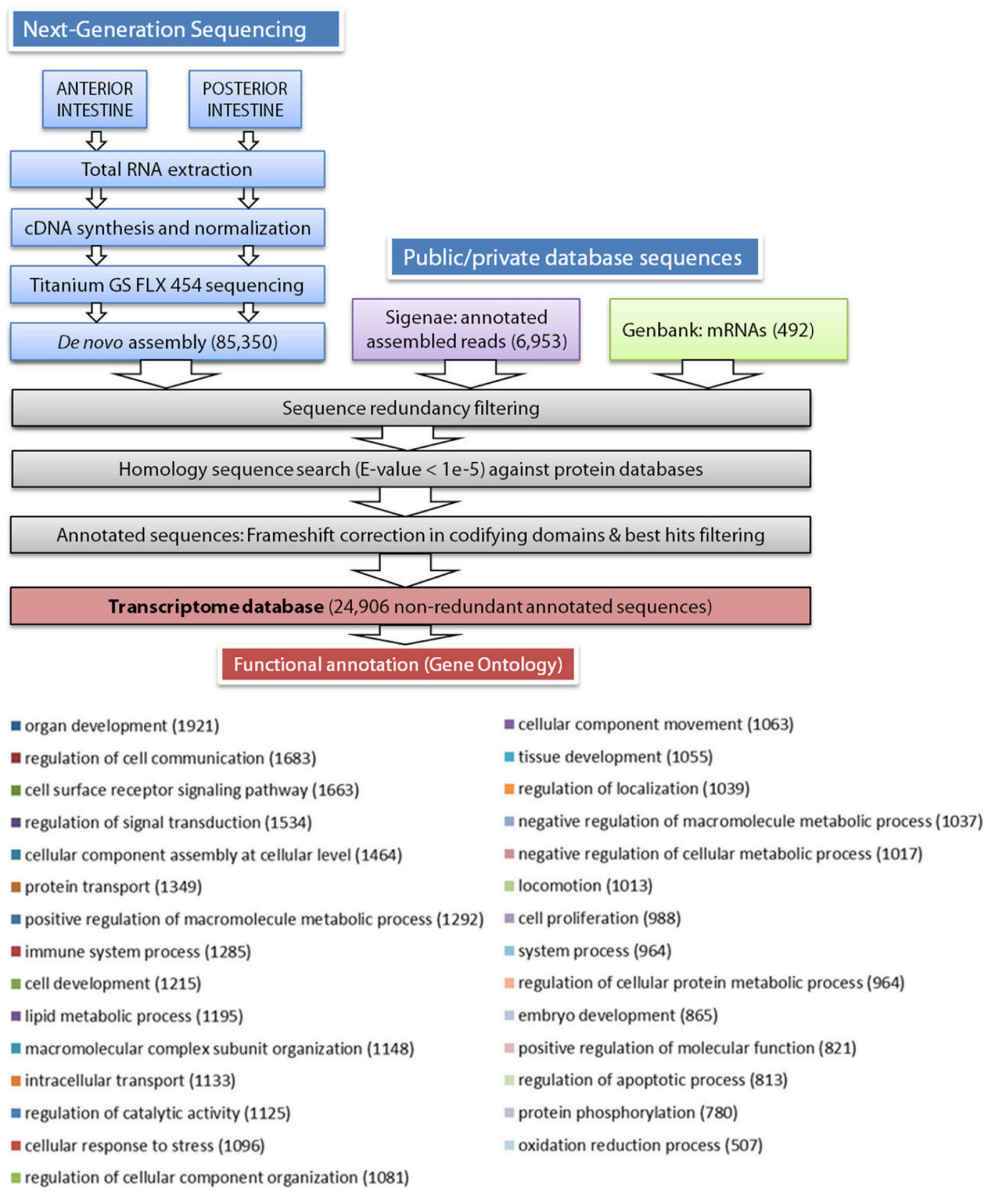
**Schematic representation of the data processing pipeline for the ***de novo*** assembly and annotation of European sea bass intestine transcriptome**. Distribution of biological process multilevel GO annotation terms in European sea bass sequences. The number of sequences for each GO term is represented between parentheses.

According to the scheme pipeline shown in Figure 1, assembled contigs and singletons from the *de novo* assembly (85,350 sequences) were combined with European sea bass annotated nucleotide sequences extracted from public (European sea bass complete mRNAs from Genbank, 492 sequences) and private (AQUAFIRST EU project at http://www.sigenae.org/aquafirst, 6953 sequences) databases. Redundant sequences were removed retaining the longest ones, and filtered sequences were annotated by similarity searches using protein reference databases, such as UniProt/Swissprot, RefSeq protein, TrEMBL, and Pfam. The *e*-value threshold to determine similarities was set to 1E-5, and the Uniprot/Swissprot entry to which they received the highest similarity was usually assigned as the gene identity. After annotation, the frameshift edition algorithm previously developed for sequencing corrections at homopolymer regions in gilthead sea bream transcriptome assemblies was applied (Calduch-Giner et al., 2013). Functional characterization by means of gene ontology analysis was made from the most representative contigs/singletons for each gene identity using the Blast2GO software (Conesa et al., 2005) with a threshold cutoff set at 1E-3. Multilevel GO term analysis was performed under default settings (minimum cutoff 1E-04 BlastX) and results were filtered by annotation score.

### Microarray construction, hybridization, and data analysis

A custom high-density oligo-microarray (8 × 15 K) from the assembled nucleotide European sea bass sequences was designed and printed using the eArray web tool (Agilent). The array comprised 60-oligomer probes for 14,147 different European sea bass annotated sequences. Total RNA (150 ng) from individuals (*n* = 8 for each intestine segment: AI, MI, and PI) were labeled with cyanine 3-CTP (Low Input Quick Amp Labeling Kit, Agilent), and 600 ng of each labeled cRNA were hybridized to microarray slides that were analyzed with an Agilent G2565C Microarray Scanner according to the manufacturer's protocol. Data were extracted using the Agilent Feature Extraction Software 11.5.1.1. Data analysis of differentially expressed (DE) genes was carried out with the Genespring GX 13.0 software (Agilent). Functional pathway analysis of DE genes was performed with the Ingenuity Pathway Analysis (IPA) software (http://www.ingenuity.com). For each gene, the Uniprot accession of the annotation equivalent for one of the three higher vertebrates model species in IPA (human, rat, or mouse) was assigned.

### Real-time qPCR validation

Up to 29 genes that were considered representative of DE genes were validated on individual AI and PI samples (*n* = 6 for each intestinal segment) by real-time qPCR, using an iCycler IQ Real-time Detection System (Bio-Rad). cDNA synthesis was performed using the High-Capacity cDNA Archive Kit (Applied Biosystems) with random decamers. For this purpose, 500 ng total RNA were reverse transcribed in a final volume of 100 μl. RT reactions were incubated for 10 min at 25°C and 2 h at 37°C. Negative control reactions were incubated in the absence of reverse transcriptase. Diluted RT reactions were conveniently used for PCR reactions in a 25 μl volume. Each PCR well contained a SYBR Green Master Mix (Bio-Rad) and specific primers (Table S1) were used at a final concentration of 0.9 μM. DNA Polymerase was activated and cDNA denatured by preincubation for 3 min at 95°C; the template was amplified for 40 cycles of denaturation at 95°C for 15 s, and annealing/extension at 60°C for 60 s. β-Actin was used as the housekeeping gene and the efficiency of PCR reactions for target and reference genes varied between 90 and 98%. The amount of product in a particular sample was determined by interpolation of the cycle threshold (Ct) value. The specificity of the reaction was verified by analysis of melting curves and by electrophoresis and sequencing of PCR-amplified products. Fluorescence data acquired during the extension phase were ultimately normalized to β-actin by the ΔΔCt method (Livak and Schmittgen, 2001). For each selected gene, fold-change variations were calculated between posterior and anterior regions.

### Statistical analysis

Microarray results for the different intestinal segments were compared in pairs by means of a *t*-test (corrected *P* < 0.05, Benjamini-Hochberg multiple testing correction).

## Results

### Transcriptome assembly and annotation

As stated in Table 1, sequencing of AI and PI segments yielded similar results in terms of the number of high-quality reads and average read length. The combined *de novo* assembly of all high-quality reads resulted in a total of 85,350 unique sequences (20,554 contigs and 64,796 singletons) with an average length of 788 nucleotides. After combination with annotated assembled reads from Aquafirst and GenBank complete mRNAs, filtering resulted in 24,906 non-redundant annotated sequences with an average length of 1050 nucleotides (N50 = 1323 nucleotides) that were uploaded to our nucleotide database (http://www.nutrigroup-iats.org/seabassdb). The assembled transcriptome yielded a total of 15,367 different gene descriptions for annotated sequences. Blast2GO analysis showed a high abundance among others of GO terms related to organ development (GO:0048513, 1921 different genes), cell communication, cell surface receptor signaling and signal transduction (GOs:0010646, 0007166, 0009966; 1683, 1663, and 1534 genes, respectively), cellular assembly (GO:0022607, 1464 genes), protein transport (GO:0015031, 1349 genes), immune system (GO:0002376, 1285 genes), cell development (GO:0048468, 1215 genes), and lipid metabolism (GO:0006629, 1195 genes) (Figure 1).

**Table 1:** **Statistics for 454 pyrosequencing libraries**.

	**Anterior intestine**	**Posterior intestine**	***De novo* assembly**
**PYROSEQUENCING READS**			
High-quality reads	492,924	494,474	987,398
Average read length (bp)	462	518	490
Total Megabases	227.9	256.3	484.3
**ASSEMBLY STATISTICS**			
Number of contigs	12,782	13,798	20,554
Reads assembled	388,694	369,029	789,688
Average contig length (bp)	1376	1426	1513
Assembled contigs N50 (bp)	1540	1539	1681
Number of singletons	48,234	48,527	64,796
Average singleton length (nt)	536	596	558
Total consensus Megabases	43.4	48.6	67.3
Average sequences coverage	7.2	6.7	10.0

**Figure 2:**
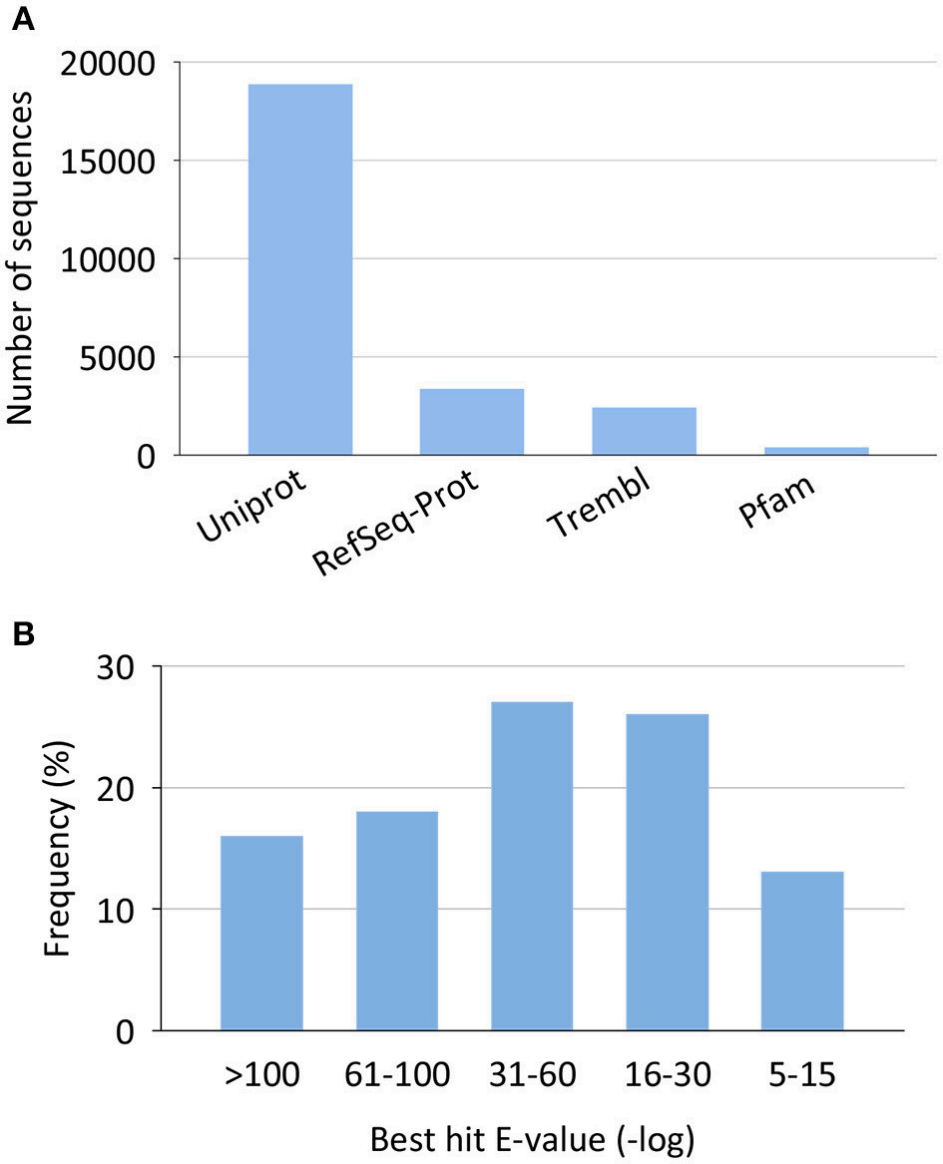
**Annotation statistics for the European sea bass transcriptome. (A)** Top annotation hits by reference databases. **(B)** Annotation best hit *e*-value distribution.

Most of the non-redundant assembled sequences had a reliable annotation against the UniProt/Swissprot database (17,181 sequences, 69%). Sequences with no significant equivalent in this database were annotated by similarity to RefSeq-Prot (4004 sequences), Trembl (3160 sequences), or Pfam (561 sequences) (Figure 2A). In all cases, reliable annotations were obtained with *e*-values lower than 1E-30 for more than 60% of sequences (Figure 2B).

### Functional analysis of differentially expressed (DE) genes

**Figure 3:**
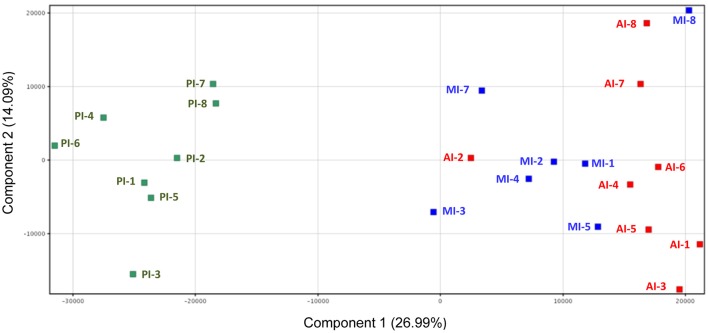
**Principal component analysis of the gene expression profile of intestine sections of European sea bass**. Anterior intestine samples are marked as AI (red), middle intestine as MI (blue) and posterior intestine as PI (green).

Microarray gene expression profiling of individual fish showed a gradient expression pattern along the intestinal tract with a close association of AI and MI by principal component analysis, with the first two components accounting for 41% of the total variance (Figure 3). Hence, *t*-tests (Benjamini-Hochberg, *P* < 0.05) were not able to detect DE genes between AI and MI samples, and these two intestinal segments were combined into one category (AI-MI) for further analysis comparing AI-MI vs. PI. This strategy yielded 5770 DE genes (Benjamini-Hochberg *t*-test, *P* < 0.05), reduced to 1906 (960 upregulated in AI-MI; 946 upregulated in PI) after filtering with a fold-change cutoff of 2.0. The complete list of DE genes with data on fold-changes is shown as Table S2.

**Figure 4:**
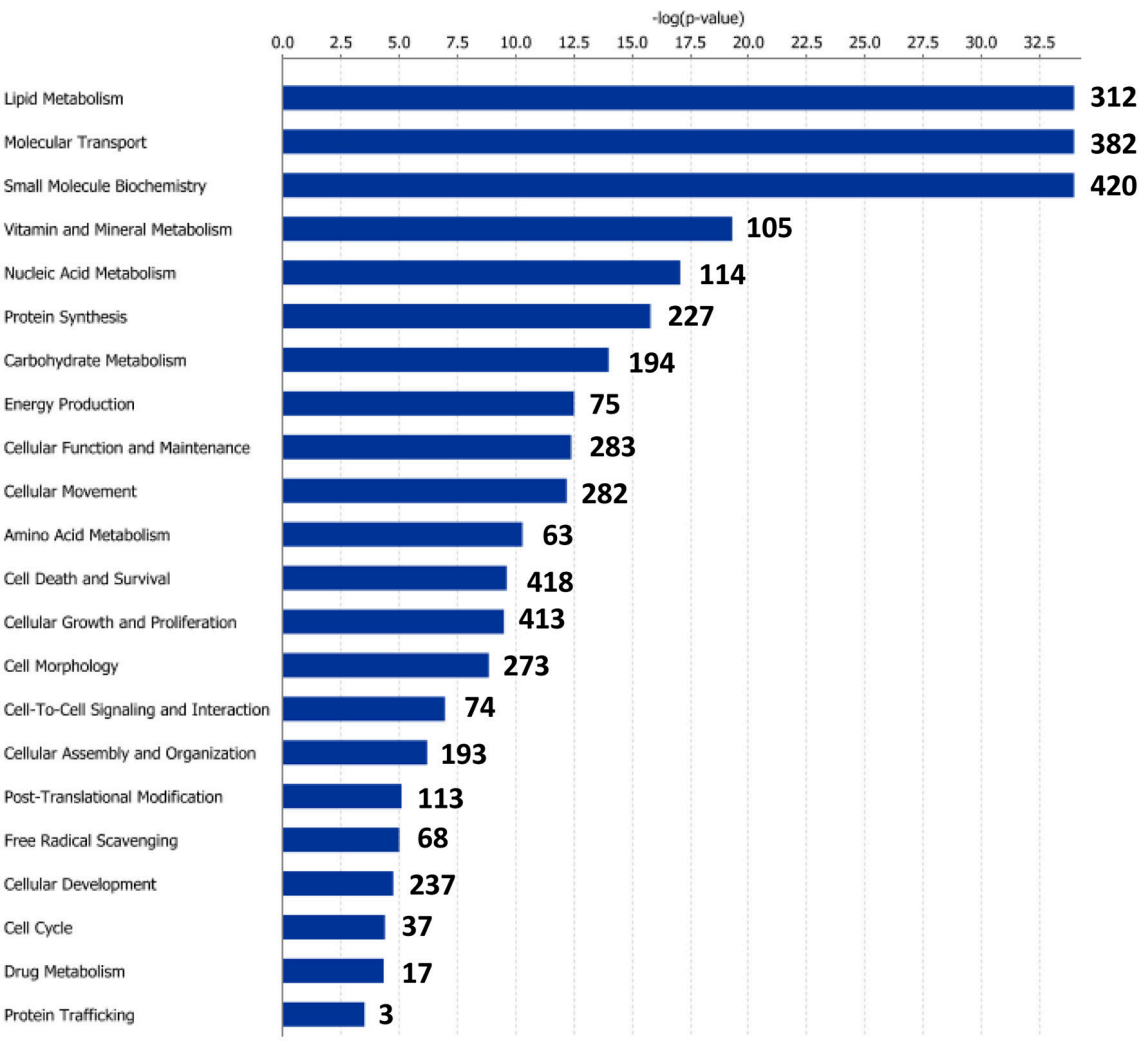
**Molecular and cellular functions of differentially expressed genes in the anterior-middle intestine segment (AI-MI)**. For a given molecular function, blue bars indicate the −log of the *P*-value, and the number of differentially expressed genes is represented.

**Figure 5:**
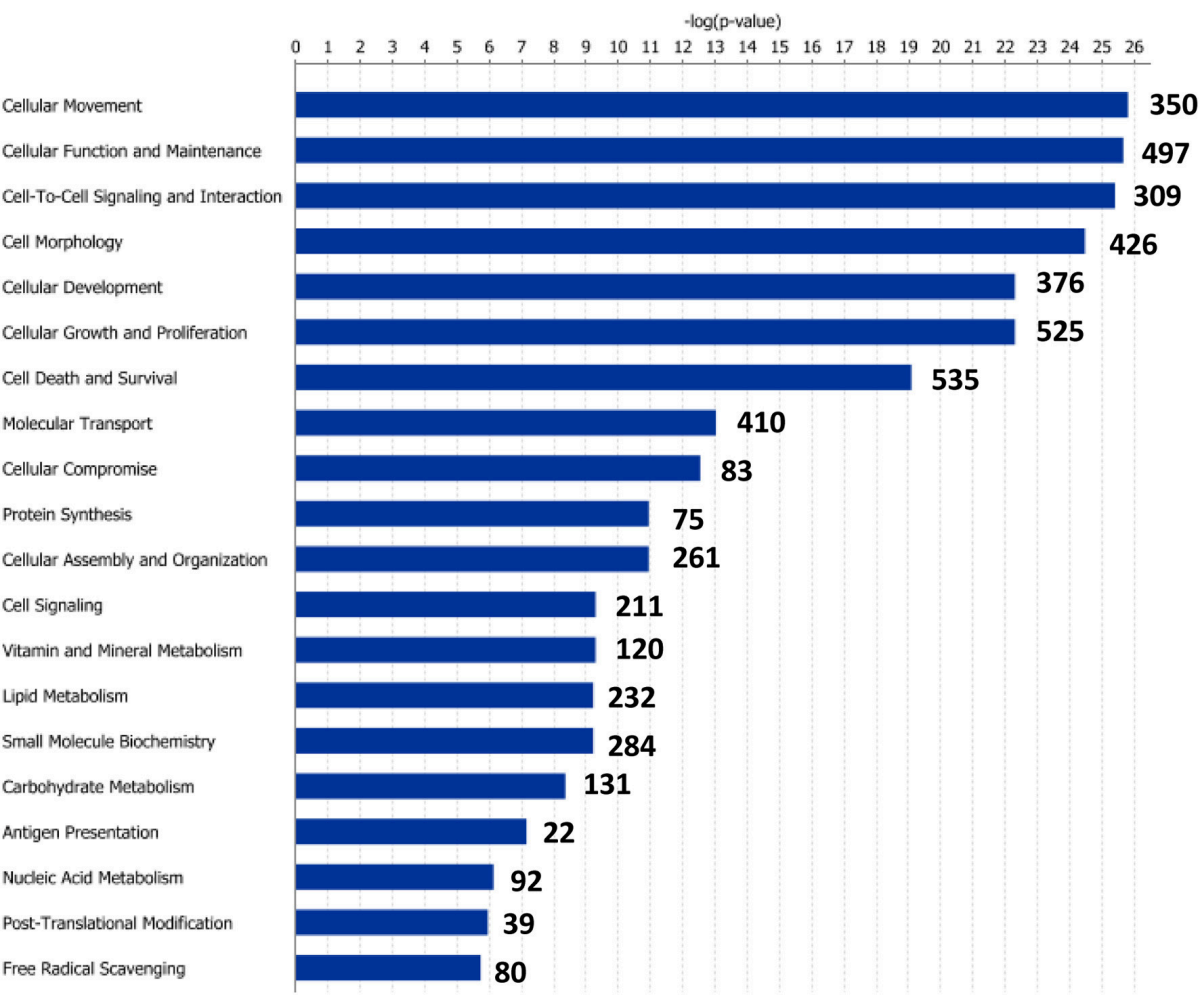
**Molecular and cellular functions of differentially expressed genes in the posterior intestine segment (PI)**. For a given molecular function, blue bars indicate the −log of the *P*-value, and the number of differentially expressed genes is represented.

**Figure 6:**
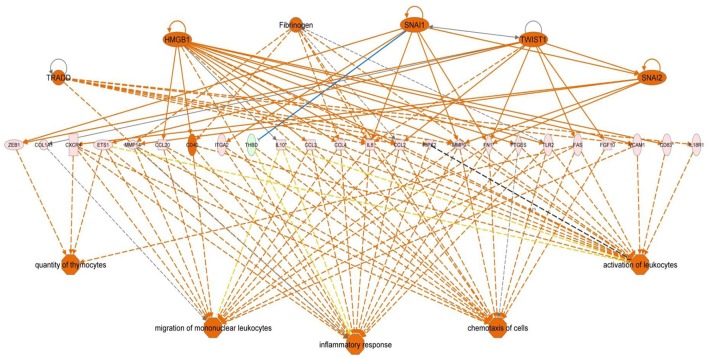
**Regulator effector network of genes differentially expressed in the posterior intestine of European sea bass**. It integrates the upstream regulators (upper line), differentially expressed genes (middle line), and downstream effects results (lower line).

**Figure 7:**
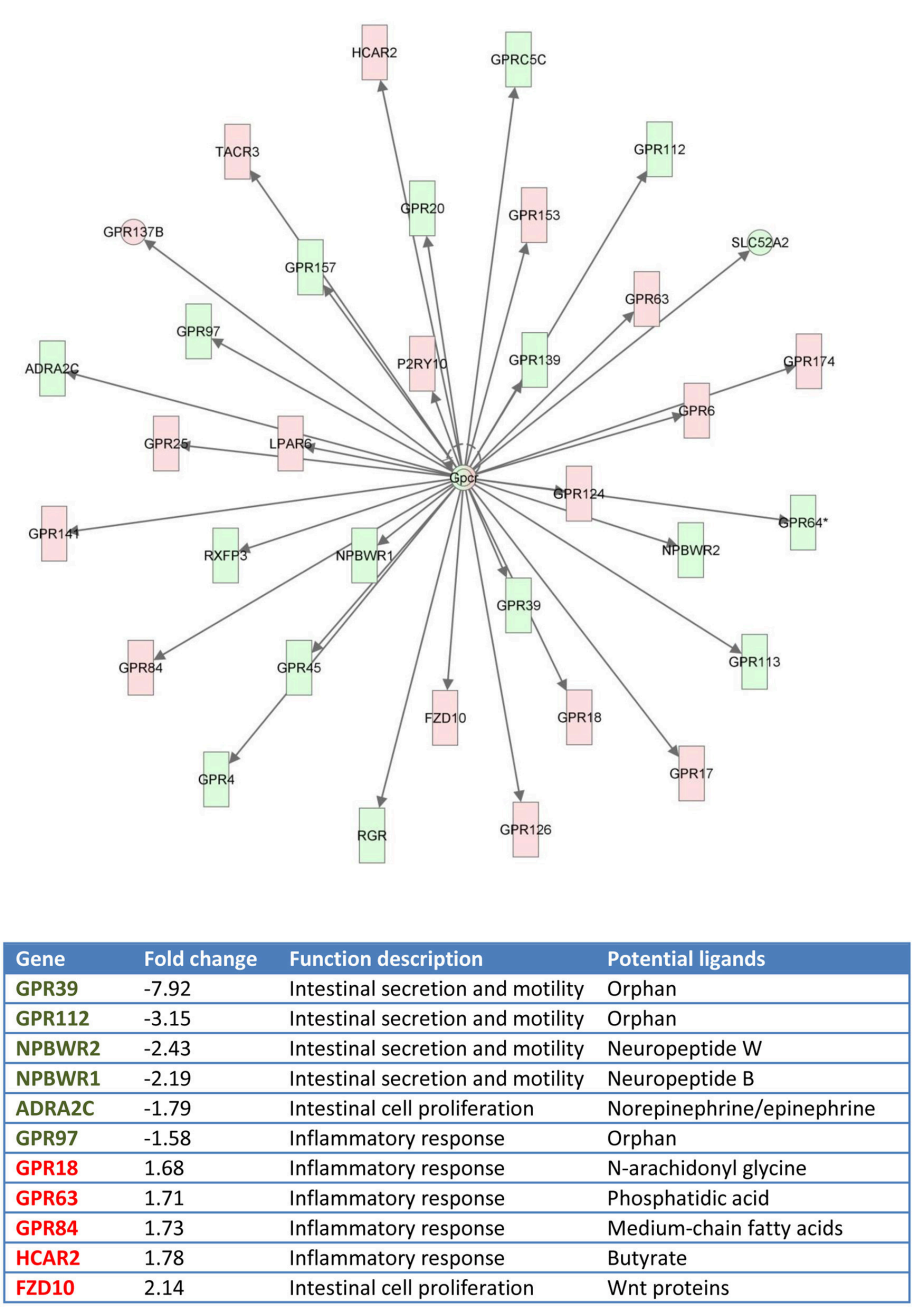
**Gene network generated by IPA analysis**. Red boxes indicate GPCRs differentially and highly expressed at posterior intestine (fold-change, expression ratio between posterior intestine and anterior-middle intestine, >1). Green boxes indicate GPCRs differentially and highly expressed at anterior-middle intestine (fold-change < 1).

Functional pathway analysis of DE genes revealed a different pattern of molecular and cellular functions throughout the intestine. In AI-MI, there was an overrepresentation of genes related to digestion and transport of nutrients (lipids, vitamins, minerals, carbohydrates, amino acids) (Figure 4). This was consistent with the observation that highly expressed genes in the AI-MI segments in comparison with PI included a vast array of proteins (*pepsin A, chymotrypsin-like elastase family member 2A, elastase-1*), lipid (*phosphodiesterase family member 7, neutral ceramidase, bile salt-activated lipase*), and carbohydrate (*lactase-phlorizin hydrolase*) digestion-related genes, as well as genes involved in lipoprotein metabolism (*14 kDa apolipoprotein, apolipoprotein C-II*) and intestinal motility (*neuromedin-B-like*) (Table 2). In PI, the most relevant molecular and cellular functions (17 out of 20) were coincident with those of AI-MI, but they were differently ranked, and the most significant ones were related to the preservation of tissue integrity and cell-to-cell communication (cellular movement, cellular function and maintenance, cell-to-cell signaling and interaction, cell morphology, cellular development, cellular growth and proliferation, cell death and survival) (Figure 5). At the same time, the list of the highest expressed genes in PI in comparison with AI-MI (Table 3) included markers of cell positional identity and epithelial barrier function (*sorting nexin-6-like, homeobox protein Hox-D4b, sorting nexin 10*). Other genes with higher expression in the PI were those related to absorption of vitamin B12 (*protein amnionless, cubilin, gastric intrinsic factor-like, transcobalamin-2*) and bile acids (*ileal sodium/bile acid cotransporter, gastrotropin*). Genes related to immune or antimicrobial response were also represented in the top list of genes with a specific or upregulated expression in the PI (*immunoglobulin-like and fibronectin type III domain-containing protein 1, transmembrane and immunoglobulin domain-containing protein 1, histidine ammonia-lyase, B-L beta chain class II histocompatibility antigen*, and *beta defensin*) (Table 3). Other immune-related DE genes in the PI, not included in the top list, were lysozymes, mieloperoxidases, and some TLRs and cytokines (Table S2). This was supported by the effector network analysis conducted by means of IPA software, which identified the immune-related effector network (24 genes connecting chemotaxis, inflammatory responses and leukocyte activation and proliferation) as one of the most distinctive gene signatures of the distal intestine (Figure 6). Likewise, cell signaling comprising a vast array of G-protein coupled receptors (GPCRs) was identified as a highly significant network (Figure 7). Interestingly, many of the receptors that were mainly expressed in AI-MI segments were related to intestinal secretion and motility (*GPR39*; *GPR112*; *alpha-2C adrenergic receptor, ADRA2C*; *neuropeptide B/W receptor types 1 and 2, NPBWR1, NPBWR2*), whereas those GPCRs with intestinal cell proliferation and inflammatory function (*GPR18*; *GPR63*; *GPR84*; *hydroxycarboxylic acid receptor 2-like, HCAR2*; *frizzled class receptor 10, FZD10*) usually had a higher expression level in the PI segment.

**Table 2:** **Filtering by fold-change of differentially expressed genes through the intestine segments**.

**Gene name**	***E*-value**	**Description**	**Fold-change**
Natterin-3-like	4.33E-71	Antimicrobial function	34,873.18
Acidic mammalian chitinase	1.74E-136	Antimicrobial function	24,297.52
14 kDa apolipoprotein	5.61E-64	Lipoprotein metabolism	10,790.68
Peptide Y	3.06E-39	Feed intake regulation	3983.42
Pepsin A	2.00E-109	Protein digestion	2556.36
Apolipoprotein C-II	1.37E-29	Lipoprotein metabolism	908.45
Uncharacterized protein LOC100002243	3.17E-36	No functional information	692.44
Meprin A subunit beta	0	Inflammatory response	594.20
Aquaporin-8	1.41E-62	Water channel protein	572.00
Hypothetical protein LOC100699670	3.64E-38	No functional information	556.59
Phosphoethanolamine N-methyltransferase 3	2.06E-147	Phosphatidylcholine synthesis	535.96
Lactase-phlorizin hydrolase	0	Carbohydrate digestion	462.75
Platelet glycoprotein 4	2.28E-129	Inflammatory response	451.42
Chymotrypsin-like elastase family member 2A	4.43E-60	Protein digestion	427.20
Alkaline phosphatase, tissue-non-specific isozyme	1.08E-126	Hydrolase enzyme	407.53
Ammonium transporter Rh type B	4.80E-113	Ammonium transporter	359.73
Protein FAM151A	1.79E-117	No functional information	356.85
Elastase-1	7.77E-106	Protein digestion	343.84
Phosphodiesterase family member 7	1.12E-143	Lipid digestion	328.98
Neuromedin-B-like	2.00E-80	Intestine motility. Inflammatory response	311.30
Ecto-NOX disulfide-thiol exchanger 1-like	3.83E-18	Cell surface oxidase	297.03
Type-4 ice-structuring protein LS-12	9.69E-31	Lipid transport	280.91
Gastrin	2.11E-25	Feed digestion	280.86
Transmembrane 4 L6 family member 1	2.64E-12	Epithelial cell adhesion and proliferation	245.05
Carbonic anhydrase 4	1.58E-45	pH buffering	218.15
Laminin N	1.03E-13	Cell adhesion and migration	206.65
Lysozyme C	1.26E-57	Immune response	206.34
Neutral ceramidase	0	Sphingolipid digestion	201.39
Bile salt-activated lipase	0	Lipid digestion	194.54
Differentially regulated trout protein	6.75E-33	Inflammatory response	182.46

*The listed genes are the most up-regulated (top 30) in the AI-MI intestine segments in comparison to PI segment*.

**Table 3:** **Filtering by fold-change of differentially expressed genes through the intestine segment**.

**Gene name**	***E*-value**	**Description**	**Fold-change**
Transmembrane protein 236	1.61E-101	No functional information	3825.29
Protein amnionless	2.11E-47	Vitamin B12 metabolism	1781.91
Immunoglobulin-like and fibronectin type III domain-containing protein 1	7.12E-81	Immune response	757.80
Transmembrane and immunoglobulin domain-containing protein 1	2.40E-24	Immune response	556.28
Cubilin	0	Vitamin B12 metabolism	513.32
Histidine ammonia-lyase	0	Immunosuppression	395.29
Cathepsin L-like	1.52E-45	Cysteine proteinase	367.05
B(0,+)-type amino acid transporter 1	0	Cysteine transporter	314.06
Unconventional myosin-Vb	6.06E-82	Cell organelle movement	229.39
Beta-defensin	5.11E-20	Antimicrobial peptide	157.27
Ileal sodium/bile acid cotransporter	9.66E-107	Bile acid metabolism	125.59
Gastric intrinsic factor-like	5.44E-38	Vitamin B12 metabolism	124.94
Homeobox protein Hox-C13a	1.10E-158	Vasoactive intestinal peptide	118.63
Excitatory amino acid transporter 3	0	Amino acid transporter	116.11
Homeobox protein Hox-A13a	6.05E-113	Vasoactive intestinal peptide	113.58
Patched domain-containing protein 3	1.96E-159	Proteosomal degradation	105.85
Forkhead box protein D2	2.31E-87	Probable transcription factor	95.66
Sorting nexin 6-like	1.07E-159	Epithelial barrier function	55.27
Gastrotropin/ileal fatty acid binding protein/FABP6	6.49E-35	Bile acid metabolism	52.48
Solute carrier family 15 member 2	0	Oligopeptide transporter	52.26
Zinc transporter 8	5.22E-96	Zn metabolism	51.39
Homeobox protein Hox-D4b	6.20E-27	Positional identity	50.83
Phosphatidylinositol 3,4,5-trisphosphate 3-phosphatase TPTE2	2.87E-128	Regulation of AKT/PKB pathway	50.50
Granulins-like	3.87E-26	Cell growth regulation	48.21
Sorting nexin 10	8.17E-17	Epithelial barrier function	45.71
Major facilitator superfamily domain-containing protein 4-A	2.82E-157	Small molecules transport	42.50
Hydroxylysine kinase	3.23E-129	Amino acid catabolism	42.33
Transcobalamin-2	1.43E-37	Vitamin B12 metabolism	42.14
Homeobox protein Hox-A11a	1.23E-15	Positional identity	41.07
Class II histocompatibility antigen, B-L beta chain	7.74E-17	Immune response	38.13

*The listed genes are the most up-regulated (top 30) in the PI intestine segment in comparison to AI-MI intestine segments*.

### Real-time qPCR validation

**Figure 8:**
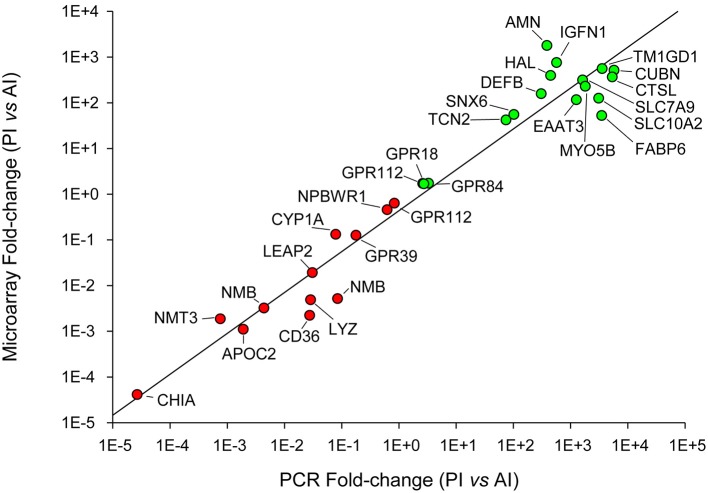
**qPCR validation of microarray results**. Correlation plot of fold-change (posterior/anterior) values for the selected genes analyzed by qPCR (X-axis) and microarray (Y-axis).

Selected genes for qPCR validation of microarray results were representative of the main processes along the intestinal tract: (i) amino acid transport and proteolysis [*B(0*,+*)-type amino acid transporter 1, excitatory amino acid transporter 3, cathepsin L*], (ii) lipid metabolism and digestion (*apolipoprotein C-II, bile salt-activated lipase, phosphoethanolamine-N-methyltransferase 3*), (iii) integrity of the epithelial barrier (*sorting nexin 6*), (iv) antimicrobial action (*acidic mammalian chitinase, beta-defensin, liver-expressed antimicrobial peptide 2, unconventional myosin-Vb*), (v) inflammatory and immune responses (*histidine ammonia-lyase, immunoglobulin-like and fibronectin type III domain-containing protein 1, lysozyme C, neuromedin-B, platelet glycoprotein 4, transmembrane*, and *immunoglobulin domain-containing protein 1*), (vi) intestinal chemosensation (*GPR18, GPR39, GPR63, GPR84, GPR112, NPBWR1*), and (vii) metabolism of bile acids (*gastrotropin, ileal sodium/bile acid cotransporter*) and vitamin B12 (*cubilin, protein amnionless, transcobalamin-2*). A scatter plot of microarray and qPCR fold-change values for all the above-mentioned genes highlighted a strong linear correlation (*r* = 0.97) near to equality (Figure 8).

## Discussion

In the present work, the assembly and annotation of 454 normalized libraries resulted in a transcriptome that, in terms of non-redundant contigs (85,350) or different annotated sequences (15,367), is similar to those previously reported for other tissues in this fish species. The resulting information is accessible through an easy-to-use web interface hosted at http://www.nutrigroup-iats.org/seabassdb that contains data from assembled, annotated and semi-automatically curated singletons/contigs. This database has been built as previously reported for gilthead sea bream (http://www.nutrigroup-iats.org/seabreamdb) (Calduch-Giner et al., 2013), and offers the possibility for data requisition by several Blast options or direct word searches for annotated names or gene ontology terms. These genomic resources are useful tools for functional phenotyping of Mediterranean farmed fish and have been used to develop several pathway-focused PCR arrays for the simultaneous and semi-automated profiling of selected markers of mitochondria function and biogenesis (Bermejo-Nogales et al., 2014, 2015), lipid metabolism (Benedito-Palos et al., 2013, 2014), immune response (Pérez-Cordón et al., 2014; Pérez-Sánchez et al., 2015), and muscle growth (Benedito-Palos et al., 2016) in gilthead sea bream, and lipid metabolism in European sea bass (Rimoldi et al., 2016). In the current study, this knowledge has been used to design and validate a high density oligo-microarray, enriched in actively transcribed genes of the intestinal tract.

The advent of transcriptome analysis by RNA-seq has revolutionized the field of fish transcriptomics with the discovery of new transcripts, interrogation of post-transcriptional modifications and analysis of single nucleotide polymorphism (Louro et al., 2014; Qian et al., 2014). However, DNA microarray technology still constitutes a solid and widely used approach because of its competitiveness in terms of cost, sample preparation, and interpretation of data, despite of their methodological limitations including the dependence on the existing knowledge on expressed sequence tags or genomic sequences (Sedighi and Li, 2014; Martin et al., 2016). Indeed, the current custom European sea bass microarray was able to evidence a pronounced transcriptional divergence of PI in relation to AI-MI segments, which was validated by qPCR of 29 selected genes representative of the main biological process and functions across the intestine. The magnitude of the transcriptional difference (5770 genes, *P* < 0.05; 1906 with a fold-change cutoff = 2) between the intestinal segments found in the current study is higher than that reported in most of the studies dealing with changes induced in fish intestine by different experimental approaches. Table 4 shows the number of DE intestinal genes in fish with different feeding habits, exposed to environmental stress, pathogens, or diet interventions. Although comparisons across studies in different species is always difficult, transcriptome differences found between the two intestinal segments are of the same order than those found when comparing different organs and tissues, either in fish or other animal models. To put it in a proper perspective, the intestinal segment differences found in European sea bass are comparable to those found among heart and red and white skeletal muscle in gilthead sea bream, with more than 3000 DE genes (Calduch-Giner et al., 2014). Also, the level of DE probes among two different tissues in gene expression atlas of human and other model organisms is in the range of 50–90% (Son et al., 2005; Briggs et al., 2011; Freeman et al., 2012; Kapushesky et al., 2012).

**Table 4:** **Fish transcriptomic profiling studies for the characterization of gut responses to environmental stress, pathogens, or diet interventions**.

**Feeding habit**	**Fish species (Order)**	Intestinal segment^*^	**Transcriptomic technique**	**Experimental approach**	**Experimental period**	**DE genes**	**References**
Carnivorous	*Sparus aurata* (Perciformes)	Posterior	Microarray	Parasite infection × diet intervention	102 days	359–1392	Calduch-Giner et al., 2012
		Posterior	Microarray	Parasite infection	113 days	371	Davey et al., 2011
	*Dicentrarchus labrax* (Perciformes)	Hind gut	RNA-seq	Bacterial oral vaccination	135 days	366	Sarropoulou et al., 2012
	*Lates calcarifer* (Perciformes)	n.a.	RNA-seq	Salinity exposure	3 days	1228	Xia et al., 2013
		n.a.	RNA-seq	LPS injection	8 days	1739	Xia et al., 2013
		n.a.	RNA-seq	Bacterial injection	8 days	1477	Xia et al., 2013
		n.a.	RNA-seq	Fasting	8 days	1080	Xia et al., 2013
	*Scophthalmus maximus* (Pleuronectiformes)	Pyloric caeca	RNA-seq	Parasite infection	42 days	1413	Robledo et al., 2014
	*Salmo salar* (Salmoniformes)	n.a.	Microarray	Diet intervention	55 weeks	1409	Morais et al., 2012b
		n.a.	Microarray	Genotype	55 weeks	1626	Morais et al., 2012b
		Distal	Microarray	Diet intervention	8 weeks	33–254	Król et al., 2016
		Distal	Microarray	Diet intervention	1, 2, 3, 5, 7 days	7–48	Sahlmann et al., 2013
		Distal	Microarray	Diet intervention	12 weeks	2664	De Santis et al., 2015
	*Oncorhynchus mykiss* (Salmoniformes)	n.a.	Microarray	Viral oral vaccination	4 weeks	305	Doñate et al., 2010
	*Oryzias latipes* (Beloniformes)	n.a.	RNA-seq	Salinity exposure	1 h, 3 h, 1 day, 7 days		Wong et al., 2014
	*Danio rerio* (Cypriniformes)	Entire	RNA-seq	Diet intervention	21 days	328	Rurangwa et al., 2015
	*Ictalurus punctatus* (Siluriformes)	Entire	RNA-seq	Bacterial infection	3 h, 24 h, 3 days	693–1035	Li et al., 2012
Herviborous	*Oreochromis niloticus* (Perciformes)	Anterior	RNA-seq	Salinity exposure	4 weeks	AI: 726	Ronkin et al., 2015
		Posterior				PI: 636	
	*Oreochromis mossambicus* (Perciformes)	Anterior Posterior	RNA-seq	Salinity exposure	4 weeks	AI: 474 PI: 600	Ronkin et al., 2015
	*Ctenopharyngodon idella* (Cypriniformes)	n.a.	RNA-seq	Diet intervention	70 days	2119	Li et al., 2015
		n.a.	RNA-seq	Viral infection	2–120 h	500 to > 1500	Shi et al., 2014
Omnivorous	*Pangasianodon hypophthalmus* (Siluriformes)	n.a.	RNA-seq	Salinity exposure	8 weeks	1013	Nguyen et al., 2016
	*Gadus morhua* (Gadiformes)	Mid gut	Microarray	Diet intervention	12 weeks	289	Morais et al., 2012a

* *The nomenclature used in each reference has been kept; n.a., not available*.

At the morphological level, differences across the intestinal length have been described in teleosts, with a progressive decrease in the diameter, the density of mucosal foldings and goblet cells (main cell producers of mucus), whereas rodlet cells closely linked to osmoregulation and the immune system become more abundant in the posterior segment (Reite, 2005; Ballester-Lozano et al., 2015). In European sea bass, a different profile of digestive enzyme activities between the anterior and the posterior segments has been shown, being the activity of amylase, alkaline phosphatase, total alkaline proteases, and trypsin higher at the AI segment (Castro et al., 2016). Functional and transcriptional compartmentalization of the digestive tract has also been described in zebrafish (*Danio rerio*), a stomachless teleost, with 2558 DE genes across the seven segments considered in that study (Wang et al., 2010). Thus, the fish intestine has evolved as a complex tissue with a gradual change in nutrient absorptive capacity from AI to PI segments, whereas the posterior segment appears as part of the first line of defense against pathogens (Løkka et al., 2014). The results reported herein indicate that this change is more gradual than initially envisaged, as some immune-relevant genes are much more expressed in the AI-MI segments than in PI, such as *natterin-3-like* and *acidic mammalian chitinase*. Natterin-like proteins contain a mannose-binding lectin-like domain that might act as a pathogen recognition protein with an important role in the acute-phase response of fish (Magnadóttir, 2006). Chitinases are primarily associated with the stomach, but they have also been found in the intestine with a role in food digestion and immune defense in several vertebrate species, including fish (Gutowska et al., 2004; Tran et al., 2011). Moreover, administration of recombinant chitinases boosts several serum immune parameters in orange-spotted grouper (*Epinephelus coioides*) (Zhang et al., 2012). All these results point out the importance of antimicrobial function also in this intestinal portion. On the other hand, the PI also plays a key role in the reabsorption of bile salts and some micronutrients. This dualism is, thereby, of relevance when considering the regulation of the intestine as whole, as further discussed below for some particular genes or biological processes.

Fish intestine has been reported to progressively tighten from the anterior to the posterior part, and intestinal integrity and intercellular permeability is maintained by proteins, such as integrins, claudins, cadherins, and gap junction proteins (Tsukita et al., 2001; Lu et al., 2013). Genes encoding these proteins were largely represented in our microarray, although no clear distinctive differential expression pattern was detected with the established cutoff value. However, the expression profile of these intestinal markers was quite similar to that reported in gilthead sea bream (Pérez-Sánchez et al., 2015). This finding suggests that the structure and function of tight junctions is maintained through the evolution of modern fish species, although some species differences can occur even when comparing close-related fish.

Functional network effector analysis clearly evidenced that active immune surveillance is a key role of the distal intestine of European sea bass, in comparison with the role of mammalian large intestine. This is probably due to fact that fish are continuously and directly exposed to a microbial-rich environment; e.g., 1 liter of marine water contains 10,000 million virus and 9000 million of bacteria (Fenical, 1993; Fuhrman, 1999). Thus, compared to terrestrial animals, they have to cope with high microbial loads. The immune cell repertoire of teleost intestine is governed by the gut-associated lymphoid tissue, and regional differences have already been reported (reviewed in Rombout et al., 2011; Salinas and Parra, 2015). In European sea bass, the predominance of T cells in the PI has been long established (Abelli et al., 1997), and the intestinal regionalization of the expression of T cell-markers was suggested for the first time in a teleost in this marine fish (Picchietti et al., 2011). Our present results confirm and expand these data with over-representation of several genes related to cell-mediated intestinal immunity in the PI segment. Similarly, a marked increase in neutrophil density was observed in intestinal segment 6 of zebrafish, in coincidence with an increase of the neutrophil-expressed genes and several cytokines implicated in intestinal immunity (Wang et al., 2010). *Cathepsin L-1* also had the highest expression in segment 6, as *cathepsin L-like* in the PI of European sea bass. Cathepsin L family has multifunctional roles in many biochemical pathways of vertebrates, including intracellular protein degradation, antigen presentation, and cellular development (Zhou et al., 2015). Nevertheless, their function in fish is just starting to be elucidated and further studies are needed to reveal if they have an immunological role in the intestine of fish. In addition, infection models in a wide-range of fish species have shown the importance of the local intestinal immune response (Estensoro et al., 2012; Li et al., 2012; Pérez-Cordón et al., 2014; Dezfuli et al., 2015). In our study, the transcriptome of MI did not differ from that of AI, however, in a quantitative PCR analysis of 27 antimicrobial genes (Oehlers et al., 2011), the MI exhibited elevated expression of *dual oxidase*, the *defensin beta-like*, and peptidoglycan recognition protein families, and also presented the highest numbers of leukocytes and endocytic cells, supporting a specialized immunological role. In agreement with this, *beta-defensin* was among the top DE genes in the PI of European sea bass.

As expected, the highest expression level of genes related to digestion was observed in the AI-MI segments, since an absorption gradient is present along the length of the intestine of all fish. Water-soluble nutrients are mostly absorbed in the pyloric caeca and the midgut (Sundell and Rønnestad, 2011) and the majority of lipids are absorbed in these first segments with the concurrence of secreted bile salts to hydrolyse triglycerides to free fatty acids and glycerol. In coincidence with data from zebrafish (Wang et al., 2010), several well-known molecular markers of mammalian small intestine, *fabp2, apoa1*, and *vil1l*, had a higher expression at the AI-MI segments than at PI in European sea bass (Table S2). Furthermore, we also found that the gene markers (*fabp2* and *transcription factor gata5*) found to be stable across the five most proximal segments of adult zebrafish (Oehlers et al., 2011), were higher in AI-MI segments than in the PI one. Typically, bile acids continue along the whole intestine and are absorbed by enterocytes of the PI segment and are transported to the liver as a part of the enterohepatic circulation. This process ensures the rapid turnover of bile acids by the participation of gastrotropin (also termed *ileal lipid-binding protein or fatty acid-binding protein 6, fabp6*), which is exclusively expressed in the intestine of mammals (Gong et al., 1994; Fujita et al., 1995; Besnard et al., 2002). The higher expression of *gastrotropin* in the PI segment of European sea bass is consistent with previous results in zebrafish (Alves-Costa et al., 2008; Oehlers et al., 2011) and gilthead sea bream (Pérez-Sánchez et al., 2015), in which the detection of *gastrotropin* transcripts was exclusive to the distal portions of intestine. The enterohepatic circulation also serves to efficiently use the water-soluble vitamin, vitamin B12. This vitamin plays a key role in the normal functioning of the brain and nervous system, and it is synthesized exclusively by bacteria in the gut (Raux et al., 2000). Deficiency of this vitamin results in decreased growth, anemia and abnormal fish behavior (NRC, 2011), and the dietary replacement of fish meal by plant proteins is accompanied by an increased risk of vitamin B12 deficiency in Atlantic cod (Hansen et al., 2007). The underlying regulatory mechanisms remain to be established in fish, but the observed high expression of vitamin B12 binders (*gastric intrinsic factor-like protein, transcobalamin-2*) and binder-vitamin complex receptors (*protein amnionless, cubilin*) in the PI of European sea bass ensures an efficient intestinal uptake and absorption of this essential micronutrient.

Our results also indicate a different expression pattern of GPCRs throughout the AI-MI and PI. GPCRs are the largest family of signaling receptors in vertebrates and most of them are ubiquitously expressed in the enteroendocrine cells of the GI tract. The presence of these receptors along the gut constitutes a key gastrointestinal chemosensory system for the regulation of appetite, nutrient digestion, intestinal motility, and mucosal defense mechanisms, contributing to the integration of a vast array of pathways linking the gut with the brain and metabolically active tissues (Reimann et al., 2012). In this study, the GPCR with the highest intestine spatial regulation (upregulated in the AI-MI) was *GPR39*. This is an orphan receptor that belongs to the ghrelin/motilin receptor subfamily, and it is emerging as an important regulator of gastrointestinal motility and secretion (Depoortere, 2012). Two forms of *GPR39* have been characterized in black sea bream (*Acanthopagrus schlegeli*), and intestinal expression of *sbGPR39-1a* was found to decrease significantly during food deprivation (Zhang et al., 2008). Another orphan receptor, *GPR112*, was highly DE in the AI-MI of European sea bass. This GPCR was also highly expressed in the intestinal mucosal layer of zebrafish (Harty et al., 2015) and rodents (Ito et al., 2009; Badiali et al., 2012). *NPBWR1* and *NPBWR2* were also differentially upregulated in the AI-MI of European sea bass. Neuropeptide B and neuropeptide W are both endogenous peptide ligands for NPBWR1 and NPBWR2, which are key players in the regulation of feeding and energy metabolism (Mondal et al., 2003; Tanaka et al., 2003). These neuropeptides are mostly expressed in the brain, although a high expression of *neuropeptide B* has also been found in the intestine of medaka (*Oryzias latipes*) (Hiraki et al., 2014). Another GPCR with a high level of expression in the AI-MI of European sea bass was *ADRA2C*, a known ligand for norepinephrine and epinephrine (Lefkowitz and Caron, 1988). The regulatory role of alpha adrenergic receptors in the proliferation of intestinal epithelial crypt cells is well known in mammals (Schaak et al., 2000) and their tissue expression patterns parallel those of their mammalian orthologs in zebrafish (Ruuskanen et al., 2005) and perhaps, European sea bass.

Regarding the upregulated GPCRs in the PI segment, many of them were involved in cell proliferation and immune response. The exception was the orphan receptor *GPR97*, with a lowered expression in the PI of European sea bass despite its proven role in the remodeling of lymphatic cells of the mouse intestine (Valtcheva et al., 2013). This contrasted with the observation that *FZD10*, a receptor for molecules in the Wnt pathway that is a good marker of colorectal tumors in humans (Nagayama et al., 2009), was the most upregulated GPCR in the PI of European sea bass. Another highly and differentially expressed GPCR at PI was the *hydrocarboxylic acid receptor 2*, also termed *GPR109*. This receptor has anti-inflammatory properties acting as the metabolite sensor of butyrate, the end product of the microbial fermentation of dietary fiber (Singh et al., 2014). This short-chain fatty acid has been considered a promising feed additive in aquaculture, improving the immunological status and intestinal condition in carp (*Cyprinus carpio*) (Liu et al., 2014). Recent data also support the promising effects of butyrate supplementation in European sea bass, and Rimoldi and co-workers have found reported improved growth and increased gene expression of the *oligopeptide transporter 1* (*PEPT1*) in the hindgut of fish fed low-fish meal diets (unpublished). Other GPCRs that showed increased expression levels in the PI of European sea bass were *GPR84, GPR63*, and *GPR18*. GPR84 is a putative receptor of medium-chain fatty acids that mediates proinflammatory responses in human myeloid cells (Suzuki et al., 2013), whereas GPR63 binds phosphatidic acid and is also involved in the inflammatory response (Kostenis, 2004). Lastly, *GPR18*, also known as N-arachidonyl glycine receptor, is ubiquitously expressed in channel catfish (*Ictalurus punctatus*) tissues, and its immunostimulatory action in response to infection with *Aeromonas hydrophila* has been proven (Pridgeon and Klesius, 2013).

In summary, the functional phenotyping at the molecular level of the intestinal tract of European sea bass has been assessed with a specific oligo-microarray. Similar molecular signatures were found for AI and MI segments, that were clearly different to PI one. The consistency of this finding was highly supported by qPCR results for the genes selected as representative markers of several functions or processes, including intestinal permeability, macronutrient and micronutrient digestion and absorption, immune response and intestinal chemosensing. With the available transcriptomic data, we can match the AI-MI segments of European sea bass to the small intestine of mammals and the first segments of zebrafish (segments 1–5 as defined in Wang et al., 2010, 1–4 as defined in Oehlers et al., 2011), whereas the PI of European sea bass would correspond to the large intestine of mammals and last segments of zebrafish (segments 6–7 as in Wang et al., 2010, or segment 6 as in Oehlers et al., 2011). In any case, fish gut transcriptome should not be considered a static feature, and significant changes and evolution could be envisaged not only in a spatial basis, but also related to changes in season and feeding regimes. In this sense, further work is necessary, not only in European sea bass, but in other fish species of interest, to establish the precise tuning of intestine plasticity from a transcriptional and functional perspective.

## Author contributions

AS and JP conceived and designed the experiment. JC sampled animals and performed RNA extractions. JC and JP analyzed microarray data. JC, AS, and JP wrote the manuscript.

## Funding

This work was funded by the EU seventh Framework Programme by the ARRAINA (Advanced Research Initiatives for Nutrition and Aquaculture; KBBE-2011-288925) project. It does not necessarily reflect the views of the EU and in no way anticipates the Commission's future policy in this area. The funders had no role in the study design, data collection and analysis, decision to publish, or preparation of the manuscript. Additional funding was obtained from the Spanish Ministerio de Economía y Competitividad through the MI2-FISH project (Unraveling Metabolic, Intestinal and Immunopathological Fish Status; AGL2013-48560) and Generalitat Valenciana (PROMETEO FASE II-2014/085).

### Conflict of interest statement

The authors declare that the research was conducted in the absence of any commercial or financial relationships that could be construed as a potential conflict of interest.
