# Supplementary material for: Gene Expression Profiling Reveals Functional Specialization along the Intestinal Tract of a Carnivorous Teleostean Fish (Dicentrarchus labrax)
Source: Front Physiol. 2016 Aug 25;7:359. doi: 10.3389/fphys.2016.00359 (PMC4997091; doi:10.3389/fphys.2016.00359)
Supplement: Supplementary file 1 [file Table1.PDF]

TABLE S1. Forward and reverse primers for real-time PCR.

Gene name	Symbol		Primer sequence
B(0,+)-type amino acid transporter 1	SLC7A9	F	CAA CTG GGC ACG CAA ACT CA
		R	CTC AGG AGG CAC TAC TTC CAT CAG
Cathepsin L	CTSL	F	CAA GTT TGA AAG GGT CTA CAG CTC TCC AT
		R	AGT TTG CGG TTG TTG AGC CAG ATT T
Cubilin	CUBN	F	GCA GCG ATT CTG TGG TAC TGT ATC
		R	GTC ATG GTG TTG GCA GAG GAG
Excitatory amino acid transporter 3	EAAT3	F	TGG TGA CGG TGC TGA GTG GT
		R	GAA GAT GAG TGG CAG GCA GAT GGT
Gastrotropin	FABP6	F	ATA CCT TCT CCT GGA CCC AAA TCT ACC C
		R	TCG CAC TCC TTG CTG ATG GTG AAT T
Histidine ammonia-lyase	HAL	F	TTC AAC GCT TCC TGC CTC TCC TT
		R	GGT CTC CAC TAG CAC CGA CTG TT
Ileal sodium/bile acid cotransporter	SLC10A2	F	GCC GTC GTC ATC ATC ATT ATG G
		R	CGT CCA GCC AGT AGC AGA T
Immunoglobulin-like and fibronectin type III domain-containing protein 1	IGFN1	F	ATG ACC GCC TCC ACT ACA CA
		R	TTA TTG AAG AGC CTG TCA GCC ACT
N-arachidonyl glycine receptor	GPR18	F	GCT CTG TGG CTG TTT GCT CTG
		R	GTG CTT TGG CTG GAC GAT GG
Probable G-protein coupled receptor 63	GPR63	F	CGT GCC TTC ACC ACC ATC CTC ATC C
		R	GCC CAG CAC ACT GTA AAC ACG GAG AA
Protein amnionless	AMN	F	CAC TGC CTC CGC TGC CAT CT
		R	GCT CTC CAA CAT CAC TGT GTC TCT TCA AG
Sorting nexin-6	SNX6	F	CTG GAT CTG GAG TCA CGC TAC C
		R	CAT TCC TCC TGG CGG TCT CTA AC
Transcobalamin-2	TCN2	F	AAG TGC GGT GAA TGG AGA GC
		R	CAT CAG AGG AGA GGT GCC AAT ATC
Transmembrane and immunoglobulin domain-containing protein 1	TMIGD1	F	GTC AGA GCA GGT TAC AGT AGA AGA AGA G
		R	ACG GAT GAG ACT GGC GGA TT
Unconventional myosin-Vb	MYO5B	F	GTG TCT TGA TCC GTG CCC TGA GTA ACC
		R	AGT GAT GGA GAG GAG CTG CTG ATA GG
Acidic mammalian chitinase-like	CHIA	F	AGA CTC TGC TTG CTG TTG GTG GAT GGA
		R	TGA CGG TTG GCT GGA GTG GAC A

Apolipoprotein CII	APOC2	F	GCG AGT GGA TAC CTG GAA AGC A
		R	GTC GTT CAG AAT GCC AGC GTA GG
Bile salt-activated lipase	CEL	F	CTG CCA ACG CCA CGA CTG
		R	TGG GCT GAG AGA AGA GGT AGG A
Cytochrome P450 1A1	CYP1A	F	CAG GTG GTG GGC AGC GGT AA
		R	GGC AGG AAT TGA AGA ACA GGG ATG AAG TC
G-protein coupled receptor 39	GPR39	F	CAC CTC ACC ATC GAA CAC ATC AAC A
		R	AGG CGG CGT GGG AGT TAC
Liver-expressed antimicrobial peptide 2	LEAP2	F	GGT TTG CTC CAA CGG ACC AA
		R	CAC AGG CTT CAT GCT GTT CCA
Lysozyme C	LYZ	F	CGG AGC CAT CAA CCA CAA CAC TG
		R	GCC ATT ATT ACA CCA CCA GCG ACT GT
Neuromedin-B	NMB	F	AGT TCC TGC GAG TGG CGT TG
		R	CGA GCG GCT CTC TTG TGT ATC C
Neuropeptides B/W receptor type 1	NPBWR1	F	TGG CTC TGA CGA CGG ACC TGA
		R	TAG GAG ATC CCG ATT AGC AGT GGT GTG
Phosphoethanolamine N-methyltransferase 3	NMT3	F	GCT ACC GCA CTG ACG CAC AAT ACA
		R	CCT CTG GCT CCT CCA CTT GTA CTG AT
Platelet glycoprotein 4	CD36	F	GGA GAG CAA GGA TCT GAA AGG GAT TGA AGT
		R	AGG GTA TTT GGC TGG AGG GCG TAG
Beta-defensin	DEFB	F	GGG CTG AGC TTG GTT CTC CTT GT
		R	CCT CCC CAA CTG CGA GCA TCA
G-protein coupled receptor 84	GPR84	F	TGT TGA CCA TTC TAG CCT TCG CCT TAG
		R	AGC CAG GTT GAT GAT GAG CAC ATT GAA
Probable G-protein coupled receptor 112	GPR112	F	ATT GCC ACT GCT GCG ACA CTG C
		R	CCC ATC CAG GTG AAA GAA GCC AGT AGG
