# Supplementary material for: Gene Expression Profiling Reveals Functional Specialization along the Intestinal Tract of a Carnivorous Teleostean Fish (Dicentrarchus labrax)
Source: Front Physiol. 2016 Aug 25;7:359. doi: 10.3389/fphys.2016.00359 (PMC4997091; doi:10.3389/fphys.2016.00359)
Supplement: Supplementary file 2 [file Table2.PDF]

TABLE S2. Differentially expressed genes between AI-MI and PI.

Clone	Description	Fold-change (AI-MI/PI)
L12_76973	*1,2-dihydroxy-3-keto-5-methylthiopentene dioxygenase*	-2.72
L1_53088	*14 kDa apolipoprotein*	10790.68
L12_73933	*17-beta-hydroxysteroid dehydrogenase 14*	-11.25
L2_34731	*17-beta-hydroxysteroid dehydrogenase 14-like*	-9.80
L12_83613	*1-acylglycerol-3-phosphate O-acyltransferase ABHD5*	1.76
L12_79134	*1-acyl-sn-glycerol-3-phosphate acyltransferase alpha*	5.00
L3_80221	*1-acyl-sn-glycerol-3-phosphate acyltransferase epsilon*	2.09
L1_44524	*1-phosphatidylinositol 3-phosphate 5-kinase*	-2.66
L2_57624	*1-phosphatidylinositol 4,5-bisphosphate phosphodiesterase beta-1*	-1.52
L2_67868	*1-phosphatidylinositol 4,5-bisphosphate phosphodiesterase beta-4*	-2.41
L2_57426	*1-phosphatidylinositol 4,5-bisphosphate phosphodiesterase delta-3-A*	-1.68
L1_66165	*1-phosphatidylinositol 4,5-bisphosphate phosphodiesterase epsilon-1*	3.39
L1_28014	*1-phosphatidylinositol 4,5-bisphosphate phosphodiesterase epsilon-1-like*	2.35
L2_65369	*1-phosphatidylinositol 4,5-bisphosphate phosphodiesterase eta-1*	-3.89
L12_90114	*1-phosphatidylinositol 4,5-bisphosphate phosphodiesterase gamma-2*	-1.68
L12_69208	*1-phosphatidylinositol phosphodiesterase-like*	3.37
L1_51447	*2,5-diketo-D-gluconic acid reductase A*	1.98
L3_42818	*28S ribosomal protein S21, mitochondrial*	1.52
L12_82494	*2-acylglycerol O-acyltransferase 2-A*	3.02
L3_30720	*2-amino-3-carboxymuconate-6-semialdehyde decarboxylase*	2.51
L3_51580	*2-amino-3-ketobutyrate coenzyme A ligase, mitochondrial*	1.57
L2_39720	*2-hydroxypropyl-CoM lyase*	1.79
L12_89407	*2-oxoglutarate dehydrogenase, mitochondrial*	13.44
L12_83295	*3 beta-hydroxysteroid dehydrogenase type 7*	3.72
L12_80399	*3'(2'),5'-bisphosphate nucleotidase 1*	1.88
L3_48877	*39S ribosomal protein L14, mitochondrial*	-2.01
L1_30556	*39S ribosomal protein L42, mitochondrial*	-1.94
L12_43599	*39S ribosomal protein L52, mitochondrial*	1.97
L12_76005	*3-hydroxyanthranilate 3,4-dioxygenase*	1.73
L3_65893	*3-hydroxybutyrate dehydrogenase type 2*	1.67
L1_65887	*3-hydroxypropionyl-coenzyme A dehydratase-like*	2.00
L12_85438	*3-ketoacyl-CoA thiolase, mitochondrial*	4.07
L1_76175	*3-keto-steroid reductase*	2.40
L12_69409	*3-mercaptopyruvate sulfurtransferase*	8.82

L12_75313	*3-oxo-5-beta-steroid 4-dehydrogenase*	-1.61
L1_52800	*3-oxoacyl- synthase 2*	1.83
L12_83536	*40S ribosomal protein S9*	-2.51
L12_74177	*4-aminobutyrate aminotransferase, mitochondrial*	1.51
L1_57850	*4F2 cell-surface antigen heavy chain-like*	-1.55
L12_88485	*5'(3')-deoxyribonucleotidase, mitochondrial*	2.04
L1_17264	*50S ribosomal protein L14*	3.08
L12_89796	*55 kDa erythrocyte membrane protein*	1.97
L12_87399	*5-aminolevulinate synthase, nonspecific, mitochondrial*	-1.87
L12_82019	*5'-AMP-activated protein kinase subunit gamma-1*	-2.84
L2_49683	*5-hydroxyisourate hydrolase*	6.03
L2_15712	*5-hydroxytryptamine receptor 4*	-1.72
L2_44746	*5-hydroxytryptamine receptor 7*	-2.13
L12_77102	*5'-nucleotidase*	-1.95
L1_67485	*5-oxoprolinase*	1.52
L12_86581	*60S ribosomal export protein NMD3*	1.87
L1_24530	*6-methylsalicylic acid synthase*	3.00
L12_83586	*6-phosphofructo-2-kinase/fructose-2,6-bisphosphatase 1*	1.81
L12_86717	*6-phosphofructo-2-kinase/fructose-2,6-bisphosphatase 4*	2.69
L12_74324	*7,8-dihydro-8-oxoguanine triphosphatase*	-1.58
L12_88372	*7-alpha-hydroxycholest-4-en-3-one 12-alpha-hydroxylase*	23.44
L12_72066	*7-dehydrocholesterol reductase*	3.61
L2_60426	*A disintegrin and metalloproteinase with thrombospondin motifs 15*	-1.83
L1_22765	*A disintegrin and metalloproteinase with thrombospondin motifs 8*	-2.44
L2_44479	*ABC transporter, ATP-binding & transmembrane domain*	1.58
L1_2432	*Abhydrolase 3*	-2.70
L1_29490	*Abhydrolase domain-containing protein 1*	1.99
L12_73538	*Abhydrolase domain-containing protein 2*	2.55
L2_10164	*Abhydrolase domain-containing protein 2-A*	-2.15
L1_55149	*Abhydrolase domain-containing protein 2-B*	-2.53
L2_42027	*Abhydrolase domain-containing protein 3-like*	1.60
L1_57208	*Abhydrolase domain-containing protein 4*	2.19
L2_38969	*Abhydrolase domain-containing protein FAM108A1*	-1.61
L12_77324	*Abnormal spindle-like microcephaly-associated protein homolog*	-1.55
L12_81263	*Absent in melanoma 1 protein*	-1.58
L1_60097	*Absent in melanoma 1-like protein*	-2.05
L12_82787	*Acetyl-CoA acetyltransferase, mitochondrial*	-1.60
L3_80818	*Acetyl-CoA carboxylase*	2.66
L2_57044	*Acetyl-CoA carboxylase 2*	2.43
L12_73999	*Acetyl-coenzyme A synthetase 2-like, mitochondrial*	-3.02
L1_33761	*Achaete-scute homolog 1a*	2.71
L12_85085	*Acid ceramidase*	-2.13

L12_89653	*Acid sphingomyelinase-like phosphodiesterase 3b*	1.83
L12_81287	*Acid trehalase-like protein 1*	-3.26
L1_86990	*Acidic mammalian chitinase*	24297.52
L2_39622	*Actin, adductor muscle*	1.69
L12_84053	*Actin, cytoplasmic 2*	-1.77
L2_56930	*Actin-binding Rho-activating protein*	-3.09
L12_87983	*Actin-related protein 2/3 complex subunit 1A*	3.41
L12_47586	*Actin-related protein 2/3 complex subunit 5*	-1.61
L2_58427	*Actin-related protein 3B*	-3.69
L12_63438	*Activation-induced cytidine deaminase*	-2.29
L12_84307	*Activator of 90 kDa heat shock protein ATPase homolog 1*	3.33
L12_82044	*Activin receptor type-1*	-1.69
L12_79868	*Activin receptor type-2A*	1.83
L12_39178	*Acyl-CoA dehydrogenase family member 11*	2.07
L1_17140	*Acyl-CoA-binding domain-containing protein 5*	1.84
L3_37184	*Acyl-CoA-binding protein*	1.53
L3_33118	*Acyl-CoA-binding protein homolog*	2.89
L12_88614	*Acyl-coenzyme A thioesterase 11*	2.70
L3_45558	*Acyl-coenzyme A thioesterase 11-like*	2.68
L3_44774	*Acyl-coenzyme A thioesterase 13*	-1.67
L3_83960	*Acyl-coenzyme A thioesterase 2, mitochondrial*	1.58
L12_81603	*Acyl-coenzyme A thioesterase 4*	-2.67
L3_79381	*Acylphosphatase-2*	-2.28
L12_74101	*Acyl-protein thioesterase 2*	-1.58
L1_56864	*ADAM metallopeptidase with thrombospondin type 1 motif, 20*	1.89
L2_46043	*ADAMTS-like protein 1*	-1.55
L2_48496	*ADAMTS-like protein 5-like*	-1.83
L2_57639	*Adapter protein CIKS*	-1.54
L12_77433	*Adenosine kinase*	-1.77
L1_36412	*Adenosine receptor A1*	1.81
L3_79115	*Adenosylhomocysteinase*	2.28
L12_88337	*Adenosylhomocysteinase A*	3.06
L2_63776	*Adenylate cyclase type 6*	1.58
L2_49679	*Adenylate cyclase type 9*	1.95
L2_21858	*Adenylate kinase 7*	-3.18
L12_80450	*Adenylate kinase isoenzyme 1*	-1.90
L3_56388	*Adenylate kinase isoenzyme 4, mitochondrial*	-1.64
L12_83380	*Adenylosuccinate lyase*	1.73
L12_82273	*Adenylosuccinate synthetase isozyme 1 C*	-5.38
L12_81789	*Adenylyl cyclase-associated protein 2*	-2.90
L1_63136	*Adiponectin receptor protein 1*	1.77
L12_84187	*Adiponectin receptor protein 2*	6.82
L1_33733	*ADP/ATP translocase 3*	2.02
L3_61110	*ADP-ribosylation factor 4*	1.60

L12_87165	*ADP-ribosylation factor GTPase-activating protein 3*	1.67
L3_75571	*ADP-ribosylation factor-like protein 16*	14.92
L12_83557	*ADP-ribosylation factor-like protein 4C*	-2.62
L2_21812	*ADP-ribosylation factor-like protein 4D*	-1.55
L12_85402	*ADP-ribosylation factor-like protein 5A*	2.02
L3_62283	*ADP-ribosylation factor-like protein 8B*	-1.68
L3_59832	*ADP-ribosylation factor-like protein 8B-A*	-3.38
L12_79160	*ADP-sugar pyrophosphatase*	-1.51
L12_82910	*Adrenodoxin, mitochondrial*	2.87
L12_82647	*Adrenomedullin*	-5.20
L2_52612	*Adseverin*	27.49
L1_33391	*Adseverin-like*	3.97
L12_89223	*Advillin*	2.44
L12_84486	*AF4/FMR2 family member 1*	2.07
L2_69480	*AF4/FMR2 family member 3*	-2.48
L12_88862	*Aflatoxin B1 aldehyde reductase member 2*	1.97
L2_53727	*Agouti-signaling protein-like*	-6.20
L1_35299	*Agrin*	1.86
L1_28160	*A-kinase anchor protein 11-like*	1.84
L12_84550	*A-kinase anchor protein 9*	-1.64
L12_85972	*Alanine aminotransferase 2-like*	-1.60
L12_88750	*Alanine-glyoxylate aminotransferase 2, mitochondrial*	5.91
L12_80932	*Alcohol dehydrogenase A*	-37.24
L12_76681	*Alcohol dehydrogenase B*	-1.77
L12_78166	*Alcohol dehydrogenase class-3*	1.79
L12_76182	*Alcohol dehydrogenase class-3 chain L*	2.34
L12_88865	*Aldehyde dehydrogenase family 16 member A1*	-2.13
L1_64116	*Aldehyde dehydrogenase family 8 member A1*	1.78
L12_85131	*Aldehyde dehydrogenase family 9 member A1*	2.25
L12_85928	*Aldehyde dehydrogenase family 9 member A1-B*	-1.88
L1_47966	*Aldehyde oxidase*	2.11
L12_79109	*Aldose reductase*	1.56
L12_83987	*Alkaline ceramidase 2*	1.88
L2_51465	*Alkaline ceramidase 3*	3.15
L1_77244	*Alkaline phosphatase, tissue-nonspecific isozyme*	407.53
L12_85509	*Alkylglycerol monooxygenase*	2.94
L1_35303	*Alkylglycerol monooxygenase-like*	2.20
L3_30163	*Allantoicase*	4.19
L12_65705	*Allograft inflammatory factor 1-like*	-1.53
L12_83439	*All-trans retinoic acid-induced differentiation factor*	-4.43
L2_46946	*Alpha adaptinC2*	-2.98
L3_26321	*Alpha-(1,3)-fucosyltransferase*	1.95
L2_36697	*Alpha-(1,6)-fucosyltransferase*	-1.68
L2_26946	*Alpha/beta hydrolase domain-containing protein 11*	1.83
L1_66312	*Alpha-1,2-mannosyltransferase ALG9*	1.86

L2_34251	*Alpha-1,3-mannosyl-glycoprotein 4-beta-N-acetylglucosaminyltransferase A*	1.84
L12_87529	*Alpha-1,3-mannosyl-glycoprotein 4-beta-N-acetylglucosaminyltransferase B*	2.24
L1_22299	*Alpha-1,3-mannosyl-glycoprotein 4-beta-N-acetylglucosaminyltransferase C*	2.80
L3_58034	*Alpha-1-antitrypsin*	2.03
L2_44185	*Alpha-2 adrenergic receptor*	-10.17
L3_77618	*Alpha-2,8-sialyltransferase 8E*	-3.75
L12_65403	*Alpha-2,8-sialyltransferase 8F*	-2.89
L1_4859	*Alpha-2A adrenergic receptor*	2.57
L1_58066	*Alpha-2B adrenergic receptor*	1.67
L1_17670	*Alpha-2C adrenergic receptor*	1.79
L3_71324	*Alpha-2-macroglobulin*	1.61
L12_74564	*Alpha-2-macroglobulin receptor-associated protein*	-9.98
L3_60644	*Alpha-2-macroglobulin-like*	-1.95
L1_43747	*Alpha-2-macroglobulin-like protein 1*	-1.82
L1_24657	*Alpha-aminoadipic semialdehyde synthase, mitochondrial*	2.66
L12_72154	*Alpha-aspartyl dipeptidase*	1.84
L12_85792	*Alpha-galactosidase A*	-6.27
L2_58838	*Alpha-mannosidase 2C1*	-1.75
L1_27409	*Alpha-methylacyl-CoA racemase*	1.81
L2_71330	*Alpha-N-acetylgalactosaminide alpha-2,6-sialyltransferase 2*	-3.39
L12_89554	*Alpha-N-acetylglucosaminidase*	-4.12
L12_79374	*Alpha-protein kinase 1*	-1.55
L12_89668	*Amiloride-sensitive amine oxidase*	115.01
L12_86919	*Aminoacyl tRNA synthase complex-interacting multifunctional protein 2*	1.72
L1_19420	*Aminomethyltransferase, mitochondrial*	3.67
L12_27763	*Aminopeptidase N-like*	1.90
L1_84283	*Ammonium transporter Rh type B*	359.73
L12_86567	*AMP deaminase 2*	1.51
L1_39604	*Amphiphysin*	-1.58
L2_51999	*Amyloid beta A4 protein-binding family B member 2*	-1.53
L1_6266	*Amyloid-like protein 2*	2.79
L2_44824	*Androgen receptor*	1.82
L2_57325	*Angiopoietin-related protein 1*	-2.31
L12_38156	*Angiopoietin-related protein 2*	2.05
L12_51421	*Angiotensin-converting enzyme*	2.21
L12_90217	*Angiotensin-converting enzyme 2*	5.18
L1_43185	*Anion exchange protein 2*	-3.49
L2_59023	*Ankycorbin*	1.73
L1_40628	*Ankyrin repeat and death domain-containing protein 1B*	1.56
L12_83035	*Ankyrin repeat and FYVE domain-containing protein 1*	-2.18

L12_86978	*Ankyrin repeat and SAM domain-containing protein 4B*	1.54
L3_76576	*Ankyrin repeat and SOCS box protein 16*	2.07
L12_86622	*Ankyrin repeat and SOCS box protein 2*	-1.72
L2_67187	*Ankyrin repeat and SOCS box protein 9*	-2.14
L2_20771	*Ankyrin repeat and sterile alpha motif domain-containing protein 1B*	-1.89
L12_88482	*Ankyrin repeat and zinc finger domain-containing protein 1*	1.74
L1_46075	*Ankyrin repeat domain-containing protein 27*	-1.63
L12_85642	*Ankyrin repeat domain-containing protein SOWAHA*	1.53
L12_82095	*Annexin A2*	7.90
L12_81615	*Annexin A3*	2.34
L1_66349	*Anoctamin*	-2.22
L12_67411	*Anoctamin-10*	-2.77
L12_79712	*Anoctamin-3*	-1.80
L12_78998	*Anoctamin-6*	-1.87
L12_72802	*Anoctamin-6-like*	-1.62
L12_88605	*Anoctamin-9*	2.21
L1_24439	*Anthrax toxin receptor 2*	-1.60
L4_89643	*Antigen peptide transporter 1*	-1.53
L4_86478	*Anti-Mullerian hormone*	1.62
L12_75169	*AP-1 complex subunit sigma-3*	-1.60
L1_42012	*AP-2 complex subunit alpha-2*	-2.31
L1_61801	*AP-2 complex subunit mu*	-1.52
L12_87052	*AP-2 complex subunit sigma*	-1.86
L12_60753	*AP-3 complex subunit delta-1*	-1.93
L12_88867	*AP-3 complex subunit mu-2*	-2.59
L12_81965	*AP-3 complex subunit sigma-1*	-3.23
L12_77439	*AP-4 complex subunit mu-1*	1.57
L12_82759	*AP-4 complex subunit sigma-1*	-7.71
L12_85624	*AP-5 complex subunit mu-1*	-1.89
L2_33507	*APC membrane recruitment protein 1*	2.23
L12_89586	*Apical endosomal glycoprotein*	6.71
L2_29027	*Apical endosomal glycoprotein-like*	14.24
L1_13419	*APOBEC1 complementation factor*	2.36
L1_22120	*Apolipoprotein A-I*	31.66
L12_86775	*Apolipoprotein A-IV*	8.47
L2_47644	*Apolipoprotein A-IV3*	14.81
L3_83747	*Apolipoprotein B*	26.29
L12_89941	*Apolipoprotein B-100*	71.69
L2_28353	*Apolipoprotein B-100-like*	47.85
L1_10210	*Apolipoprotein B-100-like, partial*	24.63
L12_59073	*Apolipoprotein C-II*	908.45
L1_36472	*Apolipoprotein C-I-like*	174.69
L3_81365	*Apolipoprotein Eb*	52.61

L2_8349	*Apoptosis-inducing factor 3-like*	-4.33
L1_36074	*Apoptosis-stimulating of p53 protein 1*	1.67
L1_9175	*Apoptosis-stimulating of p53 protein 2-like*	1.59
L3_58589	*Apoptotic protease-activating factor 1*	1.81
L4_71738	*Aquaporin FA-CHIP*	-2.95
L12_85158	*Aquaporin-8*	572.00
L2_7484	*Arfaptin*	-1.93
L12_76550	*Arf-GAP domain and FG repeat-containing protein 1*	3.47
L2_59607	*Arf-GAP with Rho-GAP domain, ANK repeat and PH domain-containing protein 1-like*	-1.55
L2_50412	*Arf-GAP with SH3 domain, ANK repeat and PH domain-containing protein 3*	-1.51
L1_49433	*Arginase-2, mitochondrial*	32.56
L3_37188	*Arginase-2, mitochondrial-like*	30.33
L12_87226	*Argininosuccinate lyase*	-3.62
L12_83771	*Argininosuccinate synthase*	-1.93
L3_58504	*Arginyl-tRNA-protein transferase 1*	-1.53
L12_72142	*ARL14 effector protein*	-1.60
L1_32601	*Aromatic-L-amino-acid decarboxylase*	10.74
L1_23212	*Arrestin C*	1.66
L2_70151	*Arrestin domain-containing protein 1-like*	1.63
L12_85044	*Arsenite methyltransferase*	6.35
L1_48517	*Arylacetamide deacetylase-like*	-4.20
L2_48102	*Arylacetamide deacetylase-like 4*	-1.53
L1_23817	*Aryl-hydrocarbon-interacting protein-like 1*	14.89
L12_88893	*Arylsulfatase A*	3.03
L1_37200	*Arylsulfatase G*	1.68
L12_83186	*Arylsulfatase K*	-1.88
L12_84746	*Asialoglycoprotein receptor 1*	-1.63
L2_43296	*Asparagine synthetase*	-2.52
L3_77340	*Aspartate aminotransferase, mitochondrial*	1.63
L12_86878	*Aspartoacylase*	1.94
L12_83589	*Aspartoacylase-2B*	-1.59
L1_45642	*Aspartyl aminopeptidase*	3.50
L3_79880	*Asporin*	1.61
L3_62941	*Astrocytic phosphoprotein PEA-15*	-1.51
L1_61406	*Ataxin-1*	2.00
L12_78023	*Atlastin-2*	2.02
L3_31807	*ATP synthase subunit g, mitochondrial*	2.24
L2_63575	*ATP synthase, H+ transporting, mitochondrial F0 complex, subunit d*	2.24
L12_44597	*ATPase inhibitor, mitochondrial*	-1.64
L1_74986	*ATP-binding cassette sub-family A member 1*	20.25
L3_79984	*ATP-binding cassette sub-family A member 12*	2.24
L2_52267	*ATP-binding cassette sub-family A member 3*	-2.28

L12_88609	*ATP-binding cassette sub-family B member 10, mitochondrial*	1.68
L1_58065	*ATP-binding cassette sub-family B member 6, mitochondrial*	3.69
L2_31837	*ATP-binding cassette sub-family D member 3*	1.66
L12_88207	*ATP-binding cassette sub-family D member 4*	-3.84
L12_88559	*ATP-binding cassette sub-family G member 5*	2.08
L3_73426	*ATP-binding cassette sub-family G member 8*	2.83
L2_33232	*ATP-dependent RNA helicase SUPV3L1, mitochondrial*	1.60
L2_39232	*ATP-sensitive inward rectifier potassium channel 10*	-2.71
L2_30430	*AT-rich interactive domain-containing protein 3A*	-4.11
L2_58270	*AT-rich interactive domain-containing protein 3B-like*	3.58
L12_87245	*B- and T-lymphocyte attenuator-like*	-2.09
L2_89061	*B(0,+)-type amino acid transporter 1*	-314.06
L2_27972	*B(0,+)-type amino acid transporter 1-like*	1.88
L12_79957	*B7-H1/DC protein*	-2.43
L2_68858	*B9 domain-containing protein 1*	-1.87
L12_68930	*BAG family molecular chaperone regulator 2*	-1.58
L12_88932	*Band 4.1-like protein 2*	-4.89
L1_43403	*Band 4.1-like protein 2-like*	-1.66
L2_52768	*Band 4.1-like protein 4B*	2.55
L3_76506	*Barrier-to-autointegration factor*	-1.59
L12_75474	*Basic leucine zipper transcriptional factor ATF-like*	-1.81
L2_53639	*Basic leucine zipper transcriptional factor ATF-like 3*	-6.51
L12_82586	*B-cell antigen receptor complex-associated protein alpha chain-like*	-11.53
L3_81205	*B-cell linker protein*	-2.62
L1_70456	*B-cell linker protein-like*	-4.53
L2_47199	*B-cell lymphoma/leukemia 11B*	-2.24
L12_74240	*B-cell receptor CD22*	-4.23
L12_89786	*Bcl2 antagonist of cell death*	2.27
L12_75567	*Bcl-2-related ovarian killer protein homolog A*	-1.60
L2_22067	*BCL-6 corepressor-like, partial*	1.63
L12_85635	*Beta,beta-carotene 15,15'-monooxygenase*	8.67
L12_85268	*Beta-1,3-galactosyl-O-glycosyl-glycoprotein beta-1,6-N-acetylglucosaminyltransferase*	-2.40
L1_16978	*Beta-1,3-galactosyl-O-glycosyl-glycoprotein beta-1,6-N-acetylglucosaminyltransferase 7 '*	1.68
L12_85815	*Beta-1,3-galactosyltransferase 2*	-1.87
L3_85664	*Beta-1,3-N-acetylglucosaminyltransferase lunatic fringe*	-3.11
L12_70640	*Beta-1,4 N-acetylgalactosaminyltransferase 1*	-2.01
L12_89743	*Beta-1,4-galactosyltransferase 5*	1.55
L12_87959	*Beta-1,4-N-acetylgalactosaminyltransferase 3*	1.90
L1_21544	*Beta-1-syntrophin*	-3.14
L1_21520	*Beta-2-glycoprotein 1-like*	5.83

L3_85123	*Beta-catenin-interacting protein 1*	-1.55
L12_73497	*Beta-catenin-like protein 1*	-1.66
L2_43285	*Beta-chimaerin*	2.13
L2_17762	*Beta-defensin*	-157.27
L12_82350	*Beta-enolase*	3.68
L12_88439	*Beta-galactosidase*	-23.58
L12_88598	*Beta-galactosidase-1-like protein 2*	1.95
L12_50449	*Beta-galactoside-binding lectin*	-1.86
L12_88969	*Beta-glucuronidase*	-3.06
L12_90025	*Beta-hexosaminidase subunit alpha*	-3.99
L2_69389	*Beta-hexosaminidase subunit alpha-like*	-5.79
L12_86146	*Beta-hexosaminidase subunit beta*	-1.54
L1_71762	*Beta-ketoacyl synthase*	15.34
L12_85396	*Beta-mannosidase*	-1.64
L12_32968	*Beta-microseminoprotein-like*	2.58
L1_18984	*Beta-N-acetylhexosaminidase*	2.21
L12_73257	*Beta-parvin*	-3.08
L2_16314	*Bicaudal D-related protein 1-like*	-1.97
L3_80099	*Bifunctional 3'-phosphoadenosine 5'-phosphosulfate synthase 1*	-2.47
L12_87490	*Bifunctional 3'-phosphoadenosine 5'-phosphosulfate synthase 2*	-1.65
L12_84141	*Bifunctional coenzyme A synthase*	-1.88
L2_29353	*Bifunctional folylpolyglutamate synthase/ dihydrofolate synthase*	2.30
L12_87171	*Bifunctional purine biosynthesis protein PURH*	-1.94
L12_86747	*Bifunctional UDP-N-acetylglucosamine 2-epimerase/N-acetylmannosamine kinase*	1.80
L12_89171	*Bile acid-CoA:amino acid N-acyltransferase*	-2.63
L1_72488	*Bile salt export pump*	62.99
L12_84525	*Bile salt-activated lipase*	194.54
L1_34057	*Binding-protein-dependent transport systems inner membrane component*	2.61
L3_79062	*Biogenesis of lysosome-related organelles complex 1 subunit 4*	-2.10
L12_68439	*Biogenesis of lysosome-related organelles complex 1 subunit 6*	-3.50
L2_47678	*Bloodthirsty-2*	-1.87
L2_50588	*BMP-2-inducible protein kinase*	-2.07
L2_17545	*BOC cell adhesion associated, oncogene regulated*	-1.73
L12_85458	*Bone morphogenetic protein 2*	2.94
L12_83800	*Brain protein I3*	-1.75
L4_53521	*Brain-derived neurotrophic factor*	-2.84
L2_67837	*Brain-specific angiogenesis inhibitor 1-associated protein 2-like protein 1-like*	1.76

L12_58884	*Brain-specific angiogenesis inhibitor 1-associated protein 2-like protein 2*	1.94
L2_15378	*Branched-chain-amino-acid aminotransferase, cytosolic*	-1.88
L1_50334	*BRCA1-associated RING domain protein 1*	1.51
L2_46495	*Breakpoint cluster region protein*	1.56
L2_24764	*Breast cancer metastasis-suppressor 1-like protein-A*	-1.53
L2_44292	*Brefeldin A-inhibited guanine nucleotide-exchange protein 3*	-2.35
L3_75218	*Brevican core protein*	7.17
L12_77572	*Bromodomain adjacent to zinc finger domain protein 2B*	-1.56
L1_29737	*Bromodomain-containing protein 7-like*	-1.53
L1_58205	*Brorin-like*	-2.40
L1_18528	*BTB/POZ domain-containing protein 6-B*	1.79
L3_77971	*BTB/POZ domain-containing protein 8*	1.79
L12_76809	*BTB/POZ domain-containing protein KCTD12*	-1.86
L12_76129	*BTB/POZ domain-containing protein KCTD14*	-1.69
L1_37283	*BTB/POZ domain-containing protein KCTD16*	2.62
L12_83323	*Butyrophilin subfamily 1 member A1-like*	-8.65
L1_37782	*C10orf107*	1.63
L1_65322	*C1q-like protein*	5.89
L2_44255	*C2 calcium-dependent domain-containing protein 4C*	1.96
L12_38938	*C2 calcium-dependent domain-containing protein 4C-like*	2.93
L12_69782	*C2 domain-containing protein 2*	-3.26
L12_85043	*C3a anaphylatoxin chemotactic receptor*	-1.72
L2_743	*C3a anaphylatoxin chemotactic receptor-like*	2.33
L12_72226	*CAAX prenyl protease 1 homolog*	-3.11
L12_88451	*Cadherin EGF LAG seven-pass G-type receptor 1*	1.68
L12_87911	*Cadherin-1*	1.89
L2_39997	*Cadherin-15-like*	1.78
L12_89254	*Cadherin-17*	1.71
L2_69819	*Cadherin-7*	-3.56
L12_90198	*Cadherin-related family member 2*	2.54
L12_84664	*Cadherin-related family member 5*	2.87
L12_82034	*Calcineurin B homologous protein 3*	-2.10
L12_44777	*Calcineurin subunit B type 1*	-2.01
L1_65391	*Calcium uniporter protein, mitochondrial*	-2.14
L1_54723	*Calcium/calmodulin-dependent protein kinase type 1G*	3.29
L12_81326	*Calcium/calmodulin-dependent protein kinase type II delta 1 chain*	1.98
L12_84623	*Calcium/calmodulin-dependent protein kinase type II delta 2 chain*	-4.82
L12_71508	*Calcium-activated potassium channel subunit alpha-1*	2.58
L2_56218	*Calcium-binding mitochondrial carrier protein Aralar1*	-2.48
L12_86753	*Calcium-binding mitochondrial carrier protein SCaMC-1*	2.09
L12_86045	*Calcium-binding mitochondrial carrier protein SCaMC-2*	19.41

L3_79597	*Calcyphosin-like protein*	1.70
L12_86897	*Caldesmon, smooth muscle*	-2.05
L2_81032	*Calmin*	-3.68
L12_84800	*Calmodulin-like protein 4*	2.15
L1_62963	*Calmodulin-regulated spectrin-associated protein 1-B*	1.97
L1_37659	*Calpain-15*	1.62
L2_63215	*Calpain-5*	-4.76
L12_73397	*Calpain-8*	-5.56
L1_15180	*Calpain-9*	13.92
L12_79735	*Calponin-1*	-2.51
L12_84944	*Calponin-3*	-1.81
L1_59005	*Calsyntenin-3*	1.61
L12_82018	*Calumenin-A*	-2.01
L12_79754	*CAMP-dependent protein kinase type II-alpha regulatory subunit*	1.64
L12_72716	*CAMP-regulated phosphoprotein 21*	-1.81
L12_52812	*CAMP-specific 3',5'-cyclic phosphodiesterase 4B*	-2.01
L12_51591	*CAMP-specific 3',5'-cyclic phosphodiesterase 4B-like*	-2.05
L2_52535	*CAMP-specific 3',5'-cyclic phosphodiesterase 7B*	-1.82
L12_89664	*Canalicular multispecific organic anion transporter 1*	-1.63
L12_78640	*Cannabinoid receptor 2-like*	-2.17
L2_40582	*Cannabinoid receptor type 1A*	-2.47
L2_25730	*Capping protein (Actin filament) muscle Z-line, alpha 2*	-1.64
L1_43049	*Carbamoyl-phosphate synthase large chain*	1.66
L1_67167	*Carbohydrate sulfotransferase 1*	2.06
L12_79081	*Carbohydrate sulfotransferase 12*	-1.93
L12_86847	*Carbohydrate sulfotransferase 15*	-1.90
L2_52344	*Carbohydrate sulfotransferase 3*	-1.73
L1_46990	*Carbohydrate-responsive element-binding protein*	2.40
L12_87679	*Carbonic anhydrase 1*	38.90
L12_85270	*Carbonic anhydrase 4*	218.15
L2_77736	*Carbonic anhydrase 5B, mitochondrial*	-4.29
L12_89283	*Carbonic anhydrase 7*	3.11
L12_82526	*Carbonyl reductase 1*	1.90
L1_58064	*Carboxyl-terminal PDZ ligand of neuronal nitric oxide synthase protein*	2.25
L12_75680	*Carboxymethylenebutenolidase homolog*	-2.71
L12_79757	*Carboxypeptidase A1*	7.84
L12_88460	*Carboxypeptidase A2*	99.28
L12_81698	*Carboxypeptidase B*	36.45
L2_61148	*Carboxypeptidase D*	-1.68
L12_87778	*Carboxypeptidase O*	1.59
L12_88046	*Carboxypeptidase Q*	-2.87
L12_81076	*Carboxypeptidase Z*	-2.51

L12_90005	*Carboxy-terminal domain RNA polymerase II polypeptide A small phosphatase 1*	-1.77
L1_86196	*Carcinoembryonic antigen-related cell adhesion molecule 5*	55.83
L2_58537	*Carcinoembryonic antigen-related cell adhesion molecule 5-like*	-6.83
L1_40940	*Cardiolipin synthase*	1.64
L12_84739	*Carnitine O-palmitoyltransferase 1, liver isoform*	-3.26
L3_48229	*Cartilage matrix protein*	1.90
L12_82857	*Cartilage-associated protein*	-3.13
L1_62583	*CAS1 domain-containing protein 1*	1.98
L12_85281	*CASP8 and FADD-like apoptosis regulator*	-1.53
L12_83535	*Caspase recruitment domain-containing protein 11*	-1.64
L1_51933	*Caspase recruitment domain-containing protein 14*	1.90
L4_52987	*Caspase-1 protein*	-1.98
L12_88365	*Caspase-6*	-2.24
L12_88420	*Caspase-8*	-1.71
L12_53524	*Cat eye syndrome critical region protein 5 homolog*	2.20
L12_87749	*Catalase*	2.60
L12_70240	*Catechol O-methyltransferase*	-1.57
L12_71931	*Catechol O-methyltransferase domain-containing protein 1*	-1.74
L12_37418	*Catenin beta*	-1.57
L12_80325	*Cathepsin K*	-1.75
L2_51754	*Cathepsin L-like*	-367.05
L1_52723	*Cation efflux system protein CusA*	1.71
L1_40884	*Cation transport regulator-like protein 2*	1.95
L3_73213	*Cation-dependent mannose-6-phosphate receptor*	-1.84
L12_74776	*Cation-independent mannose-6-phosphate receptor*	-1.89
L3_13519	*Caveolin-1*	-1.97
L12_68039	*Caveolin-2*	-1.54
L2_22876	*CC chemokine*	-3.36
L4_40862	*CC chemokine 2*	-2.91
L4_68991	*CC chemokine ligand 4*	-2.63
L12_81931	*C-C chemokine receptor type 1*	-1.65
L12_90140	*C-C chemokine receptor type 3*	-1.54
L1_7412	*C-C chemokine receptor type 3-like*	-1.50
L12_87246	*C-C chemokine receptor type 6*	-1.73
L12_78330	*C-C chemokine receptor type 9*	-1.88
L12_76320	*C-C motif chemokine 20-like*	-1.71
L12_85595	*CD151 antigen*	-1.70
L2_50139	*CD166 antigen homolog*	-1.64
L2_69255	*CD209 antigen*	1.67
L12_83298	*CD22*	-3.90
L12_85608	*CD226 antigen*	-1.50

L12_71511	*CD2-associated protein*	-1.89
L2_62300	*CD2-associated protein-like*	-1.97
L4_71171	*CD3 gamma/delta protein*	-2.16
L12_75312	*CD48 antigen-like*	-1.80
L12_75251	*CD59 glycoprotein*	-1.55
L12_57277	*CD59 glycoprotein-like*	-1.97
L12_72056	*CD79b*	-11.52
L12_88071	*CD81 protein*	3.67
L12_78669	*CD83*	-1.82
L4_54031	*CD83 antigen*	-2.80
L12_75184	*CD9 antigen*	-16.38
L12_68928	*Cdc42 effector protein 3*	-2.06
L12_85974	*Cdc42 effector protein 3-like*	-1.89
L12_86650	*Cdc42 effector protein 5*	1.99
L2_19386	*CDK5 and ABL1 enzyme substrate 2*	-4.25
L12_89872	*CDP-diacylglycerol-serine O-phosphatidyltransferase-like*	1.92
L1_33486	*Cell adhesion molecule-related/down-regulated by oncogenes-like*	-1.81
L12_42487	*Cell cycle checkpoint control protein RAD9A-like*	2.32
L3_75481	*Cell cycle checkpoint control protein RAD9B*	-2.67
L12_79022	*Cell death activator CIDE-3*	7.41
L12_81081	*Cell death activator CIDE-B*	4.37
L12_85091	*Cell growth regulator with EF hand domain protein 1*	2.76
L12_77103	*Cellular retinoic acid-binding protein 1*	-2.99
L3_77701	*Cellular retinoic acid-binding protein 2*	-3.47
L2_46063	*Centromere-associated protein E*	1.68
L2_15468	*Centrosomal protein of 152 kDa-like*	-2.04
L1_50476	*Centrosomal protein of 85 kDa*	-1.51
L12_75966	*Centrosomal protein of 97 kDa*	1.61
L1_32694	*Centrosome-associated protein 350*	-1.70
L12_90248	*Ceramide synthase 1*	3.21
L3_81619	*Ceramide synthase 4*	9.69
L12_85147	*Ceramide synthase 5*	3.75
L12_88783	*Ceroid-lipofuscinosis neuronal protein 5*	-2.71
L3_65783	*Ceruloplasmin*	17.29
L12_67071	*CGMP-dependent protein kinase 2-like*	1.59
L1_58441	*CGMP-inhibited 3',5'-cyclic phosphodiesterase B*	1.69
L12_89818	*Chaperone activity of bc1 complex-like, mitochondrial*	1.97
L1_60353	*Chaperone protein DnaK*	1.69
L12_90193	*Charged multivesicular body protein 4b*	-1.93
L12_50258	*Charged multivesicular body protein 6*	-1.69
L12_58513	*Chemokine CC-like protein*	-2.88
L12_90171	*Chloride anion exchanger*	-11.84
L1_30733	*Chloride anion exchanger-like*	-3.68
L12_74660	*Chloride channel protein 2*	1.83

L1_50447	*Chloride intracellular channel protein 6*	4.09
L12_73874	*Cholesterol 24-hydroxylase-like*	2.31
L12_87487	*Choline kinase alpha*	8.33
L1_39172	*Choline transporter-like protein 2*	-2.43
L2_18257	*Choline transporter-like protein 2-like, partial*	-2.69
L12_89440	*Choline transporter-like protein 4*	-4.87
L2_24878	*Choline transporter-like protein 5*	-2.49
L2_27962	*Choline transporter-like protein 5-B*	4.29
L2_66202	*Choline/ethanolaminephosphotransferase 1*	-1.50
L12_75419	*Choline-phosphate cytidylyltransferase B*	8.87
L2_26551	*Cholinephosphotransferase 1*	2.44
L12_89039	*Cholinesterase*	3.00
L12_82183	*Chondroitin sulfate glucuronyltransferase*	-1.57
L3_76335	*Chondroitin sulfate N-acetylgalactosaminyltransferase 2*	1.56
L12_85434	*Chromobox protein homolog 3*	-2.58
L12_83855	*Chromosome 1 SCAF14609, whole genome shotgun sequence*	-1.63
L12_81910	*Chromosome 1 SCAF14995, whole genome shotgun sequence*	4.68
L3_8980	*Chromosome 1 SCAF14998, whole genome shotgun sequence*	-3.13
L2_58841	*Chromosome 1 SCAF15008, whole genome shotgun sequence.*	-1.63
L12_88716	*Chromosome 10 SCAF15019, whole genome shotgun sequence*	3.96
L3_76276	*Chromosome 10 SCAF15019, whole genome shotgun sequence.*	1.62
L1_57107	*Chromosome 10 SCAF15123, whole genome shotgun sequence*	1.59
L2_37634	*Chromosome 11 SCAF14979, whole genome shotgun sequence.*	1.73
L2_45310	*Chromosome 12 SCAF14652, whole genome shotgun sequence.*	-2.73
L1_66656	*Chromosome 12 SCAF14692, whole genome shotgun sequence.*	2.26
L2_36081	*Chromosome 12 SCAF14993, whole genome shotgun sequence*	-1.62
L1_43519	*Chromosome 12 SCAF14999, whole genome shotgun sequence*	1.73
L1_63179	*Chromosome 12 SCAF15104, whole genome shotgun sequence*	1.53
L2_58850	*Chromosome 13 SCAF14715, whole genome shotgun sequence.*	-2.53
L12_72121	*Chromosome 13 SCAF15035, whole genome shotgun sequence*	2.31

L1_67054	*Chromosome 15 SCAF14542, whole genome shotgun sequence*	-10.52
L2_51045	*Chromosome 16 SCAF15002, whole genome shotgun sequence*	6.87
L12_84314	*Chromosome 16 SCAF15002, whole genome shotgun sequence.*	-1.74
L1_49697	*Chromosome 18 SCAF15100, whole genome shotgun sequence*	-1.62
L2_68612	*Chromosome 19 SCAF14664, whole genome shotgun sequence.*	-2.34
L12_82412	*Chromosome 20 SCAF14744, whole genome shotgun sequence*	2.71
L12_88175	*Chromosome 21 SCAF15029, whole genome shotgun sequence*	-3.06
L2_13571	*Chromosome 3 SCAF14700, whole genome shotgun sequence*	-3.88
L12_74730	*Chromosome 3 SCAF14707, whole genome shotgun sequence*	-1.84
L2_45956	*Chromosome 3 SCAF14707, whole genome shotgun sequence*	-2.47
L2_21937	*Chromosome 4 SCAF14533, whole genome shotgun sequence.*	3.87
L1_57366	*Chromosome 4 SCAF14752, whole genome shotgun sequence*	-2.32
L1_79692	*Chromosome 7 SCAF14536, whole genome shotgun sequence*	112.16
L1_46284	*Chromosome 8 SCAF14543, whole genome shotgun sequence*	-1.53
L12_69911	*Chromosome 8 SCAF15044, whole genome shotgun sequence*	-1.97
L2_63664	*Chromosome 9 SCAF14729, whole genome shotgun sequence*	1.51
L1_49620	*Chromosome undetermined SCAF10112, whole genome shotgun sequence.*	-1.56
L3_57563	*Chromosome undetermined SCAF10300, whole genome shotgun sequence*	57.40
L2_39862	*Chromosome undetermined SCAF11274, whole genome shotgun sequence*	-4.09
L1_17789	*Chromosome undetermined SCAF11462, whole genome shotgun sequence.*	-1.75
L12_76981	*Chromosome undetermined SCAF13694, whole genome shotgun sequence*	-1.79
L3_77883	*Chromosome undetermined SCAF14235, whole genome shotgun sequence*	7.85

L1_49005	*Chromosome undetermined SCAF14653, whole genome shotgun sequence*	-2.12
L12_84101	*Chromosome undetermined SCAF14678, whole genome shotgun sequence*	-2.23
L3_53515	*Chromosome undetermined SCAF14699, whole genome shotgun sequence*	-2.28
L1_47503	*Chromosome undetermined SCAF14724, whole genome shotgun sequence.*	-3.59
L12_74030	*Chromosome undetermined SCAF5070, whole genome shotgun sequence.*	1.67
L2_44188	*Chromosome undetermined SCAF7233, whole genome shotgun sequence.*	-1.74
L12_73913	*Chromosome undetermined SCAF7547, whole genome shotgun sequence*	-8.77
L12_78935	*Chromosome undetermined SCAF7646, whole genome shotgun sequence.*	-1.59
L12_81370	*Chymotrypsin A*	90.76
L12_59715	*Chymotrypsin-C*	180.70
L12_72877	*Chymotrypsin-like elastase family member 2A*	427.20
L12_87016	*Chymotrypsin-like elastase family member 3B*	73.19
L12_71897	*Chymotrypsin-like protease CTRL-1*	22.48
L2_38590	*Cingulin-like protein 1-like*	2.01
L2_45341	*Circadian locomoter output cycles protein kaput*	2.21
L2_8153	*Circadian locomoter output cycles protein kaput-like*	2.12
L2_64937	*Circularly permutated Ras protein 1-like*	-1.75
L12_89830	*Citrate synthase, mitochondrial*	1.92
L12_83582	*C-Jun-amino-terminal kinase-interacting protein 3*	-1.59
L3_81844	*CKLF-like MARVEL transmembrane domain-containing protein 3*	-1.83
L3_55673	*CKLF-like MARVEL transmembrane domain-containing protein 7*	-2.30
L2_51380	*CKLF-like MARVEL transmembrane domain-containing protein 8*	-1.79
L12_78996	*Clarin-3*	1.57
L12_81825	*Class E basic helix-loop-helix protein 41*	-2.81
L3_50869	*Class I helical cytokine receptor number 21*	-2.42
L2_52334	*Class II histocompatibility antigen, B-L beta chain*	-38.13
L12_81231	*Clathrin heavy chain 1*	-1.83
L12_73900	*Clathrin light chain A*	-2.00
L3_76208	*Clathrin light chain B*	1.57
L2_76063	*Claudin-14*	-15.46
L12_86083	*Claudin-2*	51.53
L1_59906	*Claudin-3*	1.93
L12_85053	*Claudin-7-B*	-1.85
L12_88979	*Cleavage stimulation factor subunit 3*	-1.98

L3_16409	*CLIP-associating protein 2*	1.52
L12_87251	*Clusterin*	-1.75
L12_86741	*CMP-N-acetylneuraminate-beta-galactosamide-alpha-2,3-sialyltransferase 1-like*	-9.27
L3_78976	*CMP-N-acetylneuraminate-beta-galactosamide-alpha-2,3-sialyltransferase 4*	2.08
L2_68468	*CMP-N-acetylneuraminate-beta-galactosamide-alpha-2,3-sialyltransferase 4-like*	-4.10
L2_60192	*CMRF35-like molecule 3-like*	-1.72
L2_34551	*C-Myc-binding protein*	-1.65
L1_51595	*Coagulation factor VIII*	-2.52
L1_70125	*Coagulation factor XI*	6.89
L3_71816	*Coagulation factor XIII A chain*	1.78
L1_54507	*Coagulation factor XIII A chain-like*	2.65
L12_73241	*Coagulation factor XI-like*	9.90
L12_88674	*Coatomer subunit gamma-2*	1.51
L1_19001	*Cobalt-zinc-cadmium resistance protein CzcD*	1.63
L12_79854	*Coenzyme Q-binding protein COQ10 homolog, mitochondrial*	-13.68
L2_58277	*Coiled-coil and C2 domain-containing protein 1B*	-1.84
L12_76298	*Coiled-coil domain-containing protein 115*	-2.25
L1_62489	*Coiled-coil domain-containing protein 13*	7.46
L12_87751	*Coiled-coil domain-containing protein 134*	-2.06
L2_45176	*Coiled-coil domain-containing protein 149-B-like*	1.59
L2_29527	*Coiled-coil domain-containing protein 15*	-5.06
L12_87426	*Coiled-coil domain-containing protein 22*	-1.85
L2_66612	*Coiled-coil domain-containing protein 3*	-1.88
L3_75239	*Coiled-coil domain-containing protein 39*	2.21
L1_54161	*Coiled-coil domain-containing protein 48-like*	1.55
L12_82992	*Coiled-coil domain-containing protein 50-like*	-1.77
L12_79860	*Coiled-coil domain-containing protein 74B*	-3.27
L1_36655	*Coiled-coil domain-containing protein 85C-B*	2.47
L1_39210	*Coiled-coil domain-containing protein 88B-like*	-1.53
L12_76369	*Coiled-coil-helix-coiled-coil-helix domain-containing protein 1*	-1.53
L1_14044	*Collagen*	1.68
L12_87215	*Collagen alpha-1(I) chain*	-1.51
L12_74260	*Collagen alpha-1(II) chain*	6.40
L2_68966	*Collagen alpha-1(IV) chain*	2.32
L1_33351	*Collagen alpha-1(XI) chain*	-1.51
L1_13433	*Collagen alpha-1(XVII) chain*	-1.94
L12_86694	*Collagen alpha-1(XVIII) chain*	-5.83
L2_37647	*Collagen alpha-1(XXIV) chain*	-1.79
L12_89873	*Collagen alpha-2(I) chain*	-1.53
L12_86974	*Collagen alpha-2(V) chain*	-1.61

L1_34430	*Collagen triple helix repeat-containing protein 1*	-1.67
L12_88209	*Collagen type IV alpha-3-binding protein*	1.89
L1_39596	*Collectin-11*	1.76
L2_64229	*Collectrin*	-25.35
L3_62856	*COMM domain-containing protein 10*	-1.78
L12_70860	*COMM domain-containing protein 2*	-2.36
L12_65619	*COMM domain-containing protein 3*	-1.68
L12_66049	*COMM domain-containing protein 4*	-3.73
L3_75822	*COMM domain-containing protein 5*	-1.94
L2_47265	*COMM domain-containing protein 8-like*	-2.00
L12_72841	*COMM domain-containing protein 9*	-2.43
L1_40782	*Complement C1q and tumor necrosis factor-related protein 9A*	-2.79
L12_74629	*Complement C1q-like protein 2*	56.64
L12_81038	*Complement C1q-like protein 2-like*	80.12
L12_81462	*Complement C1q-like protein 2-like*	7.68
L3_24652	*Complement C1q-like protein 3-like*	-1.51
L1_51869	*Complement component C8 beta chain*	4.34
L12_88845	*Complement factor B*	12.66
L1_19359	*Complexin-4*	6.64
L1_40710	*Condensin-2 complex subunit D3-like*	1.73
L2_66081	*Connector enhancer of kinase suppressor of ras 3*	-3.22
L2_43866	*Connector enhancer of kinase suppressor of ras 3-like*	-2.99
L12_88546	*Conserved oligomeric Golgi complex subunit 6*	1.52
L12_87153	*COP9 signalosome complex subunit 3*	-2.16
L12_89396	*Copine-3*	-2.64
L12_86497	*Copper homeostasis protein cutC homolog*	1.62
L12_63069	*Copper transport protein ATOX1*	-2.52
L1_25207	*Copper-transporting ATPase 2*	-2.95
L1_26758	*CorA-like Mg2+ transporter family protein*	83.95
L12_74181	*Cordon-bleu protein-like 1*	1.57
L12_84448	*Core-binding factor subunit beta*	-1.71
L3_44493	*Cornifelin*	-1.53
L12_84208	*Coronin-1A*	-1.81
L12_83442	*Coronin-2A*	1.75
L3_77976	*Coronin-6*	-1.96
L1_49406	*Corticotropin-releasing factor receptor 2*	4.94
L12_28519	*Costars family protein ABRACL*	-3.10
L12_86772	*Counting factor associated protein D*	-8.49
L2_35553	*COX8*	1.62
L2_70698	*CR1-3*	-2.17
L3_75027	*C-reactive protein*	-1.67
L1_39505	*C-reactive protein-like*	58.59
L12_82101	*Creatine kinase U-type, mitochondrial*	3.99
L2_58271	*Crystall*	3.10

L1_60184	*CST complex subunit CTC1-like, partial*	1.72
L12_78021	*CST complex subunit TEN1*	-1.69
L12_71974	*CTD small phosphatase-like protein*	-2.07
L3_77630	*C-type lectin domain family 3 member A*	2.52
L4_87554	*C-type lectin domain family 4 member M*	-1.50
L2_63103	*C-type mannose receptor 2-like*	1.69
L12_64878	*CUB domain-containing protein 1-like*	-1.54
L2_88422	*Cubilin*	-513.32
L2_55687	*Cubilin-like*	-1.68
L12_89555	*CUGBP Elav-like family member 2*	-1.61
L1_5319	*C-X-C chemokine receptor type 2*	-1.88
L12_83983	*C-X-C chemokine receptor type 3*	-2.15
L12_84456	*C-X-C chemokine receptor type 4*	-1.71
L12_78022	*C-X-C chemokine receptor type 5*	-11.97
L2_58548	*CXorf33*	1.77
L12_87419	*Cyclic AMP-dependent transcription factor ATF-6 alpha*	-1.53
L12_85435	*Cyclic AMP-responsive element-binding protein 3-like protein 3-A*	2.47
L12_72264	*Cyclic AMP-responsive element-binding protein 3-like protein 3-B*	2.68
L2_58367	*Cyclic nucleotide-gated channel cone photoreceptor subunit alpha*	-1.85
L12_77149	*Cyclin-dependent kinase 6*	-1.52
L2_28094	*Cyclin-F*	-2.35
L3_86634	*Cyclin-G1*	2.06
L2_48774	*Cyclin-J*	-1.51
L12_88481	*Cystathionine beta-synthase*	4.05
L1_51127	*Cystatin-M-like*	-1.78
L3_73849	*Cysteine dioxygenase type 1*	90.56
L12_89158	*Cysteine protease ATG4B*	1.80
L12_85920	*Cysteine protease ATG4C*	-2.15
L1_39734	*Cysteine string protein*	-1.63
L12_69077	*Cysteine string protein-like*	-3.03
L12_89530	*Cysteine/serine-rich nuclear protein 1*	1.62
L2_45175	*Cysteine/serine-rich nuclear protein 3*	1.76
L12_35515	*Cysteine-rich protein 1*	-2.13
L1_64442	*Cysteine-rich with EGF-like domain protein 1*	-1.93
L4_90163	*Cystic fibrosis transmembrane conductance regulator*	1.78
L12_81464	*Cystinosin*	-6.12
L12_83075	*Cytochrome b-245 light chain*	-3.12
L12_77147	*Cytochrome b561*	-2.41
L12_30306	*Cytochrome b-c1 complex subunit 8*	2.60
L3_54025	*Cytochrome c*	1.82
L1_68910	*Cytochrome C biogenesis transmembrane region family protein*	-2.79

L1_25894	*Cytochrome c homolog*	2.10
L1_72746	*Cytochrome c oxidase subunit 5B, mitochondrial*	-2.52
L12_72259	*Cytochrome c oxidase subunit 6B1*	1.91
L3_58499	*Cytochrome c-a*	-1.90
L4_85455	*Cytochrome P450 11B, mitochondrial*	2.30
L4_81935	*Cytochrome P450 1A1*	7.54
L3_76984	*Cytochrome P450 27C1*	1.64
L1_85234	*Cytochrome P450 2D15*	159.04
L1_42385	*Cytochrome P450 2D20*	19.02
L3_37187	*Cytochrome P450 2G1-like*	-2.31
L2_69231	*Cytochrome P450 2J1*	-8.10
L2_62617	*Cytochrome P450 2K1-like*	-1.88
L12_88214	*Cytochrome P450 3A27*	1.68
L1_47674	*Cytochrome P450 4A12A*	-4.65
L12_89669	*Cytochrome P450 4V2*	2.61
L12_87733	*Cytohesin-4*	-1.51
L12_85516	*Cytohesin-interacting protein*	-1.71
L3_79747	*Cytokine receptor common subunit gamma*	-1.87
L12_80547	*Cytokine receptor-like factor 1*	-1.94
L2_7095	*Cytokine receptor-like factor 3*	-1.58
L3_73168	*Cytokine-like protein 1*	1.53
L3_64320	*Cytolysin Src-1-like*	-11.53
L2_56917	*Cytoplasmic dynein 2 heavy chain 1*	2.65
L2_30538	*Cytoplasmic polyadenylation element-binding protein 2*	1.78
L1_41536	*Cytoplasmic polyadenylation element-binding protein 3*	2.17
L12_90047	*Cytosolic 10-formyltetrahydrofolate dehydrogenase*	2.15
L3_82916	*Cytosolic acyl coenzyme A thioester hydrolase*	-2.00
L1_50366	*Cytosolic carboxypeptidase-like protein 5*	-1.68
L1_66038	*Cytosolic Fe-S cluster assembly factor NUBP1*	3.99
L12_83162	*Cytosolic non-specific dipeptidase*	1.94
L2_35251	*D-amino-acid oxidase*	8.06
L12_85870	*D-amino-acid oxidase*	2.73
L2_7997	*Dapper homolog 2*	-13.24
L12_86476	*DAZ-associated protein 1*	1.52
L2_44560	*DDB1- and CUL4-associated factor 4-like*	4.04
L2_44835	*Death inducer-obliterator 1*	-1.52
L3_56124	*Death-associated protein 1*	3.76
L2_44549	*Death-associated protein kinase 1*	-1.82
L12_88440	*Decorin*	-1.64
L1_60025	*Dedicator of cytokinesis protein 10*	-1.71
L12_87813	*Dedicator of cytokinesis protein 2*	-1.91
L12_38710	*Dedicator of cytokinesis protein 8*	-1.68
L2_84481	*Dedicator of cytokinesis protein 9*	-7.37
L12_83088	*Dehydrogenase/reductase SDR family member 1*	5.84
L3_70131	*Dehydrogenase/reductase SDR family member 11*	-1.89

L12_87558	*Dehydrogenase/reductase SDR family member 12*	-2.07
L12_88012	*Deleted in malignant brain tumors 1 protein*	-1.94
L12_88577	*Delta(24)-sterol reductase*	2.13
L12_86853	*Delta-1-pyrroline-5-carboxylate dehydrogenase, mitochondrial*	-2.58
L12_88951	*Delta-aminolevulinic acid dehydratase*	1.83
L12_72405	*Delta-like protein A*	1.58
L1_22980	*Delta-type opioid receptor*	-1.92
L12_89761	*DENN domain-containing protein 1A*	-1.93
L12_89014	*DENN domain-containing protein 3*	-3.91
L2_63299	*DENN domain-containing protein 5B*	4.14
L12_85777	*Deoxycytidylate deaminase*	-1.98
L12_73833	*Deoxyguanosine kinase, mitochondrial*	-2.07
L3_83542	*Deoxyhypusine hydroxylase*	1.53
L1_26520	*Deoxyribonuclease gamma-like*	-5.01
L3_78083	*Deoxyribonuclease-2-alpha*	1.54
L12_78663	*Deoxyribonuclease-2-beta*	-2.32
L2_38887	*Deoxyuridine 5'-triphosphate nucleotidohydrolase*	-1.95
L2_39017	*Deoxyuridine 5'-triphosphate nucleotidohydrolase, mitochondrial*	-1.91
L2_66371	*DEP domain-containing protein 5*	-1.53
L1_45675	*DEP domain-containing protein 7-like*	1.52
L12_89537	*Desmoglein-2*	1.69
L12_90134	*Desmoplakin*	1.59
L12_88998	*Developmentally-regulated GTP-binding protein 1*	1.75
L2_67104	*Diacylglycerol kinase alpha*	4.09
L2_14104	*Diacylglycerol kinase alpha-like*	3.11
L1_61830	*Diacylglycerol kinase delta*	1.95
L2_67098	*Diacylglycerol kinase eta*	2.27
L12_89175	*Diacylglycerol O-acyltransferase 2*	49.84
L2_4845	*Diaphanous GTPase-binding and Diaphanous FH3 and Actin-binding FH2 domain containing protein*	-2.17
L2_53039	*Dickkopf-related protein 2*	-6.79
L12_85597	*Differentially expressed in FDCP 6 homolog*	-2.05
L12_89444	*Differentially expressed in FDCP 8 homolog*	-4.72
L12_22028	*Differentially regulated trout protein*	182.46
L2_12481	*Diguanylate cyclase domain protein*	-1.93
L12_76153	*Dihydrofolate reductase*	-10.26
L12_89592	*Dihydrolipoyllysine-residue succinyltransferase component of 2-oxoglutarate dehydrogenase complex, mitochondrial*	1.66
L12_84454	*Dihydropteridine reductase*	1.62
L2_37989	*Dihydropyrimidinase*	1.97
L12_74951	*Dihydropyrimidinase-related protein 2*	-1.59
L2_33818	*Dihydropyrimidine dehydrogenase*	4.53

L2_46154	*Dimethylglycine dehydrogenase, mitochondrial*	1.53
L12_81156	*Di-N-acetylchitobiase*	-1.72
L12_84773	*Dipeptidase 1*	-2.12
L12_87551	*Dipeptidyl peptidase 1*	-2.16
L12_89717	*Dipeptidyl peptidase 2*	-6.31
L12_30908	*Dipeptidyl peptidase 4*	1.56
L1_50428	*Dipeptidyl-peptidase 2*	-2.89
L1_23554	*Diphosphoinositol polyphosphate phosphohydrolase 2-like*	4.52
L12_89316	*Disabled homolog 2*	-5.15
L2_60841	*Disabled homolog 2-like*	-8.53
L2_63283	*Disco-interacting protein 2 homolog A*	1.64
L2_32633	*Disheveled-associated activator of morphogenesis 1*	1.53
L2_62993	*Disheveled-associated activator of morphogenesis 2*	-1.65
L1_62104	*Disintegrin and metalloproteinase domain-containing protein 19*	-3.18
L12_88366	*Disks large homolog 4*	2.37
L12_89438	*Disrupted in renal carcinoma protein 2*	-2.31
L2_31620	*Disulfide-bond oxidoreductase YghU*	1.73
L1_59472	*Dixin*	-1.73
L2_17977	*DLA class II histocompatibility antigen, DR-1 beta chain-like*	-3.41
L12_89025	*DmX-like protein 1*	-1.82
L12_88808	*DmX-like protein 2*	-2.02
L12_88226	*DNA (cytosine-5)-methyltransferase 3B*	-1.76
L2_28039	*DNA (cytosine-5)-methyltransferase 3B-like*	1.55
L12_84230	*DNA damage-binding protein 2*	-5.70
L12_80669	*DNA damage-inducible transcript 4 protein*	-1.75
L12_46275	*DNA damage-inducible transcript 4-like protein*	-3.81
L12_85684	*DNA damage-regulated autophagy modulator protein 1*	-4.01
L1_31275	*DNA damage-regulated autophagy modulator protein 2*	-3.73
L12_89886	*DNA fragmentation factor subunit beta*	-1.57
L12_83337	*DNA polymerase delta subunit 3*	-2.39
L1_36481	*DNA polymerase theta*	4.83
L12_82653	*DNA replication ATP-dependent helicase/nuclease DNA2*	1.92
L12_82051	*DNA replication complex GINS protein SLD5*	-1.55
L2_62140	*DNA topoisomerase 2-alpha*	-1.60
L1_51874	*DNA topoisomerase 2-beta*	-1.64
L12_39108	*DNA topoisomerase 2-beta-like*	-1.51
L12_66843	*DNA-binding protein Ikaros*	-1.75
L12_44131	*DNA-binding protein inhibitor ID-2*	1.51
L12_75456	*DNA-binding protein inhibitor ID-3-A*	-1.68
L12_82665	*DNA-binding protein RFXANK*	-1.89
L2_26856	*DNA-binding protein SATB2*	-3.94
L12_84845	*DNA-directed RNA polymerase I subunit RPA43*	1.52

L1_49405	*DNA-directed RNA polymerase III subunit RPC3*	1.55
L3_77417	*DNA-directed RNA polymerase III subunit RPC5*	3.68
L12_36956	*DNA-directed RNA polymerase III subunit RPC7*	3.68
L3_76082	*DNA-directed RNA polymerase III subunit RPC8*	1.58
L1_40313	*DNA-directed RNA polymerase subunit alpha*	1.77
L3_30383	*DNA-directed RNA polymerases I, II, and III subunit RPABC5*	1.85
L1_12448	*DnaJ homolog subfamily A member 3, mitochondrial*	1.67
L12_83160	*DnaJ homolog subfamily B member 6*	-1.54
L12_77654	*DnaJ homolog subfamily B member 9*	-1.52
L2_81139	*DnaJ homolog subfamily C member 13*	-3.11
L3_71359	*DnaJ homolog subfamily C member 2*	9.12
L12_86605	*DnaJ homolog subfamily C member 3*	1.89
L3_77696	*Dolichyl-diphosphooligosaccharide-protein glycosyltransferase subunit STT3A*	1.75
L3_78845	*Double-stranded RNA-specific adenosine deaminase*	1.87
L12_77980	*Down syndrome critical region protein 3 homolog*	-1.77
L12_51330	*DPH3 homolog*	2.66
L12_59548	*Drebrin*	2.21
L12_86011	*D-threo-3-hydroxyaspartate dehydratase*	1.55
L12_73001	*DTW domain-containing protein 2*	2.38
L12_84947	*Dual adapter for phosphotyrosine and 3-phosphotyrosine and 3-phosphoinositide*	-2.02
L12_68276	*Dual specificity phosphatase DUPD1*	-3.69
L1_31990	*Dual specificity protein phosphatase 10*	-1.57
L3_76358	*Dual specificity protein phosphatase 12-like*	2.12
L3_78732	*Dual specificity protein phosphatase 2*	-1.97
L2_41094	*Dual specificity protein phosphatase 3*	-1.70
L12_83244	*Dual specificity protein phosphatase 5*	-1.61
L3_13083	*Dual specificity testis-specific protein kinase 1*	-1.69
L1_22823	*Dual specificity testis-specific protein kinase 2*	1.52
L12_79912	*DUF1295 domain protein*	2.02
L12_72278	*Duodenase-1*	-2.03
L12_83055	*Dymeclin*	-1.88
L12_71745	*Dynactin subunit 6*	3.47
L12_81848	*Dynamin*	-1.66
L2_63653	*Dynamin-3*	-1.90
L1_44239	*Dynamin-binding protein*	2.16
L2_57420	*Dynein heavy chain 5, axonemal*	-12.26
L2_52265	*E1-E2 ATPase*	-1.59
L2_62811	*E3 SUMO-protein ligase CBX4*	-1.88
L2_4644	*E3 SUMO-protein ligase CBX4-like*	-1.68
L1_4680	*E3 ubiquitin-protein ligase CBL-like*	2.00
L12_85768	*E3 ubiquitin-protein ligase HECTD3*	1.59
L1_46297	*E3 ubiquitin-protein ligase LNX*	2.37

L2_32330	*E3 ubiquitin-protein ligase MARCH7-like*	2.08
L12_81207	*E3 ubiquitin-protein ligase NEDD4*	-1.71
L3_78759	*E3 ubiquitin-protein ligase NEURL1B*	2.03
L12_86672	*E3 ubiquitin-protein ligase NEURL3*	3.01
L2_64371	*E3 ubiquitin-protein ligase NHLRC1*	-1.56
L1_56799	*E3 ubiquitin-protein ligase PDZRN3*	-1.81
L12_79802	*E3 ubiquitin-protein ligase RNF115*	-4.88
L12_30635	*E3 ubiquitin-protein ligase RNF13*	-3.97
L2_66809	*E3 ubiquitin-protein ligase RNF180*	2.11
L1_70811	*E3 ubiquitin-protein ligase RNF19B*	-1.91
L12_81715	*E3 ubiquitin-protein ligase RNF213*	-1.56
L12_81902	*E3 ubiquitin-protein ligase Rnf220*	1.74
L12_30082	*E3 ubiquitin-protein ligase TRIM71*	1.83
L12_82472	*E3 ubiquitin-protein ligase XIAP*	1.83
L2_30017	*Early growth response protein 3*	-1.94
L1_32230	*Ecto-NOX disulfide-thiol exchanger 1-like*	297.03
L2_36707	*Ectonucleoside triphosphate diphosphohydrolase 1*	-1.72
L12_71934	*Ectonucleoside triphosphate diphosphohydrolase 3*	-1.70
L12_89688	*Ectonucleotide pyrophosphatase/phosphodiesterase family member 3*	6.48
L12_87949	*Ectonucleotide pyrophosphatase/phosphodiesterase family member 6*	2.41
L12_88885	*Ectonucleotide pyrophosphatase/phosphodiesterase family member 7*	328.98
L12_76373	*EF-hand domain-containing family member A2*	1.83
L3_77107	*EF-hand domain-containing protein 1*	2.68
L12_88621	*EGF domain-specific O-linked N-acetylglucosamine transferase*	-2.11
L12_76042	*EGF-like repeat and discoidin I-like domain-containing protein 3*	4.59
L1_22392	*Egl nine homolog 1*	2.30
L3_68266	*Egl nine homolog 3*	3.40
L2_52184	*EH domain-binding protein 1*	-1.54
L12_86238	*EH domain-binding protein 1-like protein 1*	1.52
L12_84246	*EH domain-containing protein 2*	-1.74
L1_23045	*EH domain-containing protein 4*	-1.59
L12_80774	*Elastase-1*	343.84
L1_89155	*Electrogenic sodium bicarbonate cotransporter 1*	65.76
L2_42345	*Electromotor neuron-associated protein 1*	-1.64
L2_42120	*Electron transfer flavoprotein subunit alpha*	1.83
L1_66301	*Electroneutral sodium bicarbonate exchanger 1*	-1.94
L3_80776	*ELMO domain-containing protein 2*	2.30
L12_74371	*Elongation factor 1-alpha*	-2.67
L12_71464	*Elongation factor 1-delta*	-1.97
L12_89218	*Elongation factor 2*	1.91

L1_17576	*ELOVL fatty acid elongase 1*	1.72
L1_63423	*EMILIN-3-like*	3.57
L1_57402	*Emopamil-binding protein-like*	1.85
L12_87269	*Endonuclease domain-containing 1 protein*	61.13
L12_72937	*Endonuclease/exonuclease/phosphatase family domain-containing protein 1*	15.66
L1_48255	*Endophilin-B2*	-2.72
L12_62867	*Endoplasmic reticulum resident protein 27*	49.13
L3_38395	*Endoplasmic reticulum resident protein 27-like*	50.64
L12_82306	*Endothelin-2*	4.43
L12_89070	*Endothelin-converting enzyme 1*	-1.54
L12_88693	*Engulfment and cell motility protein 1*	-1.72
L3_74512	*Enkurin*	1.97
L1_38581	*Enkurin domain-containing protein 1*	2.19
L12_79857	*Enoyl-CoA delta isomerase 2, mitochondrial*	1.84
L1_10363	*Ensconsin*	2.13
L12_89187	*Enteropeptidase*	33.05
L12_88023	*Envoplakin*	5.53
L2_64679	*Envoplakin-like*	-5.58
L2_62431	*Eosinophil peroxidase*	-4.65
L2_70103	*Ephrin type-A receptor 4a*	2.31
L1_65902	*Ephrin type-A receptor 5*	9.83
L1_9795	*Ephrin type-B receptor 1-A*	-1.67
L1_33057	*Ephrin type-B receptor 1-B*	2.89
L2_26663	*Ephrin type-B receptor 4*	1.79
L12_86941	*Ephrin-A1*	3.24
L12_44411	*Ephrin-A5b-like*	2.24
L3_55231	*Epidermal growth factor receptor kinase substrate 8-like protein 1*	8.94
L12_89497	*Epidermal growth factor receptor kinase substrate 8-like protein 2*	2.34
L12_89141	*Epidermal growth factor receptor kinase substrate 8-like protein 3*	1.66
L12_56304	*Epidermal retinol dehydrogenase 2*	2.12
L12_69525	*Epididymal secretory protein E1*	-3.08
L12_86516	*Epididymis-specific alpha-mannosidase*	-4.13
L3_66321	*Epimerase family protein SDR39U1*	2.12
L1_37877	*Epiplakin*	5.00
L2_44176	*Epithelial discoidin domain-containing receptor 1*	-1.70
L12_84521	*Epithelial splicing regulatory protein 2*	2.37
L12_89817	*Epoxide hydrolase 1*	2.15
L1_30259	*Epsin-1*	1.57
L12_76524	*Epsin-3*	1.91
L12_87255	*ER lumen protein retaining receptor 2*	2.41
L1_32542	*ErfK/YbiS/YcfS/YnhG family protein*	1.62

L2_58717	*Espin*	-2.83
L12_89490	*Estradiol 17-beta-dehydrogenase 12-B*	3.34
L4_85646	*Estrogen receptor beta*	3.62
L12_87183	*Ethanolamine-phosphate phospho-lyase*	16.81
L12_87501	*ETS domain-containing protein Elk-4*	-1.83
L2_25847	*ETS translocation variant 4*	-2.93
L3_42070	*Eukaryotic elongation factor 2 kinase*	1.75
L2_6237	*Eukaryotic translation initiation factor 4 gamma 1-like*	1.53
L3_60145	*Eukaryotic translation initiation factor 4E type 3*	-2.52
L1_44895	*Eukaryotic translation initiation factor 5B*	1.81
L2_89236	*Excitatory amino acid transporter 3*	-116.11
L12_79295	*Exocyst complex component 2*	-1.57
L1_55055	*Exocyst complex component 6*	-3.12
L2_51219	*Exocyst complex component 6-like*	-1.52
L2_54902	*Exonuclease 3'-5' domain-containing protein 1*	-7.11
L2_52629	*Exonuclease 3'-5' domain-containing protein 1-like*	-4.65
L12_85263	*Exosome complex component CSL4*	1.74
L3_74163	*Exostosin-1b*	-2.25
L12_72187	*Exostosin-1c*	4.76
L3_54026	*Extended synaptotagmin-1*	-2.24
L2_42124	*Extracellular calcium-sensing receptor*	-27.75
L3_85344	*Extracellular matrix protein 1*	-1.64
L3_78794	*Extracellular sulfatase Sulf-1*	2.81
L12_89457	*Ezrin*	2.12
L3_56407	*Factor B/C2*	6.03
L12_85536	*Factor VIII intron 22 protein*	-1.60
L2_19262	*FAM151B*	-1.72
L1_63535	*Fanconi anemia group E protein-like*	-1.72
L12_39907	*Fatty acid 2-hydroxylase*	-1.53
L4_85688	*Fatty acid desaturase 2*	2.40
L12_84223	*Fatty acid desaturase 6*	2.94
L2_33238	*Fatty acid synthase*	12.41
L12_39581	*Fatty acid-binding protein, intestinal*	16.61
L12_78045	*Fatty acid-binding protein, liver-type*	14.85
L12_84694	*Fatty acyl-CoA hydrolase, medium chain*	2.61
L1_4869	*Fatty acyl-CoA reductase 1*	-2.35
L1_54402	*Fatty aldehyde dehydrogenase*	5.90
L12_89064	*Fatty-acid amide hydrolase 1*	-3.37
L12_81387	*F-box and leucine-rich protein 22*	-2.48
L12_85641	*F-box only protein 25*	2.87
L12_87406	*F-box only protein 38*	2.63
L2_42852	*F-box only protein 43-like*	2.22
L12_45478	*F-box/LRR-repeat protein 4*	1.64
L1_68866	*F-box/LRR-repeat protein 4-like*	1.71
L12_88391	*F-box-like/WD repeat-containing protein TBL1X*	-1.59

L12_74251	*Fc fragment of IgG binding protein*	-4.88
L12_73672	*Fc receptor-like protein 5-like*	3.04
L2_45870	*FCH domain only protein 2*	-2.65
L1_61537	*Fer-1-like protein 6-like*	-1.96
L2_38683	*FERM domain-containing protein 3*	-2.36
L12_83635	*FERM domain-containing protein 4B*	1.66
L1_57403	*Fermitin family homolog 3*	-1.64
L3_71431	*Ferritin, liver middle subunit*	-2.63
L1_44330	*Ferritin, middle subunit*	-2.20
L12_87004	*FGGY carbohydrate kinase domain-containing protein*	1.51
L2_60435	*FH1/FH2 domain-containing protein 1*	1.95
L2_49017	*FH2 domain-containing protein 1*	-2.16
L12_82580	*Fibrinogen C domain-containing protein 1*	1.86
L2_63397	*Fibroblast growth factor 10*	-2.08
L12_71828	*Fibroblast growth factor 7*	2.28
L1_53356	*Fibroblast growth factor receptor 1*	2.53
L1_51253	*Fibroblast growth factor receptor 4*	1.88
L12_63729	*Fibroleukin-like*	-2.02
L2_64143	*Fibromodulin*	-19.09
L1_35298	*Fibronectin*	-1.83
L3_45557	*Fibronectin type III domain-containing protein 3B*	-1.85
L1_26626	*Fibronectin type-III domain-containing protein 3A*	2.27
L2_10076	*Fibronectin-like isoform 1*	-3.38
L12_81105	*Filamin-A*	-2.56
L12_85808	*Filamin-A-interacting protein 1*	1.53
L2_78193	*Filamin-C*	-2.05
L1_38117	*FIS family transcriptional regulator*	-5.28
L3_66045	*Fizzy-related protein homolog*	-1.59
L12_87409	*FK506-binding protein 15*	-1.59
L12_88805	*Flavin reductase-like*	-1.57
L12_89505	*Flotillin-1*	-1.57
L12_70142	*Flotillin-2*	-3.55
L1_23632	*Flp pilus assembly protein ATPase CpaE*	1.65
L1_38834	*Fms-related tyrosine kinase 4*	1.62
L2_52528	*Focal adhesion kinase 1*	-1.99
L12_69588	*Folate receptor beta*	1.76
L12_90042	*Folate transporter 1*	19.23
L12_82810	*Folliculin*	-1.96
L2_61149	*Folliculin-interacting protein 1*	-1.66
L2_36925	*Folliculin-like*	-1.91
L2_65888	*Folylpolyglutamate synthase, mitochondrial*	1.89
L2_87575	*Forkhead box protein D2*	-95.66
L12_84829	*Forkhead box protein F2*	-1.88
L12_56883	*Forkhead box protein K2*	-1.78
L12_81920	*Forkhead box protein P1*	-4.06

L12_88474	*Forkhead box protein P1-B*	-1.51
L2_48104	*Formamidopyrimidine-DNA glycosylase*	1.58
L3_42071	*Formimidoyltransferase-cyclodeaminase*	5.87
L2_52992	*Formin-binding protein 1*	-1.50
L2_32177	*Formin-binding protein 1-like*	-2.99
L12_80662	*Formin-like protein 1*	-1.92
L2_6384	*Formin-like protein 1-like*	-2.00
L1_28131	*Formyl trans N*	-2.06
L12_82149	*Four and a half LIM domains protein 3*	-3.57
L1_46467	*Four-jointed box protein 1*	3.40
L12_73131	*Free fatty acid receptor 2*	-4.73
L1_26060	*Frizzled-10*	-2.14
L3_64405	*Fructose-bisphosphate aldolase A*	1.65
L3_62468	*Fructose-bisphosphate aldolase B*	3.98
L12_87480	*Fructose-bisphosphate aldolase C-B*	-2.04
L1_49228	*FSD1-like protein*	2.48
L12_80609	*Fucolectin*	131.49
L12_82484	*Fucose-1-phosphate guanylyltransferase*	-1.59
L12_87495	*Fumarate hydratase, mitochondrial*	1.61
L1_41618	*Fumarylacetoacetate (FAA) hydrolase family protein*	2.37
L1_19996	*Furin-1*	-1.78
L1_44063	*Furin-1-like*	-1.63
L12_85180	*FXYD domain-containing ion transport regulator 6*	-1.99
L2_34895	*FXYD5a*	-3.12
L2_32109	*FYVE and coiled-coil domain-containing protein 1-like*	1.51
L12_84551	*G kinase-anchoring protein 1*	-2.06
L12_72412	*G patch domain-containing protein 3*	-2.51
L2_46927	*G protein-coupled receptor kinase 5*	-3.94
L2_59124	*G protein-coupled receptor kinase 6*	-1.90
L12_71763	*G1/S-specific cyclin-D2*	1.55
L2_62826	*G2/M phase-specific E3 ubiquitin-protein ligase*	1.55
L12_89683	*Galactocerebrosidase*	-8.73
L2_50834	*Galactosylceramide sulfotransferase*	-1.79
L3_80517	*Galanin receptor type 1*	1.94
L3_48037	*Galectin-1*	-4.89
L12_87326	*Galectin-3-binding protein A*	1.56
L12_81250	*Galectin-9*	-1.91
L1_47762	*Galectin-related protein B*	-1.61
L2_67641	*Gametocyte-specific factor 1*	-16.25
L12_89845	*Gamma-adducin*	-1.86
L2_51203	*Gamma-aminobutyric acid receptor subunit beta-2*	1.82
L1_34346	*Gamma-aminobutyric acid type B receptor subunit 1*	6.27
L2_65531	*Gamma-enolase*	-1.67
L12_89988	*Gamma-glutamyl hydrolase*	-4.20
L3_49413	*Gamma-glutamylaminecyclotransferase B*	2.65

L12_88295	*Gamma-glutamyltransferase 5*	-1.71
L2_67907	*Gamma-glutamyltransferase 5-like*	-27.20
L12_89467	*Gamma-glutamyltranspeptidase 1*	-4.41
L12_73351	*Gamma-parvin*	-1.73
L12_53951	*Gamma-secretase subunit PEN-2*	-1.90
L2_33500	*Gamma-secretase-activating protein*	-1.71
L2_64490	*Gamma-tubulin complex component 4*	-1.50
L12_88728	*Ganglioside GM2 activator*	-4.56
L1_41854	*Gap junction beta-1 protein*	-1.59
L2_35259	*Gap junction beta-2 protein*	-2.06
L3_80160	*Gap junction beta-3 protein*	-1.58
L12_80358	*Gap junction beta-4 protein*	-1.91
L12_76804	*Gap junction Cx32.2 protein*	11.38
L12_83354	*GAS2-like protein 2*	-1.76
L12_84721	*Gastric intrinsic factor-like*	-124.94
L1_32270	*Gastrin*	280.86
L2_73008	*Gastrotropin*	-52.48
L1_40519	*Gastrula zinc finger protein XlCGF67.1*	-1.70
L12_85179	*GATA-binding factor 5-A*	34.00
L12_89553	*GATA-binding factor 6-A*	4.03
L12_38171	*GATA-binding factor 6-A-like*	2.73
L2_61331	*GDH/6PGL endoplasmic bifunctional protein*	-2.04
L12_84988	*GDP-L-fucose synthase*	-1.61
L12_88843	*Gelsolin*	87.96
L12_81284	*Geminin*	-8.31
L12_64523	*GEM-interacting protein*	-1.50
L3_77973	*General vesicular transport factor p115*	1.92
L2_46144	*Germ cell-specific gene 1-like protein*	2.01
L1_11702	*GG22060*	1.91
L3_65312	*Glioma pathogenesis-related protein 1*	-2.41
L12_86593	*Glioma tumor suppressor candidate region gene 2 protein*	1.50
L12_76394	*Gliomedin*	-2.05
L12_86427	*GLIPR1-like protein 1*	-2.40
L3_69839	*Glucagon family neuropeptides*	2.76
L12_71059	*Glucagon-1*	4.86
L12_86607	*Glucocorticoid modulatory element-binding protein 1*	-2.24
L1_28505	*Glucokinase regulatory protein*	1.52
L12_47132	*Glucosamine-6-phosphate isomerase 2*	-2.38
L1_29498	*Glucose-6-phosphatase*	-2.61
L12_86395	*Glucose-6-phosphate isomerase*	1.92
L12_85884	*Glucose-6-phosphate translocase*	2.36
L2_47647	*Glucose-fructose oxidoreductase domain-containing protein 1*	1.66
L12_89868	*Glucosylceramidase*	-2.35
L1_65032	*Glucuronokinase 1*	1.76

L1_40302	*Glutamate*	5.47
L12_88897	*Glutamate carboxypeptidase 2*	2.03
L1_33915	*Glutamate decarboxylase 1*	2.45
L1_40557	*Glutamate decarboxylase-like protein 1*	8.84
L1_11448	*Glutamate dehydrogenase, mitochondrial*	4.05
L3_72416	*Glutamate receptor, ionotropic kainate 4*	2.54
L1_60442	*Glutamate-ammonia-ligase adenylyltransferase*	1.81
L12_81701	*Glutamate-cysteine ligase catalytic subunit*	-2.41
L12_74689	*Glutamate-cysteine ligase regulatory subunit*	-1.54
L1_84569	*Glutaminase kidney isoform, mitochondrial*	3.64
L3_79169	*Glutaminase kidney isoform, mitochondrial '*	1.96
L1_34420	*Glutamine amidotransferases class-II family protein*	1.55
L1_19577	*Glutamine-fructose-6-phosphate aminotransferase*	2.53
L12_77345	*Glutamine-fructose-6-phosphate aminotransferase 2*	3.20
L1_30480	*Glutaredoxin*	1.83
L12_61659	*Glutaredoxin-1*	1.77
L3_45741	*Glutaredoxin-2, mitochondrial*	1.52
L12_72024	*Glutathione peroxidase 3*	3.58
L1_16203	*Glutathione reductase, cytosolic*	1.58
L2_21739	*Glutathione S-transferase*	-4.21
L3_65978	*Glutathione S-transferase A*	1.93
L3_70754	*Glutathione S-transferase omega-1*	-1.67
L12_31364	*Glutathione S-transferase theta 1b*	4.87
L4_70231	*Glyceraldehyde-3-phosphate dehydrogenase*	3.35
L12_85517	*Glycerol-3-phosphate acyltransferase 3-like*	2.95
L2_6940	*Glycerol-3-phosphate dehydrogenase*	2.63
L12_86666	*Glycerol-3-phosphate dehydrogenase , cytoplasmic*	3.91
L12_85607	*Glycerol-3-phosphate dehydrogenase 1-like protein*	2.01
L2_31014	*Glycerol-3-phosphate dehydrogenase, mitochondrial*	-1.86
L2_52067	*Glycerophosphodiester phosphodiesterase domain-containing protein 5*	5.59
L1_75798	*Glycine amidinotransferase, mitochondrial*	141.98
L3_61807	*Glycine cleavage system H protein, mitochondrial*	13.92
L1_88208	*Glycine dehydrogenase , mitochondrial*	83.04
L2_43490	*Glycine receptor subunit alphaZ1*	1.53
L12_88364	*Glycogen synthase, muscle*	1.52
L3_58592	*Glycogen phosphorylase, brain form*	3.17
L12_64530	*Glycogen phosphorylase, liver form*	3.67
L12_87633	*Glycogenin-1-like*	1.53
L2_63028	*Glycolipid transfer protein*	-4.39
L2_32931	*Glycoprotein-N-acetylgalactosamine 3-beta-galactosyltransferase 1-A*	-14.30
L1_17425	*Glycosyl transferase 2 family protein*	1.61
L3_72931	*Glyoxalase domain-containing protein 4*	2.03
L12_65798	*Glyoxalase domain-containing protein 5*	1.61

L12_81089	*Glypican-6*	2.23
L12_87011	*GMP reductase 2*	-1.54
L12_85066	*Golgi SNAP receptor complex member 2*	2.20
L2_58287	*Golgi-associated PDZ and coiled-coil motif-containing protein*	-1.52
L3_40699	*Golgin subfamily A member 7*	1.67
L12_80468	*GPI mannosyltransferase 1*	-1.65
L1_26329	*G-protein coupled bile acid receptor 1*	-1.58
L2_28627	*G-protein coupled receptor 124-like*	-1.51
L1_62406	*G-protein coupled receptor 126*	-1.95
L12_88989	*G-protein coupled receptor 183*	-2.08
L2_21235	*G-protein coupled receptor 1-like*	-3.97
L2_59414	*G-protein coupled receptor 20-like*	2.61
L12_87978	*G-protein coupled receptor 39*	7.92
L12_50688	*G-protein coupled receptor 4-like*	2.01
L4_87942	*G-protein coupled receptor 54*	1.82
L1_52138	*G-protein coupled receptor 6*	-1.97
L12_87507	*G-protein coupled receptor 64*	2.24
L12_83810	*G-protein coupled receptor 64-like*	1.69
L3_79308	*G-protein coupled receptor 84*	-1.73
L12_84368	*G-protein coupled receptor family C group 5 member C*	1.54
L1_10634	*G-protein-signaling modulator 1*	2.59
L2_25373	*GRAM domain-containing protein 1B*	-3.17
L2_39225	*GRAM domain-containing protein 1C*	2.01
L1_66502	*GRAM domain-containing protein 3-like*	19.26
L12_62392	*Granulins*	-1.92
L1_43082	*Granulins-like*	-48.21
L12_76315	*Granzyme B*	1.50
L2_62527	*Granzyme-like protein 1*	-1.67
L3_70590	*Granzyme-like protein 2*	-1.61
L12_85119	*Graves disease carrier protein*	-2.24
L12_84565	*GRB2-related adaptor protein 2*	-1.86
L1_18942	*Gremlin-1-like*	-2.11
L3_77202	*G-rich sequence factor 1*	1.51
L2_49692	*GRINL1A complex locus protein 1-like*	-32.12
L1_71671	*Group 1 glycosyl transferase*	1.86
L12_73756	*Group XIIB secretory phospholipase A2-like protein*	19.43
L2_38164	*Group XV phospholipase A2*	-2.21
L12_80034	*Growth arrest and DNA damage-inducible protein GADD45 alpha*	-1.56
L2_50143	*Growth arrest and DNA damage-inducible protein GADD45 beta*	-4.52
L2_65464	*Growth arrest-specific protein 2*	2.87
L12_81765	*Growth arrest-specific protein 6*	-1.57
L12_40219	*Growth differentiation factor 15*	4.11

L12_59549	*Growth factor receptor-bound protein 2*	2.67
L12_82823	*Growth hormone-inducible transmembrane protein*	1.62
L12_90052	*Growth hormone-regulated TBC protein 1-A*	2.97
L1_27110	*Growth/differentiation factor 15*	14.43
L1_46336	*Growth/differentiation factor 6-A*	-1.73
L4_73172	*Growth/differentiation factor 8*	-11.08
L1_32523	*GTPase IMAP family member 7-like*	4.24
L12_70136	*GTPase KRas*	1.78
L1_57888	*GTP-binding protein 1*	1.92
L12_76471	*GTP-binding protein 10*	1.60
L12_86689	*GTP-binding protein 8*	-2.11
L2_83264	*GTP-binding protein Rhes*	-5.95
L12_87929	*Guanidinoacetate N-methyltransferase*	7.99
L12_88842	*Guanine deaminase*	-1.80
L1_53629	*Guanine nucleotide exchange factor DBS*	-2.24
L12_88676	*Guanine nucleotide exchange factor VAV3*	-1.62
L1_84040	*Guanine nucleotide-binding protein G(i) subunit alpha-2*	4.69
L12_73828	*Guanine nucleotide-binding protein G(I)/G(S)/G(O) subunit gamma-2*	-1.59
L12_83899	*Guanine nucleotide-binding protein G(o) subunit alpha*	1.96
L12_86709	*Guanine nucleotide-binding protein subunit alpha-14*	2.24
L12_83183	*Guanine nucleotide-binding protein subunit beta-5*	2.74
L1_40311	*Guanine nucleotide-binding protein subunit beta-like protein 1*	1.91
L2_62342	*Guanine nucleotide-binding protein-like 1*	3.01
L12_75761	*Guanylate cyclase soluble subunit alpha-2*	-2.34
L12_30986	*Guanylate cyclase soluble subunit alpha-3*	-2.36
L2_19610	*Guanylate cyclase soluble subunit beta-1*	-1.56
L3_59343	*Guanylate kinase*	-1.65
L1_33737	*Guanylyl cyclase-activating protein 1-like*	-5.68
L12_87089	*H(+)/Cl(-) exchange transporter 5*	-1.75
L12_83637	*H(+)/Cl(-) exchange transporter 7*	-2.14
L3_67066	*H-2 class II histocompatibility antigen, A-F alpha chain*	-1.65
L2_53250	*Hamartin*	1.68
L2_66471	*HAUS augmin-like complex subunit 6-like*	4.14
L2_31264	*HEAT repeat-containing protein 2*	2.55
L12_76021	*HEAT repeat-containing protein 7A*	-3.07
L1_54658	*Heat shock 70 kDa protein 12A*	-1.50
L2_66203	*Heat shock protein 30-like*	1.53
L12_88903	*Heat-stable enterotoxin receptor*	2.56
L12_80561	*Helicase-like transcription factor*	1.51
L12_74975	*Heme oxygenase*	-2.13
L2_67695	*Heme-binding protein 2*	-13.24
L1_35911	*Hemicentin-1*	-1.53
L3_78520	*Hemoglobin subunit alpha-D*	2.30

L3_35102	*Hemoglobin subunit beta*	1.65
L12_90258	*Hemoglobin subunit beta-2*	5.46
L12_89073	*Heparan sulfate glucosamine 3-O-sulfotransferase 3B1*	2.17
L12_86036	*Heparan-alpha-glucosaminide N-acetyltransferase*	1.54
L1_43577	*Heparan-alpha-glucosaminide N-acetyltransferase-like*	1.98
L12_77673	*Hepatitis A virus cellular receptor 1 homolog*	1.80
L2_68040	*Hepatocyte growth factor*	-2.09
L1_53175	*Hepatocyte growth factor receptor*	-2.92
L2_40668	*Hepatocyte growth factor receptor-like*	1.50
L2_47737	*Hepatocyte growth factor-regulated tyrosine kinase substrate*	-1.70
L1_32063	*Hepatocyte nuclear factor 1-alpha*	1.63
L3_58789	*Hepatocyte nuclear factor 3-beta*	2.50
L12_88040	*Hepatocyte nuclear factor 4-alpha*	2.05
L12_85830	*Hepatocyte nuclear factor 4-beta*	91.49
L12_88915	*Hepatocyte nuclear factor 4-gamma*	3.76
L12_79856	*Hepatoma-derived growth factor-related protein 3*	-2.22
L12_88975	*Hermansky-Pudlak syndrome 1 protein homolog*	-2.69
L2_51373	*Hermansky-Pudlak syndrome 3 protein*	-2.33
L2_67128	*Hermansky-Pudlak syndrome 4 protein*	-2.29
L12_85057	*Hermansky-Pudlak syndrome 5 protein*	-2.45
L2_66974	*Heterogeneous nuclear ribonucleoprotein U-like protein 2*	1.53
L12_87745	*Hexokinase-1*	-2.37
L12_80919	*Hexosaminidase D*	-1.92
L3_56606	*HIG1 domain family member 1A, mitochondrial*	-3.17
L1_66067	*High affinity cAMP-specific and IBMX-insensitive 3',5'-cyclic phosphodiesterase 8A*	1.68
L2_43558	*High affinity cGMP-specific 3',5'-cyclic phosphodiesterase 9A-like*	-5.20
L12_62568	*High affinity choline transporter 1*	1.99
L3_57662	*High affinity copper uptake protein 1*	5.80
L12_78973	*High affinity immunoglobulin gamma Fc receptor I-like*	2.87
L12_72344	*High choriolytic enzyme 1*	161.58
L12_74246	*High mobility group protein B1*	-1.50
L2_49112	*High-affinity branched-chain amino acid transport system permease protein BraE*	3.19
L3_13893	*High-affinity lysophosphatidic acid receptor*	2.59
L1_70061	*Hippocampus abundant transcript 1 protein*	-1.66
L12_80449	*HIRA-interacting protein 3*	-1.83
L12_89017	*Histamine H3 receptor*	1.89
L12_81681	*Histamine N-methyltransferase*	4.16
L12_73377	*Histamine N-methyltransferase A*	7.66
L2_88047	*Histidine ammonia-lyase*	-395.29
L12_75668	*Histidine protein methyltransferase 1 homolog*	1.95
L12_39107	*Histidine triad nucleotide-binding protein 1*	-1.87

L1_14472	*Histidine-rich glycoprotein*	1.52
L1_61373	*Histone chaperone asf1b-A*	1.82
L12_85279	*Histone deacetylase 10-like*	-1.95
L1_70007	*Histone deacetylase 5*	-1.50
L12_63173	*Histone deacetylase 6*	2.54
L12_75604	*Histone deacetylase 7*	-1.50
L2_27717	*Histone deacetylase complex subunit SAP130*	1.58
L3_32899	*Histone deacetylase complex subunit SAP18*	-1.52
L12_69358	*Histone deacetylase complex subunit SAP30L*	-1.81
L1_63260	*Histone H1oo*	30.61
L3_77339	*Histone H2A*	1.52
L12_84627	*Histone H2A.V*	-1.50
L12_83854	*Histone-binding protein N1/N2*	-2.26
L2_65901	*Histone-lysine N-methyltransferase EHMT1*	-2.31
L2_64753	*Histone-lysine N-methyltransferase NSD2*	2.96
L2_58119	*Histone-lysine N-methyltransferase SETDB1-B*	2.04
L2_63576	*HLA class II histocompatibility antigen, DQ beta 1 chain*	-8.21
L2_49128	*Homeobox protein aristaless-like 4*	80.26
L1_4673	*Homeobox protein CDX-1*	2.29
L12_71728	*Homeobox protein CHOX-CAD*	-2.01
L3_77961	*Homeobox protein Dlx2b*	1.70
L12_73753	*Homeobox protein Hox-A11a*	-41.07
L2_62478	*Homeobox protein Hox-A13a*	-113.58
L2_32647	*Homeobox protein Hox-A13b*	-23.82
L2_36015	*Homeobox protein Hox-A2b*	-11.66
L12_84463	*Homeobox protein Hox-A3a*	-2.55
L12_74308	*Homeobox protein Hox-A4*	-2.08
L12_69345	*Homeobox protein Hox-A7*	-3.34
L12_47668	*Homeobox protein Hox-A9*	-18.23
L2_27317	*Homeobox protein Hox-C11*	-4.59
L2_27519	*Homeobox protein Hox-C11a*	-2.23
L2_71564	*Homeobox protein Hox-C13a*	-118.63
L2_57621	*Homeobox protein Hox-C4*	-3.28
L1_32385	*Homeobox protein Hox-C4a*	-3.82
L2_74016	*Homeobox protein Hox-C9*	-8.72
L2_41473	*Homeobox protein Hox-D4b*	-50.83
L2_44506	*Homeobox protein Hox-D9a*	-5.87
L2_47717	*Homeobox protein Mohawk*	-2.74
L1_39198	*Homeobox protein MSH-D-like*	2.29
L12_84390	*Homeobox protein Nkx-2.5*	-1.81
L1_44891	*Homeobox protein PKNOX2*	4.54
L3_36550	*Homeodomain-only protein*	-5.66
L12_46873	*Homer protein homolog 2*	2.22
L12_80209	*Homocysteine S-methyltransferase 1*	2.69
L12_83469	*Homocysteine S-methyltransferase YbgG*	1.94

L2_74242	*Homogentisate 1,2-dioxygenase*	-7.53
L1_53630	*Hormonally up-regulated neu tumor-associated kinase homolog A*	2.90
L2_5373	*Hormone recep*	1.77
L12_73755	*Hormone-sensitive lipase*	1.57
L1_26589	*Hyaluronan and proteoglycan link protein 4*	2.74
L12_82617	*Hyaluronidase-1*	-5.56
L12_73507	*Hyaluronidase-2*	-1.54
L1_9305	*Hydroxyacid-oxoacid transhydrogenase, mitochondrial*	2.63
L2_40260	*Hydroxycarboxylic acid receptor 2-like*	-1.78
L2_75997	*Hydroxylysine kinase*	-42.33
L12_80901	*Hypothetical protein LOC100270729*	-2.04
L1_24926	*Hypothetical protein LOC100330808*	2.05
L12_86261	*Hypothetical protein LOC100486905*	2.29
L2_64764	*Hypothetical protein LOC100496506, partial*	1.56
L12_82470	*Hypothetical protein LOC100690397*	10.04
L2_45203	*Hypothetical protein LOC100690489, partial*	-1.59
L2_66632	*Hypothetical protein LOC100690563*	-1.67
L1_34753	*Hypothetical protein LOC100690681*	10.59
L12_74427	*Hypothetical protein LOC100690789*	1.66
L12_84394	*Hypothetical protein LOC100690846*	-2.17
L12_74353	*Hypothetical protein LOC100691594*	-2.60
L12_56135	*Hypothetical protein LOC100692312*	-1.92
L2_59349	*Hypothetical protein LOC100692388*	-1.58
L1_39347	*Hypothetical protein LOC100692741*	2.21
L2_43023	*Hypothetical protein LOC100693489*	-16.17
L12_83846	*Hypothetical protein LOC100695942*	-7.45
L12_79098	*Hypothetical protein LOC100695983*	2.19
L3_38390	*Hypothetical protein LOC100696022*	1.79
L1_41131	*Hypothetical protein LOC100696895*	-1.68
L2_40593	*Hypothetical protein LOC100697049*	-1.68
L1_38642	*Hypothetical protein LOC100697472*	2.07
L2_32472	*Hypothetical protein LOC100697493*	-1.60
L2_46599	*Hypothetical protein LOC100698366*	-13.19
L1_71411	*Hypothetical protein LOC100698473*	1.87
L2_50910	*Hypothetical protein LOC100699558*	-3.31
L12_82732	*Hypothetical protein LOC100699670*	556.59
L1_33760	*Hypothetical protein LOC100700543*	-2.59
L2_62623	*Hypothetical protein LOC100700562*	-2.98
L2_66209	*Hypothetical protein LOC100700692*	2.37
L2_37631	*Hypothetical protein LOC100701547*	3.02
L2_24132	*Hypothetical protein LOC100701611*	-3.00
L1_17509	*Hypothetical protein LOC100701644*	-1.84
L1_64001	*Hypothetical protein LOC100702553*	3.29
L2_24007	*Hypothetical protein LOC100703757*	1.70

L2_61594	*Hypothetical protein LOC100703766*	-2.19
L2_38256	*Hypothetical protein LOC100703912*	-2.06
L2_45624	*Hypothetical protein LOC100703934*	-1.94
L12_71054	*Hypothetical protein LOC100704118*	-1.79
L2_44547	*Hypothetical protein LOC100704132, partial*	1.63
L2_21471	*Hypothetical protein LOC100704684*	-1.94
L2_23329	*Hypothetical protein LOC100704727*	-4.80
L2_25068	*Hypothetical protein LOC100705020*	-1.54
L2_37458	*Hypothetical protein LOC100705062*	3.18
L2_34994	*Hypothetical protein LOC100705374*	-1.59
L1_53879	*Hypothetical protein LOC100706280*	1.73
L1_60069	*Hypothetical protein LOC100706652*	3.02
L2_16573	*Hypothetical protein LOC100708183*	9.67
L1_43942	*Hypothetical protein LOC100709207*	-5.14
L12_73066	*Hypothetical protein LOC100709459*	-1.83
L1_19450	*Hypothetical protein LOC100710859*	2.00
L1_6273	*Hypothetical protein LOC100710874*	2.30
L1_29215	*Hypothetical protein LOC100711525*	-2.12
L12_85693	*Hypothetical protein LOC100711564*	-1.50
L1_10294	*Hypothetical protein LOC100711695*	3.48
L2_25932	*Hypothetical protein LOC798447*	1.80
L2_21633	*Hypothetical protein SechA1 07655*	1.88
L1_40395	*Hypothetical protein SechA1 18919*	1.54
L1_7511	*Hypothetical protein SMAC 09862*	1.55
L1_5555	*Hypothetical protein SPAM266 11276*	1.56
L2_8704	*Hypothetical protein SUS17 998*	4.05
L1_42473	*Hypothetical protein VOLCADRAFT 91554*	-1.80
L12_23959	*Hypothetical protein XF0051*	2.46
L12_68444	*Hypoxia-inducible factor 1-alpha inhibitor*	1.67
L12_89623	*Iduronate 2-sulfatase*	-1.91
L2_38812	*IFI56 interferon-induced protein*	9.11
L12_88285	*Ig heavy chain C region, secreted form*	-14.61
L4_22668	*Ig heavy chain V region XIG14*	-2.09
L2_35113	*Ig heavy chain V-III region HIL-like*	-2.77
L2_54128	*Ig heavy chain V-III region WEA-like*	-2.54
L2_62633	*Ig kappa chain V region 3547*	-2.71
L3_54312	*Ig kappa chain V-III region CLL*	-4.80
L2_58369	*Ig kappa chain V-III region MOPC 63-like*	-4.74
L2_42715	*Ig kappa chain V-III region VG*	-3.13
L2_25705	*Ig lambda chain V-VI region SUT-like*	-2.56
L12_84881	*IgGFc-binding protein*	22.72
L2_88118	*Ileal sodium/bile acid cotransporter*	-125.59
L12_76889	*Immunoglobulin lambda-like polypeptide 1*	-6.82
L2_21217	*Immunoglobulin lambda-like polypeptide 1-like*	-1.73

L2_21629	*Immunoglobulin superfamily containing leucine-rich repeat protein 2*	-1.55
L3_75954	*Immunoglobulin superfamily member 10*	-2.76
L12_80473	*Immunoglobulin tau-5 heavy chain constant region*	-4.77
L2_75459	*Immunoglobulin-like and fibronectin type III domain-containing protein 1*	-757.80
L12_85662	*Inactive hydroxysteroid dehydrogenase-like protein 1*	1.86
L2_28496	*Inactive phospholipase C-like protein 1*	-2.59
L12_79456	*Inactive phospholipase C-like protein 2-like*	-1.89
L3_79064	*Inactive rhomboid protein 1*	1.52
L2_21474	*Inactive serine protease 35*	-2.15
L1_46900	*Inactive tyrosine-protein kinase 7*	1.73
L12_82490	*Indoleamine 2,3-dioxygenase 2*	-2.87
L12_86933	*Induced myeloid leukemia cell differentiation protein Mcl-1 homolog*	-2.11
L12_86764	*Influenza virus NS1A-binding protein homolog A*	1.55
L3_79301	*Inner centromere protein*	-1.63
L12_76473	*Inorganic pyrophosphatase*	4.04
L12_82974	*Inosine-5'-monophosphate dehydrogenase 2*	1.60
L1_27404	*Inositol 1,4,5-triphosphate receptor-interacting protein-like*	-1.99
L2_60873	*Inositol 1,4,5-trisphosphate receptor type 3*	1.71
L1_5069	*Inositol hexakisphosphate and diphosphoinositol-pentakisphosphate kinase 1*	1.71
L1_30024	*Inositol hexakisphosphate kinase 2*	-1.83
L2_58381	*Inositol monophosphatase 1*	-3.41
L2_22214	*Inositol polyphosphate 5-phosphatase K*	-1.56
L12_76567	*Inositol polyphosphate 5-phosphatase OCRL-1-like*	1.87
L2_62165	*Inositol-trisphosphate 3-kinase A*	-1.68
L12_82048	*Insulin receptor substrate 2*	2.87
L12_81428	*Insulin-like growth factor 1 receptor*	-1.72
L3_25255	*Insulin-like growth factor 2 mRNA-binding protein 2*	-2.79
L1_52046	*Insulin-like growth factor 2 mRNA-binding protein 3*	2.27
L4_39424	*Insulin-like growth factor II*	4.44
L12_72195	*Insulin-like growth factor-binding protein 4*	1.66
L12_73494	*Insulin-like growth factor-binding protein 5-like*	2.74
L1_65394	*Insulin-like growth factor-binding protein complex acid labile subunit*	1.82
L1_30900	*Integral membrane protein GPR137B*	-2.71
L2_11758	*Integral membrane sensor signal transduction histidine kinase*	1.61
L2_30060	*Integrin alpha2*	-2.04
L3_73793	*Integrin alpha-6*	2.54
L2_33710	*Integrin beta-5*	1.84
L12_85889	*Integrin-linked protein kinase*	-1.81

L1_88128	*Intelectin-2*	112.95
L12_46641	*Interactor protein for cytohesin exchange factors 1*	-1.56
L1_59746	*Inter-alpha-trypsin inhibitor heavy chain H3*	-2.50
L2_70346	*Inter-alpha-trypsin inhibitor heavy chain H3-like*	1.75
L4_71803	*Interferon*	-3.28
L12_81481	*Interferon regulatory factor 4*	2.71
L12_81491	*Interferon-induced transmembrane protein 10-like*	2.29
L3_7773	*Interferon-induced transmembrane protein 5-like*	-1.74
L12_84152	*Interferon-induced, double-stranded RNA-activated protein kinase*	-1.72
L4_65790	*Interferon-inducible transmembrane protein 1 protein*	-2.40
L1_45467	*Interferon-related developmental regulator 1*	1.61
L2_60323	*Interleukin-1 receptor type 2-like*	3.62
L12_84979	*Interleukin-1 receptor-associated kinase 4*	-1.73
L3_62316	*Interleukin-10*	-2.11
L1_27613	*Interleukin-10-like*	-2.08
L12_65520	*Interleukin-12 receptor subunit beta-1*	-1.58
L12_80471	*Interleukin-12 subunit beta*	-2.13
L12_78508	*Interleukin-17 receptor C*	-1.87
L2_9736	*Interleukin-17 receptor D*	-1.69
L12_82454	*Interleukin-18 receptor 1*	-1.87
L12_76161	*Interleukin-2 receptor subunit beta*	-1.51
L12_87822	*Interleukin-21 receptor*	-2.22
L12_82493	*Interleukin-22 receptor subunit alpha-2*	-5.56
L12_77063	*Interleukin-6 receptor subunit alpha*	-2.40
L4_54120	*Interleukin-8*	-3.77
L12_89446	*Intersectin-2*	1.66
L12_86353	*Intestinal-type alkaline phosphatase*	4.09
L2_69020	*Intestine-specific homeobox*	71.35
L12_73810	*Intraflagellar transport protein 20 homolog*	1.68
L12_71185	*Inverted formin-2-like*	1.97
L1_59855	*IQ motif and SEC7 domain-containing protein 2*	-5.49
L12_87058	*Iron/zinc purple acid phosphatase-like protein*	-12.48
L12_81664	*Isoamyl acetate-hydrolyzing esterase 1 homolog*	1.90
L1_61586	*Isocitrate dehydrogenase subunit alpha, mitochondrial*	-1.84
L12_84446	*Isocitrate dehydrogenase , mitochondrial*	-2.67
L12_87217	*Isoprenoid synthase domain-containing protein*	1.55
L1_43578	*Isotocin receptor*	2.37
L3_21590	*Isotocin-neurophysin IT 1*	3.87
L12_83392	*Isthmin-1*	-6.03
L3_75512	*Izumo sperm-egg fusion protein 1-like*	2.07
L1_25021	*Janus kinase and microtubule-interacting protein 1*	1.58
L3_79619	*JmjC domain-containing protein 7*	1.75
L12_79513	*JmjC domain-containing protein 8*	-4.53
L12_89527	*Junction plakoglobin*	1.67

L1_26774	*Junction-mediating and -regulatory protein*	-2.08
L1_7147	*Kalirin-like*	1.56
L2_62237	*Kazrin*	-1.85
L12_86451	*KDEL motif-containing protein 2*	-1.62
L2_64559	*Kelch domain-containing protein 10-like*	-4.04
L1_49560	*Kelch domain-containing protein 7A*	1.59
L12_87201	*Kelch repeat and BTB domain-containing protein 11*	-5.96
L2_65191	*Kelch repeat and BTB domain-containing protein 12*	-4.39
L12_83215	*Kelch repeat and BTB domain-containing protein 13*	-1.61
L3_76379	*Kelch-like protein 23*	-1.98
L3_77926	*Kelch-like protein 4*	1.77
L12_87596	*Kelch-like protein 6*	-1.90
L12_81020	*Keratin, type I cytoskeletal 13*	1.72
L12_85741	*Keratin, type II cytoskeletal 8*	1.87
L2_59300	*Keratin-like protein KRT222*	-5.15
L12_89644	*Keratinocyte-associated protein 3*	2.37
L12_80431	*Ketohexokinase*	2.00
L12_78534	*Kidney mitochondrial carrier protein 1*	-2.28
L12_83670	*Kinase D-interacting substrate of 220 kDa*	-3.58
L12_72695	*Kinase isozyme 2, mitochondrial*	1.86
L1_6891	*Kinase isozyme 4, mitochondrial*	1.56
L2_28631	*Kinase suppressor of Ras 1-like*	-1.54
L2_31260	*Kinase, mitochondrial*	2.15
L12_89735	*Kinectin*	1.57
L1_28166	*Kinesin light chain 1*	-1.64
L2_57636	*Kinesin-1 heavy chain*	-1.57
L2_49291	*Kinesin-like protein KIF16B*	-1.88
L1_26584	*Kinesin-like protein KIF20B-like*	1.72
L2_33797	*Kinesin-like protein KIF21A*	1.74
L12_59925	*Kinesin-like protein KIFC3*	8.30
L12_84925	*Kinetochore-associated protein DSN1 homolog*	2.60
L12_86569	*Kininogen*	2.44
L1_73561	*Klotho*	2.46
L12_84392	*KN motif and ankyrin repeat domain-containing protein 1*	5.76
L2_51556	*Kremen protein 1*	-2.08
L12_58141	*Krueppel-like factor 12*	-1.64
L12_58691	*Krueppel-like factor 4*	1.62
L12_86790	*Kv channel-interacting protein 1*	-2.34
L3_79117	*Kynureninase*	1.59
L12_86123	*Kynurenine formamidase*	-1.70
L12_81954	*Kynurenine-oxoglutarate transaminase 1*	7.00
L12_87785	*L-2-hydroxyglutarate dehydrogenase, mitochondrial*	1.98
L1_13092	*Lactadherin*	2.01
L12_89397	*Lactase-phlorizin hydrolase*	462.75
L12_75876	*Lactation elevated protein 1 homolog B*	-1.57

L12_61029	*Lactation elevated protein 1 homolog B-like*	-2.02
L12_73202	*Lactose-binding lectin l-2*	18.18
L12_88434	*Lactosylceramide 1,3-N-acetyl-beta-D-glucosaminyltransferase A*	-2.19
L3_68651	*Lactoylglutathione lyase*	1.73
L12_79402	*Lambda-crystallin homolog*	-2.96
L1_44533	*Lamin-A*	4.05
L3_53423	*Lamina-associated polypeptide 2, isoforms beta/delta/epsilon/gamma-like*	-1.94
L12_83947	*Lamin-B1*	-1.77
L1_4089	*Laminin G 2*	-1.54
L1_18703	*Laminin N*	206.65
L12_90144	*Laminin subunit alpha-3*	2.81
L2_59722	*Laminin subunit alpha-4*	-1.60
L1_48057	*Laminin subunit beta-3*	53.78
L12_73956	*Laminin subunit gamma-3*	-1.64
L12_84704	*L-amino-acid oxidase*	-3.37
L3_79235	*La-related protein 1B*	2.44
L1_51603	*La-related protein 1B-like*	2.32
L12_35367	*La-related protein 4-like*	1.89
L12_89826	*Large neutral amino acids transporter small subunit 4*	4.03
L12_80607	*Latent-transforming growth factor beta-binding protein 3*	-1.56
L1_52395	*Lathosterol oxidase*	2.14
L2_14483	*Ldl recept a*	-10.74
L12_74558	*LDLR chaperone MESD*	-2.34
L12_71183	*Lecithin retinol acyltransferase*	2.71
L2_6843	*Lecithin retinol acyltransferase (phosphatidylcholine--retinol O-acyltransferase)*	-9.32
L2_70565	*Legumain*	-3.18
L12_89859	*Leiomodin-1*	-1.87
L3_66498	*Leiomodin-2*	8.09
L12_72475	*Lethal(3)malignant brain tumor-like protein 4*	1.50
L12_86447	*LETM1 and EF-hand domain-containing protein 1, mitochondrial*	1.70
L1_27940	*Leucine zipper putative tumor suppressor 2*	3.13
L1_39890	*Leucine-rich repeat flightless-interacting protein 1*	2.02
L2_38904	*Leucine-rich repeat serine/threonine-protein kinase 2*	-2.33
L2_51553	*Leucine-rich repeat transmembrane neuronal protein 4*	-1.58
L12_77028	*Leucine-rich repeat transmembrane protein FLRT3*	-1.60
L12_85397	*Leucine-rich repeat-containing protein 1*	1.79
L1_49092	*Leucine-rich repeat-containing protein 14*	1.53
L3_80164	*Leucine-rich repeat-containing protein 2*	1.57
L12_87630	*Leucine-rich repeat-containing protein 33*	-2.43
L2_33158	*Leucine-rich repeat-containing protein 3B*	-2.88
L3_75181	*Leucine-rich repeat-containing protein 43*	1.57

L2_27373	*Leucine-rich repeat-containing protein 45*	-1.51
L1_66063	*Leucine-rich repeat-containing protein 48*	1.72
L12_89261	*Leucine-rich repeat-containing protein 58*	-1.95
L1_55348	*Leucine-rich repeat-containing protein 61*	3.55
L12_73435	*Leucine-rich repeat-containing protein 68-like*	1.55
L2_47524	*Leucine-rich repeat-containing protein C10orf11 homolog*	-2.15
L1_49460	*Leucine-rich repeat-containing protein ENSP00000371558-like*	2.72
L12_84609	*Leucyl-cystinyl aminopeptidase*	-1.68
L2_59859	*Leukemia inhibitory factor receptor-like*	2.43
L1_24577	*Leukocyte immune-type receptor 3*	-1.98
L1_36476	*Leukocyte immune-type receptor TS32.15 L2.1a*	-2.18
L12_87361	*Leukotriene A-4 hydrolase*	3.69
L2_39951	*Leukotriene A-4 hydrolase-like, partial*	4.12
L3_79451	*Leukotriene B4 receptor 1*	1.85
L12_65067	*Leukotriene C4 synthase*	1.73
L2_34440	*LIM and SH3 domain protein 1-like*	-2.08
L12_86746	*LIM domain and actin-binding protein 1*	2.11
L2_69851	*LIM domain kinase 2*	-1.85
L12_82252	*LIM domain only protein 7*	-1.70
L1_53004	*LIM domain only protein 7-like*	2.66
L12_88551	*LIM domain-binding protein 1-A*	-2.97
L1_20254	*LIM domain-binding protein 2*	-1.53
L3_51742	*LIM domain-containing protein 2*	-1.97
L1_24578	*LIM homeobox transcription factor 1-beta*	-3.72
L1_22098	*LIM homeobox transcription factor 1-beta.1*	5.79
L1_43353	*LIM/homeobox protein LMX-1.2*	9.44
L2_70652	*Limb region 1 homolog-like protein*	3.11
L12_87842	*LINE-1 reverse transcriptase homolog*	-3.61
L3_80250	*Linker histone H1M*	2.60
L12_81309	*Lipase member H*	1.90
L12_86518	*Lipid phosphate phosphohydrolase 1*	2.51
L1_61749	*Lipid phosphate phosphohydrolase 3*	-3.56
L2_31709	*Lipoprotein lipase*	-3.65
L1_64043	*Lipoyl synthase, mitochondrial-like*	2.07
L12_72540	*Lissencephaly-1 homolog B*	1.86
L12_73806	*Liver-expressed antimicrobial peptide 2-like*	52.32
L1_40193	*Liver-expressed antimicrobial peptide-2*	3.61
L1_38371	*L-lactate dehydrogenase 2*	1.65
L12_85938	*L-lactate dehydrogenase B chain*	1.51
L12_86732	*LMBR1 domain-containing protein 2-B*	-2.19
L12_69651	*LMBR1 domain-containing protein 2-like*	-2.39
L1_51682	*LON peptidase N-terminal domain and RING finger protein 1-like*	-1.75

L12_85369	*LON peptidase N-terminal domain and RING finger protein 3*	1.64
L12_75367	*Long-chain fatty acid transport protein 4*	3.89
L1_65983	*Long-chain fatty acid transport protein 6*	1.75
L3_76305	*Long-chain-fatty-acid-CoA ligase 3*	3.02
L12_88065	*Long-chain-fatty-acid-CoA ligase 5*	94.01
L3_77718	*Long-chain-fatty-acid-CoA ligase 6*	-3.28
L12_81307	*Long-chain-fatty-acid-CoA ligase ACSBG2*	36.37
L12_39962	*Long-chain-fatty-acid-CoA ligase ACSBG2-like*	21.41
L12_87453	*Low affinity cationic amino acid transporter 2*	2.90
L12_75305	*Low affinity vacuolar monovalent cation/H(+) antiporter*	-1.80
L12_73668	*Low choriolytic enzyme*	-2.73
L1_64540	*Low-density lipoprotein receptor*	-1.98
L12_86414	*Low-density lipoprotein receptor 1*	-4.57
L12_86072	*Low-density lipoprotein receptor-related protein 11*	-21.99
L2_87513	*Low-density lipoprotein receptor-related protein 2*	-20.32
L2_34259	*LrgA family protein*	1.80
L12_66846	*L-rhamnose-binding lectin CSL2*	-9.55
L2_34333	*Lumican*	-2.25
L12_72408	*Lutropin-choriogonadotropic hormone receptor*	-8.71
L12_72738	*Lymphatic vessel endothelial hyaluronic acid receptor 1-like*	-2.06
L12_73259	*Lymphocyte activation gene 3*	-4.37
L12_80285	*Lymphocyte antigen 108 isoform s*	-3.42
L2_32099	*Lymphocyte cytosolic protein 2*	3.59
L2_56357	*Lymphoid-restricted membrane protein*	-1.60
L3_48231	*LYR motif-containing protein 5B*	2.09
L12_47407	*LYR motif-containing protein 7*	-1.53
L12_87019	*LysM and putative peptidoglycan-binding domain-containing protein 3*	2.36
L12_90121	*Lysocardiolipin acyltransferase 1*	1.80
L12_88686	*Lysophosphatidic acid receptor 6*	-1.61
L3_43160	*Lysophosphatidylcholine acyltransferase 2*	-1.78
L2_42954	*Lysophospholipase-like protein 1-like*	1.74
L2_41787	*Lysophospholipid acyltransferase 2*	-1.94
L12_84318	*Lysophospholipid acyltransferase 5*	1.81
L2_38612	*Lysophospholipid acyltransferase LPCAT4*	-1.71
L12_79292	*Lysoplasmalogenase*	28.46
L12_55863	*Lysoplasmalogenase-like protein TMEM86A*	1.94
L12_89720	*Lysosomal acid phosphatase*	-1.75
L12_89880	*Lysosomal alpha-mannosidase*	-7.15
L12_85440	*Lysosomal amino acid transporter 1 homolog*	-1.71
L12_87958	*Lysosomal protective protein*	-10.76
L12_88743	*Lysosomal protein NCU-G1*	-2.27
L12_88698	*Lysosomal Pro-X carboxypeptidase*	-2.74

L2_64876	*Lysosomal thioesterase PPT2-A*	-6.05
L12_74799	*Lysosomal-associated transmembrane protein 4A*	-1.81
L12_57670	*Lysosome membrane protein 2*	-1.60
L12_34937	*Lysosome membrane protein 2-like*	-1.55
L12_82001	*Lysosome-associated membrane glycoprotein 2*	-2.56
L12_66506	*Lysozyme C*	206.34
L4_47403	*Lysozyme g*	-3.47
L12_88489	*M7GpppN-mRNA hydrolase*	-2.34
L12_87296	*Macrophage colony-stimulating factor 1 receptor 1*	1.56
L12_69528	*Macrophage colony-stimulating factor 1 receptor 2*	-1.50
L3_75164	*Macrophage mannose receptor 1-like*	1.99
L12_74787	*Macrophage-capping protein*	-1.77
L1_22292	*Magnesium transporter NIPA2*	2.71
L2_29179	*Magnesium transporter NIPA4*	-1.58
L12_89401	*MAGUK p55 subfamily member 6*	2.81
L12_89545	*Major facilitator superfamily domain-containing protein 1*	-4.00
L12_83918	*Major facilitator superfamily domain-containing protein 10*	-1.92
L2_39317	*Major facilitator superfamily domain-containing protein 12*	-12.92
L1_1331	*Major facilitator superfamily domain-containing protein 1-like*	-3.69
L12_76696	*Major facilitator superfamily domain-containing protein 2A-B*	-3.21
L2_61566	*Major facilitator superfamily domain-containing protein 4*	-20.86
L12_88467	*Major facilitator superfamily domain-containing protein 4-A*	-42.50
L3_77523	*Major facilitator superfamily domain-containing protein 7*	1.58
L12_79900	*Malate dehydrogenase, cytoplasmic*	1.74
L3_58221	*Maleylacetoacetate isomerase*	2.13
L12_90090	*Maltase-glucoamylase, intestinal*	2.42
L1_9399	*MAM and LDL-receptor class A domain-containing protein*	4.97
L12_89585	*MAM and LDL-receptor class A domain-containing protein C10orf112*	3.88
L12_75936	*MAM and LDL-receptor class A domain-containing protein C10orf112-like*	4.96
L4_76668	*Mamu class II histocompatibility antigen, DR alpha chain*	2.49
L3_79032	*Mannan-binding lectin serine protease 1*	3.60
L3_56036	*Mannan-binding lectin serine protease 2*	1.61
L12_82187	*Mannose-P-dolichol utilization defect 1 protein*	-1.54
L1_8838	*Mannosyl-oligosaccharide 1,2-alpha-mannosidase IC*	-4.01
L12_85672	*MAP kinase-activated protein kinase 2*	-14.30
L12_90101	*MAP kinase-interacting serine/threonine-protein kinase 2*	2.22
L2_19031	*MAP/microtubule affinity-regulating kinase 4*	-1.78
L12_54481	*MAP7 domain-containing protein 1*	-15.24
L1_40761	*MAP7 domain-containing protein 2*	1.78
L12_87384	*MARVEL domain-containing protein 2*	-1.52

L12_89203	*Maspardin*	-2.20
L12_80201	*Mast cell protease 1A*	-1.66
L2_59020	*Mast/stem cell growth factor receptor Kit*	-2.25
L2_67468	*Mast/stem cell growth factor receptor kita*	-4.31
L3_59336	*Matrix Gla protein*	-1.79
L12_76497	*Matrix metalloproteinase-14*	-1.64
L2_52552	*Matrix metalloproteinase-16*	2.25
L12_80075	*Matrix metalloproteinase-23*	-2.40
L1_28456	*Matrix metalloproteinase-28*	1.97
L3_77336	*Matrix metalloproteinase-9*	-2.58
L2_26009	*Max dimerization protein 4*	-2.45
L2_70367	*MCF.2 cell line derived transforming sequence-like 2*	-2.20
L2_72268	*Mediator complex subunit 30*	-1.52
L12_68655	*Mediator of RNA polymerase II transcription subunit 29*	-1.64
L1_62581	*Melanocortin 2 receptor accessory protein*	2.34
L12_76371	*Melanoma inhibitory activity protein 2*	2.32
L2_61948	*Melanoma inhibitory activity protein 3*	-1.71
L2_36511	*Melanopsin*	1.65
L12_83178	*Melanoregulin*	-4.00
L12_89950	*Melanotransferrin*	1.96
L12_89917	*Membrane primary amine oxidase*	2.52
L1_48531	*Membrane progestin receptor alpha-B*	1.69
L12_71788	*Membrane protein FAM174B*	-2.43
L2_51199	*Membrane transport protein XK*	1.65
L2_32562	*Membrane-associated phosphatidylinositol transfer protein 2*	-1.51
L12_67418	*Membrane-associated tyrosine- and threonine-specific cdc2-inhibitory kinase*	1.64
L2_69157	*Meprin A subunit alpha*	3.39
L12_88973	*Meprin A subunit beta*	594.20
L12_90051	*Meprin A subunit beta-like*	-2.50
L3_42310	*Mesencephalic astrocyte-derived neurotrophic factor*	2.77
L12_79805	*Mesenteric estrogen-dependent adipogenesis protein*	-5.04
L12_82795	*Mesoderm-specific transcript homolog protein*	2.17
L3_40537	*Mesoderm-specific transcript protein*	2.22
L12_76133	*Metal regulatory transcription factor 1*	-1.97
L12_58888	*Metallo-beta-lactamase domain-containing protein 1*	-1.72
L12_78811	*Metallo-beta-lactamase domain-containing protein 2*	4.53
L2_34097	*Metallophosphoesterase 1*	-2.16
L12_56310	*Metalloprotease TIKI2*	1.85
L12_87299	*Metalloproteinase inhibitor 2*	-1.67
L12_85914	*Metalloproteinase inhibitor 3*	-2.05
L12_89677	*Metalloreductase STEAP4*	-2.83
L1_6119	*Metastasis suppressor protein 1-like*	2.62
L12_85314	*Methionine adenosyltransferase 2 subunit beta*	1.75

L2_5020	*Methionine sulfoxide reductase A*	1.53
L12_77579	*Methylcytosine dioxygenase TET2*	-1.50
L12_85735	*Methylmalonate-semialdehyde dehydrogenase , mitochondrial*	2.65
L12_31446	*Methylmalonic aciduria and homocystinuria type D homolog, mitochondrial*	2.87
L12_79590	*Methylsterol monooxygenase 1*	2.81
L12_80380	*Methyltransferase-like protein 6*	1.58
L3_70062	*Methyltransferase-like protein 7A*	6.21
L2_46694	*Mevalonate kinase*	1.86
L12_84618	*MFS-type transporter SLC18B1*	4.84
L2_22790	*MHC class I antigen*	-3.43
L1_68211	*MHC class II antigen alpha chain*	-1.96
L12_84635	*MHC class II transactivator*	1.78
L2_65348	*MHC2-interact*	1.76
L1_45554	*MICAL-like protein 1*	-1.84
L2_68011	*Microcephalin*	-1.51
L2_10075	*Microfibril-associated glycoprotein 4-like*	1.92
L12_74633	*Microsomal glutathione S-transferase 3*	1.56
L12_88997	*Microsomal triglyceride transfer protein large subunit*	4.85
L12_78411	*Microtubule-associated protein 1B*	-1.70
L12_72860	*Microtubule-associated protein RP/EB family member 1*	-1.94
L2_20456	*Microtubule-based motor protein*	3.86
L2_7661	*Minor histocompatibility antigen H13*	1.85
L2_54361	*Minor histocompatibility protein HA-1*	-2.01
L12_87635	*Mitochondrial 2-oxodicarboxylate carrier*	1.61
L12_81944	*Mitochondrial 2-oxoglutarate/malate carrier protein*	1.65
L12_87919	*Mitochondrial coenzyme A transporter SLC25A42*	3.10
L12_79888	*Mitochondrial enolase superfamily member 1*	-3.64
L3_63626	*Mitochondrial fission process protein 1*	-1.91
L12_89390	*Mitochondrial glutamate carrier 1*	2.18
L12_82877	*Mitochondrial inner membrane protein OXA1L*	1.56
L12_86907	*Mitochondrial ornithine transporter 1*	6.70
L12_83804	*Mitochondrial peptide methionine sulfoxide reductase*	-1.72
L3_63620	*Mitochondrial pyruvate carrier 2*	-2.13
L12_48795	*Mitochondrial translocator assembly and maintenance protein 41 homolog*	1.55
L12_80996	*Mitochondrial uncoupling protein 2*	8.43
L1_46651	*Mitogen-activated protein kinase 12*	2.20
L1_72890	*Mitogen-activated protein kinase 15*	4.33
L12_81075	*Mitogen-activated protein kinase 15-like*	4.30
L1_4134	*Mitogen-activated protein kinase kinase kinase 13-A*	1.62
L1_31067	*Mitogen-activated protein kinase kinase kinase 5*	2.02
L3_77361	*Mitotic checkpoint serine/threonine-protein kinase BUB1 beta*	1.51

L12_74330	*Mixed lineage kinase domain-like protein-like*	3.30
L3_76582	*MKL/myocardin-like protein 1*	-2.28
L3_72671	*MLN64 N-terminal domain homolog*	-2.04
L12_89091	*MOB kinase activator 1A*	-1.63
L1_64955	*MOB kinase activator 2*	1.58
L1_22462	*Mobilization protein*	1.54
L12_85417	*Moesin*	-1.56
L2_57444	*Molybdenum cofactor sulfurase*	-4.44
L1_24847	*Monoacylglycerol lipase abhd6-B*	3.47
L12_80677	*Monocarboxylate transporter 10*	2.87
L12_85689	*Monocarboxylate transporter 12-B*	-4.34
L12_88761	*Monocarboxylate transporter 3*	-2.10
L1_46437	*Monocarboxylate transporter 6*	3.23
L12_89603	*Monocarboxylate transporter 9*	2.24
L12_82238	*Monocyte to macrophage differentiation factor*	5.36
L3_26683	*MORN repeat-containing protein 2*	-1.55
L2_19554	*MotA/TolQ/ExbB proton channel family protein*	1.98
L1_48439	*Mothers against decapentaplegic homolog 3*	-1.71
L12_66236	*Motor neuron and pancreas homeobox protein 1*	2.68
L1_70956	*Motor neuron and pancreas homeobox protein 1-like*	1.54
L12_89510	*M-phase inducer phosphatase 2*	3.56
L3_82641	*M-protein, striated muscle*	-2.25
L2_37139	*Mu transposase/integrase*	1.72
L12_89177	*Mucin-13*	2.44
L2_55634	*Mucin-5B*	-13.66
L12_87590	*Mucolipin-3*	-2.61
L12_68449	*Mucosa-associated lymphoid tissue lymphoma translocation protein 1*	-1.82
L12_87788	*Mucosa-associated lymphoid tissue lymphoma translocation protein 1 homolog*	-1.66
L1_60757	*Multidrug and toxin extrusion protein 2*	-2.13
L12_86804	*Multidrug resistance protein 1*	1.54
L12_86296	*Multidrug resistance-associated protein 4*	-3.13
L1_22315	*Multidrug resistance-associated protein 4-like*	1.88
L2_63302	*Multidrug resistance-associated protein 9*	-6.80
L12_75739	*Multiple C2 and transmembrane domain-containing protein 2*	-1.95
L1_16496	*Multiple epidermal growth factor-like domains protein 8*	2.73
L1_66575	*Multiple epidermal growth factor-like domains protein 9*	1.84
L2_10899	*Multi-sensor hybrid histidine kinase*	1.53
L1_31129	*Muscarinic acetylcholine receptor M3*	2.07
L12_87310	*Muscle M-line assembly protein unc-89*	-1.81
L1_3730	*Muscleblind-like protein 1-like isoform 10*	-1.50
L1_29195	*MYC associated factor X*	-1.64
L12_81932	*MYC-induced nuclear antigen*	1.59

L12_85248	*Myelin-associated glycoprotein-like*	-1.61
L12_87534	*Myeloid-associated differentiation marker*	-2.32
L12_84177	*Myeloid-associated differentiation marker homolog*	-1.84
L2_63315	*Myeloperoxidase*	-4.59
L1_21941	*Myeloperoxidase-like*	-2.94
L12_45232	*Myocardial zonula adherens protein*	1.85
L3_77699	*Myocardin-like*	-1.62
L12_72935	*Myocardin-related transcription factor A*	-2.86
L2_57634	*Myocyte-specific enhancer factor 2A*	-1.56
L12_87603	*Myopalladin*	-4.16
L2_54914	*Myosin head*	1.84
L2_36168	*Myosin light chain kinase, smooth muscle-like*	1.71
L3_78029	*Myosin regulatory light chain 2, atrial isoform*	7.26
L3_25261	*Myosin regulatory light chain 2, skeletal muscle isoform*	3.92
L3_61812	*Myosin regulatory light chain 2, skeletal muscle isoform type 2*	1.70
L3_58327	*Myosin regulatory light chain 2, ventricular/cardiac muscle isoform*	1.77
L12_86177	*Myosin regulatory light polypeptide 9*	-2.11
L2_31484	*Myosin tail 1*	1.57
L12_88231	*Myosin-11*	-2.91
L2_54097	*Myosin-2 heavy chain*	1.53
L1_60010	*Myosin-IIIb*	2.09
L2_66142	*Myosin-Va-like*	-17.09
L12_80987	*Myosin-VIIa*	2.45
L2_21567	*Myosin-XIX-like*	2.16
L12_88468	*Myotubularin-related protein 10*	-2.06
L1_52203	*Myotubularin-related protein 10-A*	-1.93
L12_77685	*Myotubularin-related protein 11*	-2.10
L1_35735	*Myotubularin-related protein 12*	-1.79
L2_55992	*Myotubularin-related protein 4*	-1.63
L12_61482	*Myotubularin-related protein 6*	-2.01
L12_84756	*Myotubularin-related protein 7*	-1.92
L1_29499	*Myotubularin-related protein 9*	-1.81
L3_74218	*Myozenin-1*	2.12
L12_79045	*N(4)-(beta-N-acetylglucosaminyl)-L-asparaginase*	-2.00
L12_85714	*Na(+)/H(+) exchange regulatory cofactor NHE-RF1*	-7.83
L2_33368	*Na(+)/H(+) exchange regulatory cofactor NHE-RF2-like*	-1.83
L12_83486	*N-acetylgalactosamine kinase*	-7.47
L3_57466	*N-acetylgalactosamine-6-sulfatase*	-2.30
L12_75606	*N-acetylglucosamine-1-phosphotransferase subunit gamma*	-2.34
L2_17167	*N-acetylglucosamine-1-phosphotransferase subunits alpha/beta*	-1.71
L12_77804	*N-acetylglucosamine-6-sulfatase*	-2.35

L12_86289	*N-acetylmuramoyl-L-alanine amidase*	5.57
L12_81362	*N-acetylneuraminate lyase*	-2.46
L12_72496	*NACHT, LRR and PYD domains-containing protein 1*	-1.60
L2_43571	*N-acylglucosamine 2-epimerase*	-8.94
L2_11813	*N-acylglucosamine 2-epimerase-like*	-5.89
L1_42894	*NAD kinase-like*	2.75
L3_84761	*NAD(P)H-hydrate epimerase*	1.61
L2_53767	*NAD-dependent protein deacetylase sirtuin-7*	-1.53
L3_33682	*NADH dehydrogenase 1 alpha subcomplex subunit 4*	1.56
L12_59462	*NADH dehydrogenase 1 beta subcomplex subunit 9*	1.53
L12_83288	*NADP-dependent malic enzyme*	12.79
L12_87230	*NADP-dependent malic enzyme, mitochondrial*	1.53
L12_80563	*NADPH:adrenodoxin oxidoreductase, mitochondrial*	1.70
L12_45995	*NADPH-dependent diflavin oxidoreductase 1*	-1.55
L3_59912	*Nanos homolog 1*	-1.55
L12_84945	*N-arachidonyl glycine receptor*	-1.68
L3_67635	*Nattectin*	-6.25
L12_88270	*Natterin-3-like*	34873.18
L12_80640	*Natural resistance-associated macrophage protein 2*	2.38
L2_45009	*NCK-interacting protein with SH3 domain*	-1.81
L12_86027	*NEDD8-activating enzyme E1 regulatory subunit*	-1.61
L1_36000	*Neogenin*	2.14
L1_44137	*Nephrosin*	7.56
L12_87673	*Neprilysin*	2.84
L2_57911	*Nesprin-2-like*	-2.91
L2_27640	*Netrin-1*	-1.89
L12_88713	*Neural cell adhesion molecule 1*	2.78
L12_46638	*Neural Wiskott-Aldrich syndrome protein-like*	-1.55
L2_46214	*Neuralized-like protein 1A*	-1.78
L2_28273	*Neurexin-3a-alpha*	3.88
L1_22991	*Neurobeachin*	-1.78
L2_55562	*Neurobeachin-like protein 1*	-1.75
L2_40075	*Neuroblast differentiation-associated protein AHNAK-like*	-2.39
L1_23033	*Neurocalcin-delta B*	-2.01
L1_66444	*Neuroendocrine convertase 1*	2.62
L1_25658	*Neuroendocrine convertase 2*	2.56
L12_56885	*Neuroendocrine protein 7B2*	2.00
L1_14855	*Neurogenic differentiation factor 1*	1.86
L12_73028	*Neuroguidin*	1.93
L1_44416	*Neuromedin-B-like*	311.30
L1_48780	*Neuromedin-U receptor 1*	3.90
L1_6275	*Neuromedin-U receptor 1-like*	8.24
L1_55875	*Neuron navigator 2*	-1.74
L2_33171	*Neuronal membrane glycoprotein M6-b*	-1.63
L2_58058	*Neuronal migration protein doublecortin*	-2.53

L1_45034	*Neuronal PAS domain-containing protein 4*	1.74
L2_47451	*Neuronal tyrosine-phosphorylated phosphoinositide-3-kinase adapter 1*	4.61
L1_53262	*Neuropathy target esterase*	2.17
L12_87891	*Neuropeptide Y receptor type 1*	-2.17
L1_26611	*Neuropeptides B/W receptor type 1*	2.19
L1_15960	*Neuropeptides B/W receptor type 2*	2.43
L1_38927	*Neurotrypsin*	13.55
L12_88162	*Neutral ceramidase*	201.39
L12_80306	*Neutral cholesterol ester hydrolase 1*	-1.98
L3_75782	*Neutrophil cytosol factor 1*	-2.88
L12_86886	*Neutrophil cytosol factor 2*	-1.67
L1_70128	*NF-kappa-B inhibitor delta*	2.73
L2_55474	*NF-kappa-B inhibitor delta-like*	-1.63
L2_44833	*Niban-like protein 1*	2.01
L2_64373	*Nicastrin*	-2.08
L2_43569	*Nickel-cobalt-cadmium resistance protein NccA*	1.79
L1_34186	*Nicotinate phosphoribosyltransferase*	2.56
L12_87273	*Nidogen-1*	-1.76
L2_75791	*Niemann-Pick C1 protein*	-3.81
L12_89172	*Niemann-Pick C1-like protein 1*	4.42
L12_72895	*NILT4 leukocyte receptor*	-2.39
L12_75748	*Ninjurin-1*	-34.42
L2_44259	*NK-tumor recognition protein*	-1.76
L1_54817	*N-lysine methyltransferase SETD8-A*	-1.55
L3_69516	*N-lysine methyltransferase SMYD2-B*	1.61
L12_84742	*Noelin*	-1.65
L1_62070	*Noggin*	2.04
L12_79561	*Non-lysosomal glucosylceramidase*	2.84
L12_87137	*Non-specific lipid-transfer protein*	4.39
L12_56979	*Non-syndromic hearing impairment protein 5-like*	-1.87
L12_89949	*Nostrin*	-2.30
L3_53517	*Notchless protein homolog 1*	1.52
L4_71417	*Novel immune-type receptor 1*	-2.13
L4_72220	*Novel immune-type receptor 17*	-1.87
L4_71418	*Novel immune-type receptor 2*	-1.91
L4_80714	*Novel immune-type receptor 20*	-1.74
L4_71804	*Novel immune-type receptor 22*	-1.94
L4_82340	*Novel immune-type receptor 24*	-4.15
L4_73588	*Novel immune-type receptor 25*	-1.76
L4_71296	*Novel immune-type receptor 26*	-1.96
L4_71502	*Novel immune-type receptor 3*	-1.69
L4_72098	*Novel immune-type receptor 4*	-1.65
L4_71928	*Novel immune-type receptor 5*	-1.57

L2_3363	*Novel protein containing an Acyl-CoA dehydrogenase, N-terminal and C-terminal domain*	1.53
L1_53099	*Novel protein similar to human plakophilin 2 (PKP2)*	-1.81
L1_42265	*Novel protein similar to non-vertebrate chitin synthase protein*	1.59
L1_11666	*Novel protein similar to vertebrate xanthine dehydrogenase (XDH)*	1.79
L3_79545	*N-terminal EF-hand calcium-binding protein 1*	1.95
L1_8205	*N-terminal ig-like domain of cellulase family protein*	4.49
L2_67535	*NUAK family SNF1-like kinase 1*	-2.10
L12_88652	*NUAK family SNF1-like kinase 2*	-2.60
L3_75482	*Nuclear autoantigenic sperm protein*	3.74
L12_82331	*Nuclear factor 1 X-type*	1.60
L12_74319	*Nuclear factor of activated T-cells 5-like*	-1.64
L12_85392	*Nuclear factor of activated T-cells, cytoplasmic 2*	-2.21
L12_84049	*Nuclear factor of activated T-cells, cytoplasmic 3*	4.99
L1_28904	*Nuclear factor of activated T-cells, cytoplasmic 3-like*	2.33
L2_44556	*Nuclear factor of activated T-cells, cytoplasmic 4*	1.78
L12_89587	*Nuclear GTPase SLIP-GC*	5.69
L12_79910	*Nuclear migration protein nudC*	-1.51
L3_78423	*Nuclear pore complex protein Nup153-like*	1.59
L1_52051	*Nuclear pore membrane glycoprotein 210-like*	2.36
L12_84589	*Nuclear protein 1-like*	4.60
L12_79345	*Nuclear receptor corepressor 2*	-1.69
L2_24422	*Nuclear receptor ROR-alpha-like*	-2.49
L1_43315	*Nuclear receptor ROR-beta*	1.98
L1_61104	*Nuclear receptor ROR-gamma*	-2.30
L12_88831	*Nuclear receptor subfamily 1 group D member 2*	1.57
L1_57560	*Nuclear receptor subfamily 2 group C member 2*	5.95
L12_87095	*Nuclear receptor subfamily 5 group A member 2*	3.07
L12_43934	*Nuclease-sensitive element-binding protein 1*	1.52
L1_38654	*Nucleolar complex protein 3 homolog*	1.51
L1_56326	*Nucleoporin NUP188 homolog*	1.57
L12_48422	*Nucleoredoxin*	-2.22
L12_86004	*Nucleoside diphosphate kinase*	-10.80
L3_46180	*Nucleoside diphosphate kinase 3*	-1.61
L12_89819	*Nucleoside diphosphate kinase 7*	1.86
L3_43406	*Nucleoside diphosphate kinase B*	2.58
L12_74027	*Nucleoside diphosphate-linked moiety X motif 19, mitochondrial*	-2.06
L2_69420	*Nucleotide-binding oligomerization domain-containing protein 2*	-2.35
L12_63630	*OCIA domain-containing protein 2*	2.64
L2_48930	*Octopamine receptor beta-3R*	1.66
L2_18532	*Oligopeptide transporter, OPT family*	1.64

L12_80213	*Oligophrenin-1*	-1.84
L3_77777	*Oocyte zinc finger protein XlCOF20*	-1.62
L12_87687	*Opioid growth factor receptor*	-1.80
L12_48513	*Origin recognition complex subunit 2*	-3.15
L12_79592	*ORM1-like protein 1*	2.63
L3_38303	*ORM1-like protein 2*	-2.09
L12_85232	*Ornithine carbamoyltransferase, mitochondrial*	-1.99
L12_79486	*Osmotic stress transcription factor 1*	2.61
L4_41684	*Osteocalcin*	1.52
L12_86980	*Osteopetrosis-associated transmembrane protein 1*	-1.88
L12_86528	*Otopetrin-2*	3.50
L1_21048	*Otopetrin-3*	6.43
L12_79155	*Ovarian cancer-associated gene 2 protein homolog*	1.53
L1_13695	*Ovochymase-2-like*	9.55
L3_79813	*OX-2 membrane glycoprotein-like*	-1.59
L2_45919	*Oxidized low-density lipoprotein receptor 1-like*	-1.51
L12_61555	*Oxysterol-binding protein*	2.24
L3_71081	*Oxysterol-binding protein 2*	-2.15
L12_86517	*Oxysterol-binding protein-related protein 11*	3.03
L2_35775	*Oxysterol-binding protein-related protein 6*	-1.50
L12_81260	*Oxysterols receptor LXR-alpha-like*	1.52
L2_73132	*P2X purinoceptor 5*	1.51
L12_83324	*P2X purinoceptor 7*	-1.64
L3_79141	*P2Y purinoceptor 1*	-2.07
L1_67169	*P2Y purinoceptor 1-like*	-3.92
L1_68371	*P2Y purinoceptor 8*	-3.59
L12_46879	*P53 and DNA damage-regulated protein 1*	1.89
L1_41759	*Paired box protein Pax-4*	3.83
L2_59193	*Paired box protein Pax-5*	-3.29
L2_24557	*Paired box protein Pax-6*	1.51
L12_48049	*Paladin*	-3.16
L12_83643	*Palmitoyl-protein thioesterase 1*	-1.54
L2_65364	*Palmitoyltransferase ZDHHC17*	-1.66
L1_39193	*Pancreas/duodenum homeobox protein 1-like*	1.59
L12_83259	*Pancreatic alpha-amylase*	76.08
L12_80935	*Pancreatic lipase-related protein 1*	80.10
L2_68561	*Pannexin-1*	-1.66
L12_81994	*Pantothenate kinase 1*	-2.01
L2_1593	*Par-3 family cell polarity regulator*	-5.54
L3_80205	*Patatin-like phospholipase domain-containing protein 2*	3.24
L12_71681	*Patatin-like phospholipase domain-containing protein 7*	1.75
L12_89048	*Patched domain-containing protein 3*	-105.85
L2_68637	*PDEase I*	2.01
L12_87659	*PDZ and LIM domain protein 1*	-1.65
L12_87062	*PDZ and LIM domain protein 2*	5.16

L12_86682	*PDZ and LIM domain protein 3*	-2.06
L12_84310	*PDZ and LIM domain protein 5*	-1.89
L1_42593	*PDZ and LIM domain protein 7*	-1.87
L2_70218	*PDZ domain-containing protein 2*	-1.52
L2_36436	*PDZ domain-containing protein GIPC3*	2.60
L3_83339	*Peflin*	1.53
L1_51528	*Pendrin-like*	-1.78
L12_87775	*Pepsin A*	2556.36
L2_17471	*Peptidase (Caspase-1 like protein)*	1.81
L2_29828	*Peptidase S10*	-1.65
L2_48481	*Peptidase S41*	1.57
L1_18068	*Peptide deformylase*	2.21
L4_39425	*Peptide Y*	3983.42
L1_19050	*Peptidyl-prolyl cis-trans isomerase FKBP4-like*	1.52
L2_55189	*Peptidyl-prolyl cis-trans isomerase FKBP9*	-1.78
L12_39430	*Perforin*	17.04
L12_72038	*Perforin-1*	-1.94
L2_40342	*Perforin-1-like*	1.94
L12_86385	*Perilipin-3*	3.83
L12_87340	*Periodic tryptophan protein 1 homolog*	-1.61
L1_57055	*Periostin*	-1.96
L3_69904	*Peripheral myelin protein 22*	-2.53
L12_87488	*Periplakin*	9.19
L3_72270	*Peroxiredoxin-1*	1.54
L3_49822	*Peroxiredoxin-6*	-1.57
L12_82594	*Peroxisomal 2,4-dienoyl-CoA reductase*	2.30
L12_88739	*Peroxisomal acyl-coenzyme A oxidase 1*	-1.59
L12_80191	*Peroxisomal bifunctional enzyme*	2.27
L12_86031	*Peroxisomal membrane protein 11C*	1.89
L12_88832	*Peroxisomal membrane protein PMP34*	-1.58
L12_74763	*Peroxisomal multifunctional enzyme type 2*	1.71
L1_2415	*Peroxisomal sarcosine oxidase*	7.06
L1_58994	*Peroxisome proliferator-activated receptor gamma*	2.71
L1_32280	*Peroxisome proliferator-activated receptor gamma coactivator 1-alpha*	2.02
L12_84594	*PH and SEC7 domain-containing protein 3*	-1.92
L2_80438	*PH domain leucine-rich repeat-containing protein phosphatase 2*	2.04
L2_40092	*PHD finger protein 19*	-1.53
L1_14447	*Phenylalanine-tRNA ligase beta subunit*	1.52
L12_78667	*-phosphatase 2, mitochondrial*	-1.74
L12_82471	*Phosphatidate cytidylyltransferase 1*	2.88
L1_60846	*Phosphatidate phosphatase LPIN1*	-12.40
L12_83008	*Phosphatidylcholine transfer protein*	-5.11
L12_58050	*Phosphatidylethanolamine-binding protein 1*	1.66

L12_86583	*Phosphatidylinositol 3,4,5-trisphosphate 3-phosphatase TPTE2*	-50.50
L12_76344	*Phosphatidylinositol 3,4,5-trisphosphate 5-phosphatase 1*	-1.67
L12_83970	*Phosphatidylinositol 3,4,5-trisphosphate-dependent Rac exchanger 1 protein*	-2.25
L2_60690	*Phosphatidylinositol 3-kinase regulatory subunit gamma*	-1.62
L12_44319	*Phosphatidylinositol 4,5-bisphosphate 3-kinase catalytic subunit delta isoform*	-1.91
L12_81627	*Phosphatidylinositol 4-kinase alpha*	-1.53
L12_87945	*Phosphatidylinositol 4-kinase type 2-beta*	2.97
L12_89313	*Phosphatidylinositol 4-phosphate 3-kinase C2 domain-containing subunit gamma*	2.19
L12_87336	*Phosphatidylinositol transfer protein alpha isoform*	-2.23
L1_38640	*Phosphatidylinositol-4-phosphate 3-kinase C2 domain-containing subunit gamma-like*	2.13
L12_84441	*Phosphatidylinositol-glycan biosynthesis class W protein*	-2.14
L2_18408	*Phosphatidylinositol-glycan biosynthesis class X protein-like*	-1.62
L3_19985	*Phosphatidylserine decarboxylase proenzyme*	2.88
L12_88934	*Phosphatidylserine synthase 1*	-1.63
L12_85325	*Phospho p8*	4.64
L1_82669	*Phosphoethanolamine N-methyltransferase 3*	535.96
L2_70422	*Phosphoethanolamine N-methyltransferase 3-like*	2.32
L12_88393	*Phosphoglucomutase-1*	2.35
L12_85361	*Phosphoglucomutase-like protein 5*	-2.92
L1_58233	*Phosphoglycerate kinase*	2.11
L12_87587	*Phosphoinositide 3-kinase adapter protein 1*	-2.18
L12_75503	*Phosphoinositide 3-kinase regulatory subunit 5*	-1.62
L12_76680	*Phospholamban*	-2.06
L3_34732	*Phospholipase A1 member A*	1.64
L1_22234	*Phospholipase A2*	32.91
L4_24295	*Phospholipase A2, major isoenzyme*	22.35
L12_88630	*Phospholipase B1, membrane-associated*	23.06
L1_56145	*Phospholipase B1, membrane-associated-like*	4.17
L12_79510	*Phospholipase D1*	2.82
L12_85209	*Phospholipase D4*	-1.62
L2_54605	*Phospholipase DDHD1*	-1.64
L3_72561	*Phospholipid hydroperoxide glutathione peroxidase, mitochondrial*	1.99
L12_86332	*Phospholysine phosphohistidine inorganic pyrophosphate phosphatase*	1.76
L12_87053	*Phosphomannomutase 1*	1.82
L12_73914	*Phosphomevalonate kinase*	1.74
L3_36790	*Phosphoprotein associated with glycosphingolipid-enriched microdomains 1-like*	-1.79

L12_72017	*Phosphoribosyl pyrophosphate synthase-associated protein 2*	-1.63
L2_29368	*Phosphoribosyl-AMP cyclohydrolase*	1.88
L1_45298	*Phosphoribosylformylglycinamidine synthase 2*	1.94
L3_79063	*Phosphoribosyltransferase domain-containing protein 1*	3.51
L12_75746	*Phytanoyl-CoA dioxygenase domain-containing protein 1*	-1.50
L12_85502	*Phytanoyl-CoA dioxygenase, peroxisomal*	1.55
L2_27072	*Piezo-type mechanosensitive ion channel component 2*	2.74
L12_89689	*PiggyBac transposable element-derived protein 3*	3.81
L1_62968	*PiggyBac transposable element-derived protein 5*	-1.60
L1_54500	*Pigment epithelium-derived factor*	1.67
L12_73640	*PIH1 domain-containing protein 1*	1.82
L12_76780	*PI-PLC X domain-containing protein 1*	-2.33
L12_83572	*Pirin*	1.51
L1_30072	*Piscidin-5*	5.71
L12_75834	*Pituitary homeobox 1*	8.90
L12_75446	*Pituitary tumor-transforming gene 1 protein-interacting protein*	-30.40
L1_10773	*Piwi-like protein 2*	3.10
L12_85554	*PLAC8-like protein 1*	2.63
L12_32663	*Placenta-specific gene 8 protein*	-13.50
L12_89076	*Plakophilin-1*	3.48
L12_87288	*Plakophilin-2*	-1.95
L12_87840	*Plakophilin-3*	2.30
L1_29736	*Plakophilin-3-like*	2.37
L12_84077	*Plasma kallikrein*	1.72
L12_48044	*Plasma membrane calcium-transporting ATPase 2*	-1.75
L2_30140	*Plasma membrane calcium-transporting ATPase 3*	-1.73
L1_26123	*Plasma membrane calcium-transporting ATPase 4*	-1.80
L2_57494	*Plasma serine protease inhibitor*	-1.51
L3_65700	*Plasminogen*	119.13
L12_81177	*Plasmolipin*	-1.96
L12_75431	*Plasticin*	-1.64
L12_89432	*Plastin-1*	1.53
L12_85030	*Plastin-2*	-1.53
L12_86214	*Platelet glycoprotein 4*	451.42
L1_29203	*Platelet receptor Gi24-like*	-1.58
L1_12275	*Platelet-derived growth factor receptor-like protein-like*	3.78
L3_80427	*Pleckstrin*	-2.03
L12_81879	*Pleckstrin homology domain-containing family A member 2*	-1.88
L12_76828	*Pleckstrin homology domain-containing family A member 3*	-2.03
L12_87713	*Pleckstrin homology domain-containing family G member 4B*	-2.45

L12_84012	*Pleckstrin homology domain-containing family G member 7*	8.80
L12_85908	*Pleckstrin homology domain-containing family J member 1*	-3.18
L12_84895	*Pleckstrin homology domain-containing family M member 1*	-1.70
L12_76018	*Pleckstrin homology domain-containing family O member 2*	-1.64
L1_31437	*Pleckstrin homology-like domain family B member 1*	3.39
L2_47909	*Plectin*	5.17
L2_62028	*Plexin domain-containing protein 1*	-1.61
L2_62798	*Plexin domain-containing protein 2*	-2.23
L2_56532	*Plexin-B1*	1.60
L12_87716	*Plexin-C1*	-1.55
L2_32927	*Plexin-C1-like*	-2.15
L12_81797	*PML-RARA-regulated adapter molecule 1*	-2.11
L12_40213	*PMS1 protein homolog 1*	2.29
L2_54540	*Pol polyprotein*	-4.60
L12_85722	*Poliovirus receptor-related protein 1-like*	1.64
L1_52818	*Poliovirus receptor-related protein 3-like*	-1.80
L12_81775	*Poliovirus receptor-related protein 4-like*	-1.55
L1_56907	*Pol-like protein*	-2.02
L12_79370	*Poly*	-1.80
L1_42481	*Poly(A) RNA polymerase GLD2*	-1.73
L12_79029	*Poly(U)-specific endoribonuclease*	125.42
L12_81511	*Poly(U)-specific endoribonuclease-C*	-1.51
L3_79337	*Polyadenylate-binding protein 1-A*	1.64
L2_15719	*Polyadenylate-binding protein-interacting protein 2*	-2.77
L3_73486	*Polycomb group RING finger protein 5*	1.72
L12_85500	*Polycomb group RING finger protein 6*	3.14
L12_86275	*Polycomb protein eed*	-5.89
L2_64470	*Polycystic kidney disease protein 1-like 1-like*	-1.61
L12_85034	*Polymerase I and transcript release factor*	-1.91
L1_40062	*Polypeptide N-acetylgalactosaminyltransferase 6-like*	2.89
L3_73804	*Polyubiquitin-C*	-1.58
L1_65263	*Popeye domain-containing protein 3*	2.47
L2_26375	*Potassium channel subfamily K member 16*	2.26
L1_58026	*Potassium channel subfamily K member 18-like*	-2.16
L2_44387	*Potassium channel subfamily K member 4-like*	-6.98
L12_88394	*Potassium channel subfamily K member 5-like*	-1.86
L12_85743	*Potassium channel subfamily K member 6*	7.24
L1_47780	*Potassium channel subfamily T member 1*	-1.71
L12_44128	*Potassium voltage-gated channel subfamily E member 2*	4.34
L3_73084	*Potassium voltage-gated channel subfamily G member 3*	-1.62
L3_79208	*Potassium voltage-gated channel subfamily H member 5*	2.65
L2_33646	*Potassium voltage-gated channel subfamily S member 2*	2.33

L1_65160	*POTE ankyrin domain family member J*	1.57
L12_83131	*PQ-loop repeat-containing protein 1*	-1.71
L12_65889	*PQ-loop repeat-containing protein 3*	-1.73
L2_37157	*PR domain zinc finger protein 1*	-1.61
L1_31973	*PR domain zinc finger protein 16-like*	7.54
L2_27369	*Pre-B-cell leukemia transcription factor 1-like*	1.96
L3_26459	*Pregnancy-associated glycoprotein*	16.43
L1_57389	*Prenylcysteine oxidase-like*	-1.54
L2_82924	*Prepronociceptin-like*	-3.23
L2_28773	*Prestin*	-1.53
L1_39976	*Prickle-like protein 1*	-2.46
L12_88239	*Proactivator polypeptide*	-1.56
L1_51254	*Probable aconitate hydratase, mitochondrial*	-2.54
L2_37748	*Probable alpha-ketoglutarate-dependent dioxygenase ABH5-like*	-1.60
L1_48586	*Probable aminoglycoside efflux pump*	1.50
L1_51766	*Probable ATP-dependent RNA helicase DDX11*	2.07
L12_67155	*Probable ATP-dependent RNA helicase DDX28*	-1.88
L1_63146	*Probable ATP-dependent RNA helicase DDX31*	2.09
L2_60133	*Probable ATP-dependent RNA helicase DHX40*	-1.54
L1_62646	*Probable beta-galactosidase B*	1.70
L1_61892	*Probable cation-transporting ATPase 13A2*	1.65
L12_89991	*Probable cation-transporting ATPase 13A3*	2.61
L12_89103	*Probable cysteine-tRNA ligase, mitochondrial*	2.10
L1_39760	*Probable D-xylulose kinase A*	1.56
L12_84302	*Probable E3 ubiquitin-protein ligase RNF217*	-2.25
L3_35886	*Probable flavin-containing monoamine oxidase A*	1.67
L12_65414	*Probable gluconokinase*	-2.09
L12_74056	*Probable glucuronokinase 2*	2.06
L12_72970	*Probable glutathione peroxidase 8*	-1.55
L12_89893	*Probable G-protein coupled receptor 112*	3.15
L12_80970	*Probable G-protein coupled receptor 113*	1.63
L12_73074	*Probable G-protein coupled receptor 139-like*	1.63
L12_81210	*Probable G-protein coupled receptor 141-like*	-2.12
L2_36089	*Probable G-protein coupled receptor 153*	-1.68
L12_87779	*Probable G-protein coupled receptor 157*	1.68
L12_84252	*Probable G-protein coupled receptor 174*	-2.00
L1_45316	*Probable G-protein coupled receptor 25*	-2.78
L12_85545	*Probable G-protein coupled receptor 34*	-1.70
L2_48674	*Probable G-protein coupled receptor 63*	-1.71
L1_39527	*Probable G-protein coupled receptor 97*	1.58
L1_16331	*Probable guanine nucleotide exchange factor MCF2L2*	-1.78
L12_65314	*Probable isoprenylcysteine alpha-carbonyl methylesterase ICMEL2*	-2.85
L12_88462	*Probable low affinity copper uptake protein 2*	-8.58

L1_70395	*Probable methylmalonate-semialdehyde dehydrogenase , mitochondrial*	2.30
L1_19906	*Probable methylmalonyl-CoA mutase, mitochondrial*	1.59
L1_36554	*Probable methyltransferase-like protein 15*	-2.06
L2_7737	*Probable oxidoreductase YoaE*	7.21
L3_57463	*Probable palmitoyltransferase ZDHHC16*	1.85
L1_64311	*Probable palmitoyltransferase ZDHHC8*	-2.13
L1_33389	*Probable phosphatase phospho1*	2.33
L1_60446	*Probable phospholipid-transporting ATPase 11C*	-1.66
L12_88679	*Probable phospholipid-transporting ATPase IB*	1.98
L1_52557	*Probable phospholipid-transporting ATPase IF*	-1.67
L2_70250	*Probable phospholipid-transporting ATPase IG*	-1.73
L2_46756	*Probable phospholipid-transporting ATPase IH*	-2.93
L12_84959	*Probable phospholipid-transporting ATPase IIB*	-2.30
L12_80173	*Probable phospholipid-transporting ATPase IM*	-2.57
L2_49107	*Probable phosphoserine aminotransferase*	-1.50
L12_86606	*Probable polypeptide N-acetylgalactosaminyltransferase 8*	11.83
L1_64335	*Probable polypeptide N-acetylgalactosaminyltransferase 8-like*	10.61
L3_79721	*Probable protein BRICK1*	1.69
L12_86107	*Probable serine carboxypeptidase CPVL*	-8.90
L3_24151	*Probable serine carboxypeptidase CPVL-like*	-2.60
L1_18085	*Probable serine/threonine-protein kinase kinX-like*	-14.61
L12_70501	*Probable sodium-coupled neutral amino acid transporter 6*	-4.03
L12_89119	*Probable threonine-tRNA ligase 2, cytoplasmic*	1.66
L3_75092	*Pro-cathepsin H*	-2.26
L12_82582	*Procollagen galactosyltransferase 2*	-2.91
L12_77947	*Proenkephalin-A*	3.27
L12_50260	*Profilin-1*	-1.70
L2_72060	*Progestin and adipoQ receptor family member 3*	2.61
L12_66509	*Progestin and adipoQ receptor family member 4*	-2.62
L2_14964	*Programmed cell death protein 4-like*	1.93
L12_81229	*Pro-interleukin-16*	-2.08
L12_88368	*Prolactin regulatory element-binding protein*	1.52
L12_87502	*Proliferation-associated protein 2G4*	1.80
L12_88586	*Proline dehydrogenase 1, mitochondrial*	-15.08
L1_34424	*Proline-rich membrane anchor 1-like, partial*	-2.38
L12_72592	*Proline-rich nuclear receptor coactivator 1-like*	-1.56
L12_77236	*Proline-rich transmembrane protein 4-like*	-3.08
L12_64629	*Proline-serine-threonine phosphatase-interacting protein 1*	-2.66
L12_87231	*Proline-serine-threonine phosphatase-interacting protein 2*	-2.25
L1_36571	*Prolyl 3-hydroxylase 1*	2.98
L3_8463	*Pro-melanin-concentrating hormone*	2.23

L12_72493	*Promethin*	-2.20
L12_83044	*Prominin-1*	-14.03
L3_64210	*Pro-neuregulin-1, membrane-bound isoform*	-1.71
L12_57274	*Pro-neuregulin-2, membrane-bound isoform-like*	-1.60
L4_40445	*Pro-neuropeptide Y*	5.19
L12_89026	*Propionyl-CoA carboxylase alpha chain, mitochondrial*	-2.34
L2_82793	*Prostacyclin synthase*	-24.57
L12_81626	*Prostaglandin E synthase*	-1.54
L12_87631	*Prostaglandin E2 receptor EP3 subtype*	-2.50
L12_87728	*Prostaglandin G/H synthase 1*	-1.60
L2_40596	*Prostate-associated microseminoprotein*	-2.38
L1_51350	*Protachykinin-like*	3.68
L2_63970	*Proteasome subunit alpha type*	-1.76
L12_78206	*Protein AMBP*	3.29
L12_89217	*Protein amnionless*	-1781.91
L12_52218	*Protein argonaute-1*	1.76
L12_81550	*Protein atonal homolog 8*	-1.58
L3_80057	*Protein BCCIP homolog*	1.50
L2_55544	*Protein bicaudal C homolog 1*	-11.37
L2_33080	*Protein bicaudal C homolog 1-B*	-2.09
L1_56161	*Protein broad-minded*	2.93
L12_62950	*Protein C21orf2*	-2.38
L2_17039	*Protein CASP*	-1.56
L12_44222	*Protein ccsmst1*	1.61
L1_47219	*Protein C-ets-1*	-2.13
L2_44742	*Protein C-ets-2*	-2.23
L3_42483	*Protein chibby homolog 1*	-1.60
L12_88721	*Protein cordon-bleu-like*	1.55
L2_12269	*Protein CYR61-like*	1.72
L1_25617	*Protein Daple-like*	-1.64
L12_82694	*Protein DPCD*	-1.54
L12_82565	*Protein dpy-19 homolog 4*	-2.18
L12_86661	*Protein ERGIC-53*	4.06
L2_31996	*Protein eva-1 homolog A*	2.31
L12_79104	*Protein eva-1 homolog C*	5.04
L2_8956	*Protein FAM105B*	2.00
L2_73794	*Protein FAM107A*	-22.75
L3_59340	*Protein FAM107B*	-1.58
L1_39270	*Protein FAM110C*	5.69
L1_36673	*Protein FAM110C-like*	2.88
L12_89106	*Protein FAM114A2*	1.87
L2_56282	*Protein FAM132B-like*	-2.02
L3_55229	*Protein FAM136A*	1.60
L2_59203	*Protein FAM13C*	1.86
L12_88335	*Protein FAM151A*	356.85

L12_81966	*Protein FAM151B*	-1.82
L1_18445	*Protein FAM166B*	1.70
L2_55065	*Protein FAM179B*	1.53
L1_58812	*Protein FAM188B*	-1.59
L2_50237	*Protein FAM18A*	-1.56
L1_48435	*Protein FAM196B*	-2.64
L3_76300	*Protein FAM19A2*	2.06
L12_81227	*Protein FAM210B*	1.59
L3_50774	*Protein FAM43A*	-1.97
L1_44355	*Protein FAM45A*	-3.23
L1_56227	*Protein FAM46C*	-1.63
L2_52628	*Protein FAM54B*	-1.69
L12_88271	*Protein FAM69A*	1.72
L2_75717	*Protein FAM69B*	-4.83
L2_58011	*Protein FAM70B*	-2.31
L2_67790	*Protein FAM78A*	-1.95
L12_72590	*Protein FAM83F-like*	-5.78
L12_76849	*Protein FAM83H*	2.47
L1_27415	*Protein FAM83H-like*	2.42
L1_66516	*Protein FAM84A*	3.61
L12_72474	*Protein FAM91A1*	-2.38
L12_87090	*Protein farnesyltransferase subunit beta*	-1.92
L12_84257	*Protein FEV*	1.78
L2_62803	*Protein furry homolog*	-2.53
L2_25228	*Protein HflK*	2.33
L2_40497	*Protein HID1*	1.69
L1_20112	*Protein Hikeshi*	2.45
L12_76047	*Protein KIAA0317*	1.82
L1_21117	*Protein KIBRA*	-2.41
L1_72888	*Protein kinase C and casein kinase substrate in neurons protein 1*	4.17
L12_72663	*Protein kinase C beta type*	-2.55
L12_88587	*Protein kinase C eta type*	-2.01
L2_58426	*Protein kinase C-like 2*	-2.78
L3_80161	*Protein lin-7 homolog B*	-1.53
L12_85037	*Protein LTV1 homolog*	1.61
L2_32254	*Protein lunapark-A*	1.59
L12_80135	*Protein lyl-1*	-3.03
L1_35081	*Protein MAATS1*	1.89
L1_27179	*Protein mab-21*	-1.56
L12_78874	*Protein mab-21-like 2*	-2.13
L12_86403	*Protein MAL2*	19.13
L12_76496	*Protein MEF2BNB homolog*	-1.74
L2_57815	*Protein Mpv17*	-1.50
L1_54017	*Protein naked cuticle homolog 2*	-1.67

L12_89130	*Protein NDRG1*	5.42
L12_74407	*Protein Niban*	-1.64
L3_47297	*Protein NipSnap homolog 3B*	1.74
L12_86885	*Protein NLRC3*	-1.73
L2_59761	*Protein notum homolog*	-7.95
L12_87552	*Protein N-terminal asparagine amidohydrolase*	-2.39
L12_81962	*Protein numb homolog*	-1.61
L12_78219	*Protein odd-skipped-related 2*	14.37
L12_74216	*Protein odd-skipped-related 2-A*	1.64
L12_80383	*Protein odr-4 homolog*	1.50
L12_84153	*Protein pelota homolog*	1.76
L12_85399	*Protein PET100 homolog, mitochondrial*	-1.72
L12_84475	*Protein phosphatase 1 regulatory subunit 12B*	-1.82
L1_26253	*Protein phosphatase 1 regulatory subunit 14A*	-2.03
L1_17567	*Protein phosphatase 1 regulatory subunit 1B-like*	1.71
L12_89633	*Protein phosphatase 1 regulatory subunit 21*	-1.78
L12_87413	*Protein phosphatase 1 regulatory subunit 3C-B*	9.00
L3_77700	*Protein phosphatase 1, regulatory (inhibitor) subunit 14Ab*	2.47
L1_32522	*Protein phosphatase 1L*	1.55
L12_82360	*Protein phosphatase Slingshot homolog 2*	-2.04
L1_29647	*Protein phosphatase Slingshot homolog 2-like*	-2.09
L1_58228	*Protein POF1B*	2.52
L12_48608	*Protein QNR-71*	1.67
L12_81027	*Protein quaking-A*	-1.52
L12_35444	*Protein rogdi homolog*	-5.27
L3_30385	*Protein S100-A13*	1.72
L12_48239	*Protein S100-A1-like*	1.62
L3_26679	*Protein S100-P*	10.76
L2_45206	*Protein sel-1 homolog 3*	-1.57
L2_8593	*Protein sel-1 homolog 3-like*	-1.60
L12_83011	*Protein SFI1 homolog isoform 2*	-1.51
L2_44557	*Protein SGT1 homolog*	1.68
L12_87802	*Protein Shroom2*	-1.51
L12_50177	*Protein spinster homolog 1*	-1.52
L2_43228	*Protein spire homolog 1*	3.10
L1_81903	*Protein spire homolog 2-like*	68.28
L12_86242	*Protein SSUH2 homolog*	-2.31
L1_39964	*Protein TFG*	2.38
L3_75050	*Protein TMED8*	-1.56
L2_49671	*Protein Tob2*	-14.76
L1_44787	*Protein transport protein Sec16B-like*	4.81
L12_86406	*Protein transport protein Sec23A*	2.72
L12_87585	*Protein transport protein Sec23B*	2.34
L12_75786	*Protein transport protein Sec24A*	2.35
L12_88999	*Protein transport protein Sec24D*	2.73

L3_54934	*Protein transport protein Sec61 subunit beta*	1.61
L12_80866	*Protein tweety homolog 2-like*	1.50
L1_64341	*Protein unc-13 homolog D*	-1.64
L3_79473	*Protein unc-45 homolog B*	-2.41
L3_37477	*Protein unc-79 homolog*	2.32
L2_22224	*Protein Wnt-2b*	-2.34
L2_22353	*Protein Wnt-6*	1.59
L12_88671	*Protein wntless homolog*	-1.62
L2_66175	*Protein XRP2*	-1.59
L12_83346	*Protein YIF1A*	1.63
L12_84458	*Protein YIPF1*	-1.86
L2_59615	*Protein yippee-like 2*	-1.84
L12_73796	*Proteinase-activated receptor 3*	-2.21
L12_83139	*Proteinase-activated receptor 4*	-1.81
L12_90038	*Protein-L-isoaspartate O-methyltransferase domain-containing protein 1*	-1.69
L2_41792	*Protein-methionine sulfoxide oxidase MICAL2*	-1.84
L3_68107	*Protein-tyrosine sulfotransferase 1*	1.63
L12_46450	*Proteolipid protein 2-like*	-1.68
L3_74904	*Prothrombin*	3.97
L1_35661	*Protocadherin alpha-11*	-1.50
L2_67547	*Protocadherin Fat 4-like*	1.91
L1_63272	*Protocadherin-7*	-1.76
L2_30865	*Proton myo-inositol cotransporter*	-2.03
L12_87615	*Proto-oncogene tyrosine-protein kinase LCK*	-2.14
L1_58528	*Proto-oncogene tyrosine-protein kinase Yrk*	-1.52
L12_89279	*Proto-oncogene vav*	-1.71
L12_87948	*P-selectin glycoprotein ligand 1-like*	-1.59
L12_74243	*Pseudopodium-enriched atypical kinase 1*	-1.83
L3_77487	*Pseudouridine-5'-phosphate glycosidase*	6.73
L2_67935	*Pseudouridine-metabolizing bifunctional protein C1861.05-like*	-1.86
L2_48094	*Pterin-4-alpha-carbinolamine dehydratase*	2.40
L2_45423	*Puratrophin-1*	-2.32
L2_41257	*Purine nucleoside phosphorylase*	-37.59
L12_75145	*Purpurin*	-1.89
L12_83898	*Putative adenosylhomocysteinase 2*	-2.19
L1_75726	*Putative adenosylhomocysteinase 3*	4.29
L12_87905	*Putative all-trans-retinol 13,14-reductase*	2.89
L12_75450	*Putative all-trans-retinol 13,14-reductase '*	10.22
L1_58152	*Putative ATP-dependent RNA helicase DDX11-like protein 8*	1.82
L2_17836	*Putative cation exchanger C521.04c-like*	-1.66
L3_77891	*Putative cystathionine beta-synthase Rv1077*	1.83
L12_87719	*Putative deoxyribonuclease TATDN2*	-2.70

L12_87739	*Putative ferric-chelate reductase 1*	3.08
L12_81371	*Putative gamma-glutamyltransferase YwrD*	2.47
L12_57189	*Putative glycerol kinase 5*	3.50
L12_87623	*Putative golgin subfamily A member 6-like protein 6*	-1.76
L1_37947	*Putative helicase Mov10l1*	1.93
L12_87979	*Putative hexokinase HKDC1*	1.62
L2_23340	*Putative homeodomain transcription factor 2*	-1.66
L1_44157	*Putative integrase*	13.30
L2_8513	*Putative ionic transporter y4hA*	1.79
L1_26113	*Putative membrane protein*	5.84
L2_31090	*Putative methyltransferase*	1.59
L2_34574	*Putative methyltransferase NSUN7*	-1.72
L1_22388	*Putative monooxygenase p33MONOX*	3.04
L2_38522	*Putative NADH dehydrogenase FAD-containing subunit transmembrane protein*	7.01
L12_85605	*Putative P2Y purinoceptor 10*	-2.05
L12_87600	*Putative phospholipase B-like 2*	-3.01
L2_36038	*Putative Polycomb group protein ASXL2-like*	2.86
L1_54209	*Putative protein FAM200B*	1.71
L12_83353	*Putative serine protease K12H4.7*	-3.12
L12_89954	*Putative sodium-coupled neutral amino acid transporter 7*	-7.49
L12_89700	*Putative sodium-coupled neutral amino acid transporter 9*	-2.83
L12_29784	*Putative surface antigen, partial*	1.64
L1_40383	*Putative transporter SVOPL*	2.62
L1_26111	*Putative type III restriction-modification system HindVIP enzyme mod*	1.82
L12_83805	*Putative tyrosine-protein phosphatase auxilin*	-1.59
L12_85498	*Putative ubiquitin carboxyl-terminal hydrolase 50*	-1.56
L1_59475	*Putative uncharacterized protein*	1.63
L2_21996	*Putative uncharacterized protein*	-15.06
L3_50439	*Putative uncharacterized protein C10orf122-like*	-2.54
L12_71425	*Putative unconventional myosin-XVB*	1.56
L1_26773	*PX*	-45.71
L12_86311	*Pyridoxine-5'-phosphate oxidase*	1.95
L12_85568	*Pyruvate kinase muscle isozyme*	3.62
L2_60038	*Queuine tRNA-ribosyltransferase subunit qtrtd1*	-1.91
L12_72023	*Quinone oxidoreductase*	1.73
L12_89837	*Quinone oxidoreductase-like protein 1*	-1.64
L12_77001	*Rab GTPase-activating protein 1*	-4.58
L12_86678	*Rab GTPase-binding effector protein 2*	2.03
L1_30174	*Rab GTPase-binding effector protein 2-like*	2.33
L12_84616	*Rab11 family-interacting protein 1*	2.77
L1_44876	*Rab11 family-interacting protein 3*	-3.32
L2_34386	*Rab11 family-interacting protein 4A*	1.91
L1_24499	*Rab-3A-interacting protein*	1.80

L12_87056	*Rab5 GDP/GTP exchange factor*	-1.64
L3_51659	*Rab5 GDP/GTP exchange factor-like*	-2.11
L3_63808	*Rabenosyn-5*	-1.67
L3_62938	*Rab-like protein 5*	3.11
L3_76575	*Rad1 variant C*	-1.51
L12_83048	*RAD9, HUS1, RAD1-interacting nuclear orphan protein 1*	2.22
L12_36115	*RAD9, HUS1, RAD1-interacting nuclear orphan protein 1-like*	1.92
L12_65244	*Raftlin*	-1.87
L3_64712	*Ragulator complex protein LAMTOR2*	-1.64
L3_53079	*Ragulator complex protein LAMTOR3*	-1.59
L12_33036	*Ragulator complex protein LAMTOR4*	-1.80
L12_22517	*Ragulator complex protein LAMTOR5*	-1.59
L12_88238	*Ral GTPase-activating protein subunit alpha-2*	-1.89
L1_45601	*Ral GTPase-activating protein subunit beta*	-2.28
L3_55588	*Rano class II histocompatibility antigen, A beta chain*	-1.75
L12_79259	*Rap guanine nucleotide exchange factor 4*	2.88
L1_29156	*Rap guanine nucleotide exchange factor 5*	-1.75
L2_50650	*Rap1 GTPase-activating protein 1-like*	-21.21
L12_82639	*Rap1 GTPase-activating protein 2*	-1.63
L1_56856	*Ras and Rab interactor 2-like*	1.59
L12_69001	*Ras and Rab interactor 3*	-1.94
L12_87035	*Ras association domain-containing protein 1*	-1.70
L12_80311	*Ras association domain-containing protein 4*	-1.82
L1_34684	*Ras association domain-containing protein 5*	-1.62
L3_61281	*Ras association domain-containing protein 6*	1.89
L2_54897	*Ras association domain-containing protein 7*	1.60
L2_18401	*Ras association domain-containing protein 9*	-1.59
L12_83716	*Ras GTPase-activating protein 2*	-1.78
L2_60525	*Ras GTPase-activating protein 3*	-2.98
L12_80975	*Ras GTPase-activating protein SynGAP*	-1.95
L1_63901	*RAS guanyl-releasing protein 4*	-1.58
L12_80666	*RAS protein activator like-3*	-1.90
L2_21292	*Ras-associated and pleckstrin homology domains-containing protein 1*	4.48
L1_42812	*Ras-GEF*	1.83
L12_76111	*Ras-GEF domain-containing family member 1B-A*	-1.64
L1_30405	*Ras-GEF domain-containing family member 1C*	1.77
L12_83329	*Ras-like protein family member 11B*	2.16
L12_73622	*Ras-like protein family member 12*	-6.55
L12_80267	*Ras-related and estrogen-regulated growth inhibitor*	-2.06
L12_76354	*Ras-related C3 botulinum toxin substrate 2*	-2.10
L12_82993	*Ras-related GTP-binding protein A*	-1.70
L12_87620	*Ras-related GTP-binding protein C*	-1.77
L2_39190	*Ras-related GTP-binding protein D*	-4.01

L3_39007	*Ras-related protein O-RAL*	-1.84
L12_75859	*Ras-related protein Rab-14*	-1.98
L12_71559	*Ras-related protein Rab-17*	-2.11
L12_87112	*Ras-related protein Rab-18-B*	-1.61
L12_84649	*Ras-related protein Rab-19*	2.70
L2_19620	*Ras-related protein Rab-23*	-1.51
L12_82721	*Ras-related protein Rab-33B*	-3.28
L12_76648	*Ras-related protein Rab-34*	-1.57
L12_84549	*Ras-related protein Rab-38*	-3.77
L2_56527	*Ras-related protein Rab-39B*	2.18
L12_58694	*Ras-related protein Rab-3D*	-1.78
L12_86737	*Ras-related protein Rab-44*	-1.52
L1_46098	*Ras-related protein Rab-4B*	-1.72
L12_68513	*Ras-related protein Rab-6A*	-2.16
L12_88387	*Ras-related protein Rab-7a*	-1.58
L12_73994	*Ras-related protein Rab-9A*	-1.77
L1_49082	*Ras-related protein RABA1a*	-2.78
L2_61237	*Ras-specific guanine nucleotide-releasing factor 1*	-12.18
L1_11980	*Receptor activity-modifying protein 1*	3.15
L3_65895	*Receptor expression-enhancing protein 5*	1.54
L2_46605	*Receptor tyrosine-protein kinase erbB-2*	1.66
L2_41884	*Receptor tyrosine-protein kinase erbB-2-like*	1.68
L2_66563	*Receptor-interacting serine/threonine-protein kinase 2*	-2.52
L2_42621	*Receptor-interacting serine/threonine-protein kinase 3*	-1.66
L2_25289	*Receptor-interacting serine/threonine-protein kinase 4*	-1.56
L12_89333	*Receptor-type tyrosine-protein phosphatase C*	-1.86
L3_55331	*Receptor-type tyrosine-protein phosphatase delta*	5.57
L1_37095	*Receptor-type tyrosine-protein phosphatase eta*	1.75
L12_72696	*Receptor-type tyrosine-protein phosphatase gamma*	-1.61
L2_31254	*Receptor-type tyrosine-protein phosphatase-like N*	-2.37
L2_39941	*Regulating synaptic membrane exocytosis protein 3*	-2.20
L1_40310	*Regulating synaptic membrane exocytosis protein 4*	2.12
L1_35535	*Regulator of G-protein signaling 1*	1.96
L12_42240	*Regulator of G-protein signaling 18*	-1.80
L2_41800	*Regulator of G-protein signaling 3*	-1.67
L12_75432	*Regulator of G-protein signaling 5*	-1.57
L1_51577	*Regulator of G-protein signaling 9*	2.10
L2_4647	*Regulator of microtubule dynamics protein 2*	2.94
L12_81184	*RelA-associated inhibitor*	2.78
L1_22381	*Relaxin family locus A type 2*	40.09
L2_55928	*Relaxin-3 receptor 1*	1.97
L3_30010	*Renin*	1.58
L12_84855	*Renin receptor*	-2.43
L2_67383	*Replicase/helicase/endonuclease*	-1.99
L1_45076	*Repressor protein C*	3.31

L2_42805	*Repulsive guidance molecule A*	1.67
L12_59449	*Reticulon-3*	1.57
L3_78706	*Retinal rod rhodopsin-sensitive cGMP 3',5'-cyclic phosphodiesterase subunit delta*	-1.60
L4_19640	*Retinoic acid receptor alpha*	2.13
L2_50039	*Retinoic acid receptor gamma-A*	-1.92
L4_54575	*Retinoic acid receptor RXR-alpha-A*	5.10
L12_85142	*Retinoic acid receptor RXR-gamma-B*	2.00
L12_81389	*Retinoid-inducible serine carboxypeptidase*	-5.03
L12_88450	*Retinol dehydrogenase 11*	1.84
L2_68817	*Retinol dehydrogenase 11 (All-trans/9-cis/11-cis)*	2.75
L1_33890	*Retinol dehydrogenase 12*	9.92
L12_89129	*Retinol dehydrogenase 8*	1.53
L3_66597	*Retinol-binding protein 1*	1.98
L1_66763	*Retinol-binding protein 3*	55.20
L3_78060	*Retrovirus-related Pol polyprotein from transposon 297*	1.96
L12_85978	*Reverse transcriptase-like protein-like*	6.16
L3_73652	*Rho GDP-dissociation inhibitor 1*	-1.91
L12_83148	*Rho GTPase-activating protein 15*	-1.89
L1_33729	*Rho GTPase-activating protein 24*	-1.80
L3_78115	*Rho GTPase-activating protein 25*	-1.77
L2_60980	*Rho GTPase-activating protein 26*	-7.83
L1_66660	*Rho GTPase-activating protein 30*	-1.92
L12_88320	*Rho GTPase-activating protein 32*	1.93
L12_68718	*Rho GTPase-activating protein 9-like*	-2.60
L2_65526	*Rho guanine nucleotide exchange factor 10-like protein*	-5.94
L1_20138	*Rho guanine nucleotide exchange factor 11-like*	-1.73
L12_88692	*Rho guanine nucleotide exchange factor 16*	1.75
L12_75323	*Rho guanine nucleotide exchange factor 18-like*	1.78
L1_78094	*Rho guanine nucleotide exchange factor 26*	2.07
L12_86232	*Rho guanine nucleotide exchange factor 3*	-1.92
L12_77575	*Rho guanine nucleotide exchange factor 7*	-3.42
L1_45056	*Rhodopsin kinase-like*	-2.03
L12_82619	*Rhomboid-related protein 4*	-1.66
L2_44130	*Rhombotin-1*	-2.04
L1_69759	*Rho-related BTB domain-containing protein 2*	1.76
L12_84114	*Rho-related GTP-binding protein Rho6*	-2.09
L12_86492	*Rho-related GTP-binding protein RhoF*	-1.73
L2_44823	*Rho-related GTP-binding protein RhoQ*	-1.77
L12_88617	*Rho-related GTP-binding protein RhoU*	1.76
L2_23807	*Rhotekin-2*	1.56
L2_50626	*Ribonuclease inhibitor*	-1.80
L12_83080	*Ribonuclease T2*	-1.82
L12_87339	*Ribose-phosphate pyrophosphokinase 1*	-1.53
L1_18352	*Ribosomal L7Ae*	-2.45

L12_89050	*Ribosomal protein S6 kinase beta-1*	1.63
L1_41693	*Ribosome-binding protein 1*	-1.87
L1_75407	*RIIa domain-containing protein 1*	2.35
L12_89274	*RILP-like protein 1*	-2.12
L12_57668	*RILP-like protein 2*	-1.59
L12_90183	*RING finger protein 113A*	-1.62
L12_88658	*RING finger protein 114*	-1.94
L12_88593	*RING finger protein 121*	2.34
L3_78338	*RING finger protein 141*	1.88
L2_38678	*RING finger protein 157*	-2.64
L1_54963	*RING finger protein 175*	-1.50
L12_78334	*RING finger protein 186-like*	-37.59
L12_88492	*RING finger protein 223*	-2.25
L12_76290	*RING finger protein 24*	-1.59
L3_76739	*RING finger protein 44*	1.67
L2_30218	*RNA exonuclease 1 homolog*	-3.22
L12_82030	*RNA polymerase II elongation factor ELL*	-2.19
L12_89508	*RNA polymerase-associated protein RTF1 homolog*	23.24
L12_55599	*RNA-binding protein 24*	-3.06
L3_52112	*RNA-binding protein 24-B*	2.06
L3_80392	*RNA-binding protein Musashi homolog 1*	2.43
L12_76309	*RNA-binding protein with multiple splicing*	-4.69
L3_29398	*RNA-binding Raly-like protein*	-2.12
L1_64958	*RNA-directed DNA polymerase from mobile element jockey-like*	-2.14
L12_57084	*Rootletin*	-1.58
L12_86326	*RPE-retinal G protein-coupled receptor*	3.81
L2_32626	*RRNA methyltransferase 1, mitochondrial*	2.04
L1_18649	*RRP12-like protein*	1.55
L12_77213	*R-spondin-3*	-1.88
L12_85814	*RUN and FYVE domain-containing protein 1*	-1.74
L2_67608	*Run domain Beclin-1 interacting and cystein-rich containing protein*	-1.55
L12_77407	*RWD domain-containing protein 2B*	1.60
L2_66276	*Ryanodine receptor 2*	-2.44
L12_44324	*S-(hydroxymethyl)glutathione dehydrogenase*	1.84
L12_32054	*S100 calcium binding protein A16*	-1.84
L3_56122	*SAC3 domain-containing protein 1*	1.70
L12_87641	*Saccharopine dehydrogenase-like oxidoreductase*	3.75
L12_79126	*S-adenosylmethionine synthase isoform type-2*	3.80
L12_54226	*SAM and SH3 domain-containing protein 1*	-2.02
L12_85839	*SAM and SH3 domain-containing protein 3*	-2.08
L12_86126	*SAM domain and HD domain-containing protein 1*	-4.39
L12_73305	*SAM domain-containing protein SAMSN-1*	-1.80
L2_15495	*SAM pointed domain containing ETS transcription factor*	-1.54

L12_79974	*SAM pointed domain-containing Ets transcription factor*	-2.00
L12_84587	*Sarcolemmal membrane-associated protein*	1.97
L12_49628	*Sarcosine dehydrogenase, mitochondrial*	2.24
L12_85828	*Sarcospan*	-2.04
L2_67218	*SCAN domain-containing protein 3*	-3.78
L12_84913	*Scavenger receptor class B member 1*	30.98
L2_74679	*Scavenger receptor cysteine-rich type 1 protein M130*	-1.91
L1_51356	*Scavenger receptor cysteine-rich type 1 protein M130-like*	1.61
L12_83548	*Scavenger receptor cysteine-rich type 1 protein M160*	-1.78
L12_85089	*Schwannomin-interacting protein 1*	3.13
L12_86564	*Schwannomin-interacting protein 1-like*	-1.61
L12_86579	*Sciellin*	2.90
L1_55250	*SCO-spondin*	2.73
L12_73401	*Sec1 family domain-containing protein 1*	1.70
L1_54418	*SEC14-like protein 2*	4.00
L1_22297	*SEC14-like protein 3*	4.79
L2_46327	*SEC14-like protein 5*	-1.67
L3_78195	*SEC23-interacting protein*	1.61
L1_47150	*Secretagogin*	-4.43
L2_30962	*Secreted frizzled-related protein 1*	-3.16
L12_83962	*Secreted frizzled-related protein 2-like*	-5.39
L1_22618	*Secreted frizzled-related protein 5*	-7.61
L12_86685	*Secretogranin-3*	2.32
L1_4803	*Secretory carrier membrane protein 1*	1.72
L2_30943	*Secretory carrier-associated membrane protein 3*	-3.00
L12_87670	*Secretory carrier-associated membrane protein 4*	-3.80
L12_74708	*Secretory phospholipase A2 receptor-like*	2.41
L1_35671	*Selenide, water dikinase 2*	1.52
L12_89654	*Selenium-binding protein 1*	1.69
L1_37472	*Selenocysteine insertion sequence-binding protein 2-like*	1.84
L12_89066	*Selenoprotein L*	1.82
L1_43957	*Selenoprotein N*	-3.50
L12_39898	*Selenoprotein T2*	1.54
L12_73309	*Semaphorin-3A*	-1.80
L3_77560	*Semaphorin-3ab*	-1.53
L2_56441	*Semaphorin-3C*	-1.97
L2_35711	*Semaphorin-3G*	-1.77
L2_42719	*Semaphorin-4A*	-1.54
L2_66904	*Semaphorin-4C*	1.82
L2_25302	*Semaphorin-4D*	-2.79
L2_41658	*Semaphorin-4G*	2.13
L12_88980	*Semaphorin-6D*	2.16
L3_78401	*Sentrin-specific protease 8*	1.60
L2_31565	*Septin-12*	-1.84
L12_89612	*Septin-9*	-1.62

L2_49471	*Serine hydrolase-like protein-like*	-2.06
L12_85620	*Serine hydroxymethyltransferase, cytosolic*	-4.22
L12_88557	*Serine hydroxymethyltransferase, mitochondrial*	2.86
L12_56982	*Serine incorporator 2*	-2.33
L2_55272	*Serine incorporator 3*	-1.75
L12_90086	*Serine palmitoyltransferase 2*	-1.98
L12_88726	*Serine palmitoyltransferase 3*	-2.53
L2_26037	*Serine protease HTRA3*	1.71
L12_85711	*Serine/threonine-protein kinase 24*	2.31
L2_58463	*Serine/threonine-protein kinase 32A-like*	3.63
L1_42086	*Serine/threonine-protein kinase 4*	-1.56
L2_26080	*Serine/threonine-protein kinase haspin-like*	1.56
L12_90229	*Serine/threonine-protein kinase LMTK2*	-2.75
L1_67143	*Serine/threonine-protein kinase Nek1*	-1.58
L12_81401	*Serine/threonine-protein kinase Nek2*	2.04
L2_57717	*Serine/threonine-protein kinase par-1*	-2.06
L12_61478	*Serine/threonine-protein kinase pim-3*	-1.80
L1_55891	*Serine/threonine-protein kinase RIO3*	1.63
L2_25886	*Serine/threonine-protein kinase Sgk1-like*	-1.82
L12_87832	*Serine/threonine-protein kinase Sgk3*	-1.51
L2_63869	*Serine/threonine-protein kinase TAO1*	4.54
L3_69964	*Serine/threonine-protein kinase TNNI3K*	-1.67
L12_36796	*Serine/threonine-protein kinase VRK2*	2.74
L3_77894	*Serine/threonine-protein phosphatase 2A 55 kDa regulatory subunit B delta isoform*	1.76
L3_73468	*Serine/threonine-protein phosphatase 2A catalytic subunit alpha isoform*	-2.37
L12_86378	*Serine/threonine-protein phosphatase 2A catalytic subunit beta isoform*	1.58
L12_83784	*Serine/threonine-protein phosphatase 2A regulatory subunit B'' subunit gamma*	-1.51
L12_85619	*Serine-tRNA ligase, mitochondrial*	1.59
L1_56706	*Serpin H1-like*	2.64
L1_57768	*Serum amyloid P-component*	41.01
L12_85181	*Sesquipedalian-1*	-1.65
L12_83986	*Sestrin-1*	-1.67
L2_64654	*Sestrin-2*	-2.15
L2_73728	*SH2 domain-containing adapter protein F*	-2.05
L12_73720	*SH2 domain-containing protein 1A*	-3.81
L3_77582	*SH2 domain-containing protein 5*	1.67
L1_32384	*SH2B adapter protein 3*	-3.26
L2_66787	*SH2B adapter protein 3-like*	-1.76
L2_68558	*SH3 and PX domain-containing protein 2A*	-1.56
L12_85622	*SH3 domain and tetratricopeptide repeat-containing protein 1*	-36.37

L2_27235	*SH3 domain-binding glutamic acid-rich-like protein 3*	2.02
L3_77024	*SH3 domain-binding protein 1*	-1.66
L12_24905	*SH3 domain-binding protein 1-like*	-1.93
L3_78026	*SH3 domain-binding protein 5*	-3.27
L1_51821	*SH3 domain-binding protein 5-like*	1.75
L1_67897	*SHC-transforming protein 3*	-2.53
L12_87643	*Short coiled-coil protein A*	-1.87
L2_58475	*Short transient receptor potential channel 1*	-3.18
L12_85051	*Short-chain dehydrogenase/reductase 3*	-2.04
L12_81308	*Sialic acid-binding Ig-like lectin 9-like*	-3.06
L12_84085	*Sialidase-1*	-6.43
L3_75379	*Sialidase-3-like*	2.95
L12_89022	*Sialin*	-5.03
L12_77068	*Sialoadhesin*	-4.26
L12_90092	*Sideroflexin-1*	18.71
L12_84789	*Sideroflexin-2*	-3.23
L12_88093	*Sigma non-opioid intracellular receptor 1*	2.20
L2_30368	*Signal peptide peptidase-like 2A-like*	1.58
L12_89927	*Signal recognition particle receptor subunit beta*	1.61
L2_70077	*Signal transducer and activator of transcription 1*	-1.51
L3_78256	*Signal transducer and activator of transcription 1-like*	-1.60
L3_77138	*Signal transducer and activator of transcription 5B*	-1.56
L12_82336	*Signal-induced proliferation-associated 1-like protein 1*	2.36
L12_72342	*Signal-transducing adaptor protein 1*	-2.43
L12_89854	*Signal-transducing adaptor protein 2*	2.15
L12_88132	*Single Ig IL-1-related receptor*	-1.87
L2_32632	*Single-strand binding protein/primosomal replication protein N*	1.57
L2_44561	*Single-stranded DNA-binding protein 3*	1.61
L1_64687	*Ski oncogene*	-1.64
L2_32187	*SLA class II histocompatibility antigen, DQ haplotype D beta chain*	-5.53
L2_64567	*Slit homolog 2 protein*	1.68
L2_55457	*SLIT-ROBO Rho GTPase-activating protein 3*	-1.81
L12_84939	*Small conductance calcium-activated potassium channel protein 1*	-28.10
L3_78170	*Small glutamine-rich tetratricopeptide repeat-containing protein alpha*	1.74
L12_82259	*Small glutamine-rich tetratricopeptide repeat-containing protein beta*	-1.61
L2_61551	*Smith-Magenis syndrome chromosomal region candidate gene 8 protein homolog*	-1.88
L12_77032	*Smoothelin-like protein 1*	-2.08
L2_65008	*Smoothelin-like protein 2*	2.89
L1_17120	*SNF-related serine/threonine-protein kinase*	3.15

L12_82556	*SNF-related serine/threonine-protein kinase-like*	8.53
L1_37369	*Sodium- and chloride-dependent betaine transporter*	5.84
L12_43772	*Sodium- and chloride-dependent creatine transporter 1*	1.59
L2_56557	*Sodium- and chloride-dependent GABA transporter 2*	13.28
L1_66068	*Sodium- and chloride-dependent GABA transporter 2-like*	2.38
L12_88567	*Sodium- and chloride-dependent GABA transporter ine*	2.32
L12_89793	*Sodium- and chloride-dependent taurine transporter*	-4.13
L2_61256	*Sodium bicarbonate cotransporter 3*	-1.64
L12_75006	*Sodium bicarbonate transporter-like protein 11*	-5.19
L2_38282	*Sodium bicarbonate transporter-like protein 11-like*	-4.21
L12_90202	*Sodium channel and clathrin linker 1*	4.52
L1_5673	*Sodium channel protein type 4 subunit alpha A*	-2.26
L3_79476	*Sodium channel protein type 4 subunit alpha B*	-1.55
L2_62039	*Sodium phosphate cotransporter*	2.07
L3_78227	*Sodium/calcium exchanger 1*	-2.56
L12_89703	*Sodium/glucose cotransporter 1*	2.19
L12_72825	*Sodium/hydrogen exchanger*	1.68
L1_59698	*Sodium/hydrogen exchanger 7*	-3.22
L12_61909	*Sodium/hydrogen exchanger 9*	-1.97
L12_90063	*Sodium/myo-inositol cotransporter 2*	1.52
L2_48515	*Sodium/nucleoside cotransporter*	-1.77
L1_47292	*Sodium/nucleoside cotransporter 1*	3.43
L1_45646	*Sodium/nucleoside cotransporter 1-like*	2.67
L12_88029	*Sodium/nucleoside cotransporter 2*	3.32
L12_82294	*Sodium/potassium/calcium exchanger 5*	1.82
L3_77882	*Sodium/potassium-transporting ATPase subunit alpha-1*	-1.62
L2_67429	*Sodium/potassium-transporting ATPase subunit alpha-3*	2.00
L3_69270	*Sodium/potassium-transporting ATPase subunit beta-233*	1.70
L12_89323	*Sodium-coupled monocarboxylate transporter 1*	2.52
L12_81066	*Sodium-coupled neutral amino acid transporter 2*	-1.76
L2_62152	*Sodium-dependent multivitamin transporter*	-2.07
L12_86256	*Sodium-dependent multivitamin transporter-like*	-2.20
L12_85793	*Sodium-dependent neutral amino acid transporter B(0)AT1*	7.48
L12_88541	*Sodium-dependent neutral amino acid transporter B(0)AT3*	-2.37
L3_80393	*Sodium-dependent phosphate transport protein 2A*	74.50
L12_88454	*Sodium-dependent phosphate transport protein 2B*	6.10
L2_36706	*Sodium-dependent phosphate transporter 1-A*	-2.14
L2_60721	*Sodium-dependent phosphate transporter 2*	-2.36
L2_51378	*Sodium-independent sulfate anion transporter*	-5.66
L3_77071	*Solute carrier family 12 member 2*	2.32
L12_54130	*Solute carrier family 12 member 9*	-1.81
L2_68667	*Solute carrier family 12 member 9-like*	-1.52
L1_64109	*Solute carrier family 13 member 2*	1.92

L12_85550	*Solute carrier family 13 member 3*	-8.19
L12_89622	*Solute carrier family 13 member 5*	7.95
L2_17382	*Solute carrier family 13 member 5-like*	1.85
L12_88402	*Solute carrier family 15 member 1*	87.48
L2_89755	*Solute carrier family 15 member 2*	-52.26
L2_20523	*Solute carrier family 15 member 4*	-2.67
L2_36527	*Solute carrier family 2, facilitated glucose transporter member 1*	-1.79
L4_88235	*Solute carrier family 2, facilitated glucose transporter member 2*	3.04
L12_90152	*Solute carrier family 2, facilitated glucose transporter member 4*	-1.62
L12_84958	*Solute carrier family 2, facilitated glucose transporter member 5*	41.64
L1_52131	*Solute carrier family 2, facilitated glucose transporter member 5-like*	12.27
L12_86726	*Solute carrier family 2, facilitated glucose transporter member 8*	-2.32
L12_85204	*Solute carrier family 2, facilitated glucose transporter member 9*	2.28
L12_87856	*Solute carrier family 22 member 13*	1.61
L1_59274	*Solute carrier family 22 member 23*	-1.69
L2_69689	*Solute carrier family 22 member 3*	-10.22
L3_47481	*Solute carrier family 22 member 7-like*	1.85
L12_78474	*Solute carrier family 23 member 1*	1.59
L12_86861	*Solute carrier family 25 member 33*	-1.74
L12_74194	*Solute carrier family 25 member 35*	-7.35
L1_44485	*Solute carrier family 25 member 36-A*	2.64
L12_81850	*Solute carrier family 25 member 39*	2.06
L12_86457	*Solute carrier family 25 member 40*	1.52
L2_69996	*Solute carrier family 25 member 47-A*	-1.61
L2_20616	*Solute carrier family 25 member 47-B*	-2.16
L1_39024	*Solute carrier family 26 (anion exchanger), member 11*	-7.00
L1_89470	*Solute carrier family 26 member 6*	145.73
L1_63829	*Solute carrier family 35 member G1*	2.92
L2_55995	*Solute carrier family 41 member 1*	-2.07
L2_59419	*Solute carrier family 5 (sodium/choline cotransporter), member 7*	-2.07
L12_84916	*Solute carrier family 52, riboflavin transporter, member 2*	1.62
L12_84194	*Solute carrier organic anion transporter family member 1C1-like*	-1.57
L12_88096	*Solute carrier organic anion transporter family member 2A1*	6.55
L1_21327	*Somatostatin receptor type 2*	1.81
L3_60912	*Somatostatin-like receptor F 48D10.1*	2.58

L2_51722	*Sorbin and SH3 domain-containing protein 1*	-1.88
L12_81429	*Sorbitol dehydrogenase*	3.05
L2_67782	*Sortilin-related receptor*	-1.92
L12_89523	*Sorting nexin-1*	-2.48
L12_82520	*Sorting nexin-14*	-1.58
L12_88603	*Sorting nexin-17*	-1.80
L12_85491	*Sorting nexin-18*	12.76
L3_63908	*Sorting nexin-19-like*	-1.73
L1_56675	*Sorting nexin-2*	-6.50
L1_46967	*Sorting nexin-27*	-2.23
L1_51313	*Sorting nexin-29*	-1.70
L12_76467	*Sorting nexin-3*	-2.05
L2_64091	*Sorting nexin-30*	-1.56
L12_80159	*Sorting nexin-4*	-3.21
L12_82553	*Sorting nexin-6-like*	-55.27
L12_86543	*Sorting nexin-7*	-1.50
L12_90055	*Sorting nexin-8*	-10.65
L12_68587	*SOSS complex subunit C*	-1.80
L12_74109	*SPARC-related modular calcium-binding protein 2*	2.56
L12_73045	*Spatacsin*	-1.52
L2_49782	*Spectrin beta chain, non-erythrocytic 1*	1.86
L2_48836	*Spectrin beta chain, non-erythrocytic 2*	-1.71
L3_69641	*Speriolin-like protein*	-1.57
L1_30929	*Sperm-associated antigen 16 protein*	2.44
L2_63770	*Sperm-associated antigen 17*	1.96
L2_37257	*Spermatid perinuclear RNA-binding protein*	-2.20
L3_71046	*Spermatogenesis-associated protein 17*	1.57
L1_32608	*Spermatogenesis-associated protein 20*	2.04
L3_80123	*Spermatogenesis-associated protein 6*	2.51
L1_53539	*Spermatogenesis-associated protein 7 homolog*	-1.65
L2_48512	*Spermatogenesis-associated serine-rich protein 2*	-2.43
L12_86945	*Spermine oxidase*	-2.38
L3_80004	*Sphingolipid delta(4)-desaturase DES1*	1.59
L12_88732	*Sphingomyelin phosphodiesterase*	-2.95
L2_41481	*Sphingomyelin synthase-related protein 1*	4.55
L12_85411	*Sphingosine 1-phosphate receptor 4*	-15.40
L1_15823	*Sphingosine-1-phosphate lyase 1*	2.47
L12_88432	*Sphingosine-1-phosphate phosphatase 1*	2.21
L12_72462	*Spindle and centriole-associated protein 1*	-2.25
L12_74671	*Spindle and kinetochore-associated protein 1*	-1.78
L3_44204	*Splicing factor 3A subunit 1*	1.56
L12_86545	*Spondin-2*	4.12
L1_30299	*Spondin-2-like*	-2.14
L12_84906	*SPRY domain-containing protein 7*	1.67
L3_36637	*Squidulin*	1.57

L1_51115	*SRC kinase signaling inhibitor 1*	1.65
L12_83546	*Src kinase-associated phosphoprotein 1*	-2.75
L12_82229	*Src-like-adapter 2*	-1.68
L12_84505	*StAR-related lipid transfer protein 3*	-1.56
L1_56701	*Sterile alpha and TIR motif-containing protein 1*	2.13
L12_65711	*Steroidogenic acute regulatory protein, mitochondrial*	1.99
L1_65158	*Steroidogenic factor 1*	2.22
L12_79258	*Sterol 26-hydroxylase, mitochondrial*	1.71
L12_87941	*Sterol O-acyltransferase 1*	7.78
L4_72701	*Sterol regulatory element-binding protein 1*	2.03
L1_52273	*Sterol regulatory element-binding protein cleavage-activating protein*	2.54
L12_65411	*Stimulated by retinoic acid gene 6 protein homolog*	1.66
L12_88931	*Stonin-1*	2.93
L1_63933	*Stonustoxin subunit alpha*	-2.66
L12_86651	*Store-operated calcium entry-associated regulatory factor*	-1.54
L2_45544	*Stress-70 protein, mitochondrial*	1.57
L12_30081	*Stress-associated endoplasmic reticulum protein 1*	3.35
L1_20003	*Stromal interaction molecule 2-like*	-1.63
L12_87473	*Structural maintenance of chromosomes flexible hinge domain-containing protein 1*	2.00
L12_88142	*Succinate dehydrogenase flavoprotein subunit, mitochondrial*	3.10
L12_73944	*Succinate dehydrogenase assembly factor 2, mitochondrial*	-1.52
L12_44135	*Succinate dehydrogenase cytochrome b560 subunit, mitochondrial*	1.61
L12_80341	*Succinyl-CoA ligase subunit alpha, mitochondrial*	2.35
L12_87645	*Succinyl-CoA ligase subunit beta, mitochondrial*	1.97
L12_88639	*Succinyl-CoA:3-ketoacid coenzyme A transferase 1, mitochondrial*	-2.71
L1_25918	*Succinyl-CoA:coenzyme A transferase*	1.54
L12_84181	*Sucrase-isomaltase, intestinal*	1.79
L12_83895	*Sugar phosphate exchanger 2*	-1.60
L3_72990	*Sulfatase-modifying factor 1*	-2.94
L2_63919	*Sulfate transporter*	-1.98
L1_30626	*Sulfide-quinone reductase*	1.72
L2_55455	*Sulfite oxidase, mitochondrial*	-8.36
L12_88179	*Sulfotransferase 6B1*	2.29
L2_35585	*Sulfotransferase family cytosolic 2B member 1-like*	-2.52
L12_77810	*SUN domain-containing ossification factor*	1.85
L2_21558	*SUN domain-containing protein 1*	-1.55
L3_71672	*Superoxide dismutase , mitochondrial*	1.82
L12_84301	*Suppressor of cytokine signaling 1*	-1.63
L12_84696	*Suppressor of tumorigenicity 14 protein*	-1.92
L12_79639	*Surfeit locus protein 2*	2.76

L12_87354	*Sushi domain-containing protein 3*	-2.47
L12_75877	*Sushi, nidogen and EGF-like domain-containing protein 1*	-2.17
L12_72814	*Switch-associated protein 70*	-4.59
L2_58469	*Synaptic vesicle 2-related protein*	-10.77
L2_64846	*Synaptogyrin-3*	-2.08
L2_68536	*Synaptonemal complex central element protein 1-like*	-1.68
L3_79544	*Synaptonemal complex protein 2*	1.66
L12_77672	*Synaptonemal complex protein 2-like*	-2.82
L2_37262	*Synaptophysin*	-1.69
L2_54778	*Synaptophysin-like protein 1*	-1.81
L2_5367	*Synaptopodin-2-like*	-2.28
L12_89619	*Synaptosomal-associated protein 23*	-1.69
L2_6942	*Synaptotagmin-like protein 4-like*	-1.74
L2_64733	*Synaptotagmin-like protein 5*	-1.89
L12_88660	*Syncoilin*	-2.09
L12_64427	*Synemin-like*	-1.98
L2_51097	*Syntabulin*	1.79
L12_82448	*Syntaxin-10*	1.97
L12_79779	*Syntaxin-11*	-1.96
L12_79082	*Syntaxin-3*	-2.72
L3_65128	*Syntaxin-7*	5.50
L3_81654	*Syntaxin-8*	-1.65
L12_88254	*Syntaxin-binding protein 2*	-1.73
L2_50696	*Syntaxin-binding protein 5*	-1.74
L12_81838	*T cell surface glycoprotein CD3 epsilon*	-2.10
L4_47302	*Tachykinin 3*	-4.13
L2_1296	*Tachykinin receptor 3*	-1.85
L2_47007	*Talin-2*	-2.28
L2_60797	*Tankyrase-1*	1.63
L4_87300	*Tapasin*	-1.53
L12_89031	*Target of EGR1 protein 1*	1.62
L12_87974	*Target of Nesh-SH3*	-9.17
L12_79441	*Tartrate-resistant acid phosphatase type 5*	-1.59
L2_53908	*TBC domain-containing protein kinase-like protein*	-2.77
L3_79651	*TBC1 domain family member 1*	-2.10
L12_49535	*TBC1 domain family member 13*	-1.60
L12_84892	*TBC1 domain family member 24*	-3.25
L1_59519	*TBC1 domain family member 2B*	-2.73
L12_89877	*TBC1 domain family member 4*	-4.62
L12_82687	*TBC1 domain family member 5*	-1.63
L12_76650	*TBC1 domain family member 9*	-1.53
L2_62807	*T-box transcription factor TBX1*	21.61
L12_89089	*T-cell activation Rho GTPase-activating protein*	-1.65
L2_38962	*T-cell receptor beta chain ANA 11*	3.20
L3_70538	*T-cell receptor beta chain T17T-22*	-2.54

L12_37029	*T-cell receptor beta chain T17T-22-like*	-3.03
L2_79052	*T-cell receptor beta-2 chain C region*	-2.24
L4_86612	*T-cell surface glycoprotein CD4*	-4.21
L1_60391	*T-cell-specific surface glycoprotein CD28 homolog*	-4.88
L3_77967	*Tctex1 domain-containing protein 1-A*	1.74
L2_64105	*Teashirt homolog 1*	-1.67
L12_75585	*Teashirt homolog 2*	-2.17
L1_27129	*Teashirt homolog 2-like*	-2.68
L12_67888	*Tectonic-1*	-4.82
L1_33777	*Tectonin beta-propeller repeat-containing protein 1*	1.70
L1_35502	*Telomeric repeat-binding factor 1*	-1.68
L1_43537	*Telomeric repeat-binding factor 1-like*	-1.61
L12_90014	*Tensin*	-1.91
L3_62755	*Tensin-4*	3.59
L2_39544	*Tensin-like C1 domain-containing phosphatase-like*	-1.58
L12_75584	*Testin*	-2.35
L3_35361	*Testis-expressed sequence 12 protein*	-14.99
L12_85939	*Testis-expressed sequence 2 protein*	-2.55
L12_74336	*Tetranectin*	2.35
L12_86471	*Tetraspanin-1*	-18.79
L12_85175	*Tetraspanin-33*	-3.75
L12_85965	*Tetraspanin-6-like*	-1.57
L2_60322	*Tetraspanin-7*	-1.56
L12_63812	*Tetraspanin-8*	2.51
L12_89310	*Tetratricopeptide repeat protein 14*	2.72
L1_40722	*Tetratricopeptide repeat protein 23*	-1.63
L12_81639	*Tetratricopeptide repeat protein 39A*	-3.47
L12_75590	*Tetratricopeptide repeat protein 39C*	-2.02
L3_27465	*TGF-beta receptor type-1-like*	-2.79
L2_51392	*TGF-beta receptor type-2*	1.59
L1_43576	*Thiolase C*	2.29
L2_46132	*Thioredoxin domain-containing protein 16*	-1.60
L12_89834	*Thioredoxin domain-containing protein 5*	-2.70
L12_72100	*Thioredoxin reductase 3*	-1.87
L12_89780	*Thioredoxin-interacting protein*	-1.53
L12_73647	*Thioredoxin-like protein AAED1*	-1.63
L3_74051	*Thiosulfate sulfurtransferase/rhodanese-like domain-containing protein 3*	-3.34
L12_87954	*THO complex subunit 5 homolog*	1.50
L12_79535	*Threonine synthase-like 2*	3.06
L12_88524	*Thrombomodulin-like*	1.98
L1_67752	*Thrombopoietin receptor-like*	-1.67
L2_38686	*Thrombospondin-4*	2.79
L12_80382	*Thrombospondin-4-B*	2.83
L12_74598	*Thromboxane-A synthase*	-2.25

L3_82996	*Thymidylate synthase*	38.13
L12_73829	*Thymocyte nuclear protein 1*	-1.75
L12_59153	*Thymocyte selection-associated high mobility group box protein TOX-like*	2.32
L12_85737	*Thyroid hormone receptor-associated protein 3*	-5.80
L3_81063	*Thyrotropin subunit beta*	4.88
L1_15025	*Tigrin*	2.35
L3_82295	*Tissue factor pathway inhibitor*	2.10
L12_87476	*Tissue-type plasminogen activator*	1.61
L1_54952	*TLC domain-containing protein 2*	2.04
L12_85993	*T-lymphoma invasion and metastasis-inducing protein 2*	1.69
L2_54907	*T-lymphoma invasion and metastasis-inducing protein 2-like*	1.71
L12_72898	*TNFAIP3-interacting protein 2*	-1.74
L3_80084	*Toll/interleukin-1 receptor domain-containing adapter protein*	-2.48
L2_43119	*Toll-like receptor 1*	-2.33
L12_85631	*Toll-like receptor 2*	-1.67
L2_44460	*Toll-like receptor 2 type-2-like*	-2.24
L2_36831	*Toll-like receptor 6*	-2.33
L12_82498	*Toll-like receptor 8*	-1.71
L12_72608	*Toll-like receptor 8-like*	-2.15
L2_38051	*TOM1-like protein 2*	-1.55
L2_36380	*TonB-dependent receptor-like protein*	1.60
L12_56980	*Tonsoku-like protein*	1.82
L12_89118	*Torsin-1A-interacting protein 2*	1.66
L1_52136	*Torsin-2A*	2.25
L12_68268	*Torsin-3A*	-2.02
L2_13928	*TOX high mobility group box family member 2*	-1.97
L12_76044	*TPA-induced transmembrane protein homolog*	-1.53
L3_33813	*Trafficking protein particle complex subunit 2-like protein*	-1.62
L12_75800	*Trafficking protein particle complex subunit 3*	-1.52
L2_52331	*TRAF-interacting protein with FHA domain-containing protein A-like*	-2.96
L12_85547	*Trans-1,2-dihydrobenzene-1,2-diol dehydrogenase*	1.68
L12_82809	*Transaldolase*	-1.52
L12_83830	*Transcobalamin-2*	-42.14
L2_48237	*Transcription cofactor HES-6*	-1.50
L2_71470	*Transcription cofactor HES-6-like*	1.83
L3_46341	*Transcription elongation factor 1 homolog*	-1.66
L2_70468	*Transcription elongation factor B polypeptide 3*	-4.36
L12_77988	*Transcription factor 4*	-1.53
L3_79306	*Transcription factor COE1*	-3.53
L1_36967	*Transcription factor CP2-like protein 1*	8.64
L12_72296	*Transcription factor E2F6-like*	1.55

L12_62089	*Transcription factor ETV6*	3.30
L1_39434	*Transcription factor GATA-4*	52.69
L1_62752	*Transcription factor GATA-4-like*	88.16
L12_56052	*Transcription factor HES-5*	1.58
L12_80832	*Transcription factor jun-D*	1.52
L12_85569	*Transcription factor MafB*	-2.24
L12_85913	*Transcription factor MafG*	1.97
L3_77392	*Transcription factor Sox-3*	-1.55
L4_30	*Transcription factor SOX-9*	-2.12
L1_67223	*Transcription factor Sp5*	81.77
L2_29304	*Transcription factor Spi-C-like*	-2.37
L12_46966	*Transcription factor VBP*	-1.50
L12_71687	*Transcription initiation factor TFIID subunit 10*	-2.20
L1_62305	*Transcription initiation factor TFIID subunit 2*	1.53
L12_89339	*Transcription regulator protein BACH1*	-1.73
L1_42669	*Transcription-repair-coupling factor*	1.72
L1_63899	*Transducin beta-like protein 2*	1.52
L1_13922	*Transducin-like enhancer protein 3*	-1.87
L12_74657	*Transformer-2 protein homolog beta*	-1.67
L2_24084	*Transforming acidic coiled coil 1a*	-2.15
L12_87969	*Transforming growth factor beta-1-induced transcript 1 protein*	-1.93
L12_89487	*Transforming growth factor beta-3*	2.00
L2_60845	*Transforming growth factor-beta receptor-associated protein 1*	-9.97
L12_75003	*Transforming growth factor-beta-induced protein ig-h3*	-2.12
L1_48820	*Transient receptor potential cation channel subfamily M member 1*	2.24
L12_88516	*Transient receptor potential cation channel subfamily M member 2*	-4.08
L2_57743	*Transient receptor potential cation channel subfamily M member 3*	1.91
L12_76066	*Transient receptor potential cation channel subfamily M member 6*	2.24
L3_74844	*Translation initiation factor eIF-2B subunit alpha*	1.55
L1_38167	*Translational activator of cytochrome c oxidase 1-like*	1.77
L3_64212	*Translocator protein*	-2.35
L12_85021	*Translocon-associated protein subunit alpha*	1.77
L12_77773	*Transmembrane 4 L6 family member 1*	245.05
L12_81219	*Transmembrane 4 L6 family member 4*	9.54
L12_78943	*Transmembrane 4 L6 family member 5*	20.73
L3_57687	*Transmembrane 6 superfamily member 2-like*	5.55
L12_87297	*Transmembrane and coiled-coil domains protein 1*	2.42
L2_79144	*Transmembrane and immunoglobulin domain-containing protein 1*	-556.28

L3_74805	*Transmembrane and immunoglobulin domain-containing protein 1-like*	-2.71
L2_52183	*Transmembrane and TPR repeat-containing protein 2*	-2.03
L1_61041	*Transmembrane and TPR repeat-containing protein 4*	2.84
L2_35269	*Transmembrane channel-like protein*	-2.33
L2_56762	*Transmembrane channel-like protein 6-like*	1.56
L12_77642	*Transmembrane channel-like protein 8*	-2.21
L12_84997	*Transmembrane emp24 domain-containing protein 1*	-1.67
L1_49285	*Transmembrane emp24 domain-containing protein 6*	4.39
L12_86902	*Transmembrane gamma-carboxyglutamic acid protein 4*	2.06
L12_18427	*Transmembrane prostate androgen-induced protein*	1.56
L12_85770	*Transmembrane protease serine 2*	-6.20
L12_62776	*Transmembrane protease serine 4*	1.89
L4_22971	*Transmembrane protease serine 6*	-1.72
L3_75905	*Transmembrane protein 100*	2.47
L12_86373	*Transmembrane protein 104*	-1.82
L12_87680	*Transmembrane protein 106B*	3.17
L1_2425	*Transmembrane protein 117-like*	-1.60
L12_83737	*Transmembrane protein 119*	-1.75
L2_66868	*Transmembrane protein 120B*	-2.48
L12_86273	*Transmembrane protein 125-like*	-1.78
L12_89183	*Transmembrane protein 131*	1.80
L12_74207	*Transmembrane protein 134*	-2.21
L12_89282	*Transmembrane protein 135*	2.21
L12_82325	*Transmembrane protein 150B*	2.93
L1_15821	*Transmembrane protein 173-like*	1.70
L12_85222	*Transmembrane protein 179B*	-4.53
L1_9155	*Transmembrane protein 17-like*	1.53
L12_87804	*Transmembrane protein 184C*	-1.59
L1_33543	*Transmembrane protein 189*	2.08
L3_71821	*Transmembrane protein 199*	-1.99
L12_83924	*Transmembrane protein 205*	1.84
L12_79539	*Transmembrane protein 209*	-1.62
L12_88353	*Transmembrane protein 214-A*	2.39
L2_24210	*Transmembrane protein 214-B-like*	1.62
L12_81070	*Transmembrane protein 222*	-1.85
L1_50292	*Transmembrane protein 229B*	9.41
L2_46518	*Transmembrane protein 229b-like*	2.67
L2_85695	*Transmembrane protein 236*	-3825.29
L12_60467	*Transmembrane protein 238*	1.65
L1_43176	*Transmembrane protein 244*	2.84
L2_38262	*Transmembrane protein 254*	10.04
L12_87263	*Transmembrane protein 35*	-2.02
L12_86448	*Transmembrane protein 41A-A*	1.61
L1_22138	*Transmembrane protein 42-like*	1.57

L1_29044	*Transmembrane protein 47*	-1.87
L1_14111	*Transmembrane protein 51-like*	-1.91
L2_23962	*Transmembrane protein 55A*	-2.45
L12_90234	*Transmembrane protein 56-B*	2.87
L12_82147	*Transmembrane protein 63A*	-1.68
L12_84918	*Transmembrane protein 63B*	1.93
L1_41705	*Transmembrane protein 64*	3.71
L2_62153	*Transmembrane protein 68-like*	-2.30
L12_86209	*Transmembrane protein 79*	1.61
L12_85948	*Transmembrane protein 82*	1.70
L1_7335	*Transmembrane protein 87A-like*	2.30
L1_10198	*Transmembrane protein 8B*	-1.52
L3_76301	*Transmembrane protein 9B*	-3.96
L12_82819	*Transmembrane protein adipocyte-associated 1 homolog*	1.53
L12_81372	*Transmembrane protein C14orf176 homolog*	2.30
L12_83297	*Transmembrane protein C2orf18 homolog*	-5.00
L12_87060	*Transmembrane protein C9orf91 homolog*	-2.26
L12_88028	*Transmembrane protein LOC124446*	1.67
L1_18147	*Transporter*	2.71
L2_38452	*Transposon TX1 uncharacterized 149 kDa protein*	-3.33
L3_60251	*Transthyretin*	1.65
L12_89881	*Trehalase*	2.93
L1_53853	*Tricarboxylate transport protein, mitochondrial*	-2.61
L12_89266	*Trichohyalin*	-1.72
L12_87700	*Trifunctional enzyme subunit alpha, mitochondrial*	1.93
L12_73774	*Trigger factor-like protein TIG*	-1.61
L12_90126	*Trimeric intracellular cation channel type B*	1.83
L12_80352	*Triosephosphate isomerase B*	1.80
L12_85008	*Tripartite motif-containing protein 16*	1.87
L12_88360	*Tripartite motif-containing protein 2*	-1.80
L12_83294	*Tripartite motif-containing protein 39*	2.42
L1_54522	*Tripartite motif-containing protein 39-like*	6.12
L1_56505	*Tripartite motif-containing protein 46-like*	-1.55
L3_79475	*Tripartite motif-containing protein 55*	1.68
L12_81988	*Tripartite motif-containing protein 69-like*	1.94
L12_29476	*Tripeptidyl-peptidase 1*	-1.88
L12_88174	*Triple QxxK/R motif-containing protein*	1.78
L12_87349	*TRNA (adenine(58)-N(1))-methyltransferase non-catalytic subunit TRM6*	2.71
L2_25091	*TRNA (adenine(58)-N(1))-methyltransferase, mitochondrial*	2.33
L12_62475	*TRNA wybutosine-synthesizing protein 1 homolog*	-1.55
L12_82780	*TRNA-splicing endonuclease subunit Sen54*	-1.56
L12_89756	*Tropomodulin-3*	2.86
L3_61825	*Tropomyosin alpha-1 chain*	-2.63

L3_59535	*Troponin C, skeletal muscle*	2.85
L2_70525	*Troponin T, cardiac muscle isoforms*	3.01
L12_84816	*Trypsin-3*	17.68
L12_73411	*Trypsinogen-like protein 3*	63.46
L2_43714	*Tryptophan 5-hydroxylase*	-2.76
L12_85446	*Tryptophan 5-hydroxylase 2*	5.08
L1_39592	*TSC22 domain family protein 2*	1.52
L12_85236	*TSC22 domain family protein 3*	1.62
L1_48498	*T-SNARE domain-containing protein 1*	-1.78
L2_41743	*Tubby-related protein 3*	-1.68
L1_15353	*Tubby-related protein 4-like isoform 2*	6.64
L12_61288	*Tubulin alpha-1C chain*	-1.95
L3_59063	*Tubulin delta chain*	-1.81
L12_86387	*Tubulin polymerization-promoting protein family member 3*	-1.61
L1_58067	*Tudor domain-containing protein 5*	-3.85
L12_87910	*Tumor necrosis factor alpha-induced protein 2*	-1.59
L3_54401	*Tumor necrosis factor ligand superfamily member 13B*	-1.79
L12_82680	*Tumor necrosis factor ligand superfamily member 6*	-1.76
L1_44609	*Tumor necrosis factor receptor superfamily member 11B*	2.04
L12_82789	*Tumor necrosis factor receptor superfamily member 19*	1.69
L2_64932	*Tumor necrosis factor receptor superfamily member 19L*	-16.02
L3_52804	*Tumor necrosis factor receptor superfamily member 5*	-1.78
L12_75631	*Tumor necrosis factor receptor superfamily member 6*	-1.78
L12_77652	*Tumor necrosis factor receptor superfamily member 6B*	-2.73
L2_59948	*Tumor necrosis factor receptor superfamily member 6B-like*	1.80
L12_80379	*Tumor necrosis factor receptor superfamily member 9*	1.86
L3_82940	*Tumor protein D53 homolog*	-1.61
L1_37203	*Tumor protein D54-like*	1.53
L3_62467	*Tumor suppressor candidate 2*	-1.78
L12_80022	*Tumor-associated calcium signal transducer 2*	-1.51
L12_87289	*Twinfilin-1*	-1.70
L2_49817	*Two pore calcium channel protein 1-like*	-3.89
L12_72636	*Type I inositol 3,4-bisphosphate 4-phosphatase*	-3.34
L3_67240	*Type I iodothyronine deiodinase*	7.16
L2_35211	*Type II antifreeze protein I*	3.28
L12_39497	*Type II iodothyronine deiodinase*	1.82
L12_89765	*Type III iodothyronine deiodinase*	2.88
L1_26330	*Type IV secretory pathway, VirD4 component*	2.04
L3_75827	*Type-1 angiotensin II receptor-associated protein-like*	-3.55
L2_32248	*Type-2 ice-structuring protein-like*	1.77
L12_34593	*Type-4 ice-structuring protein LS-12*	280.91
L2_44748	*Tyrosine 3-monooxygenase*	-3.18
L2_66960	*Tyrosine-protein kinase*	1.55

L2_42620	*Tyrosine-protein kinase Fyn*	-1.71
L1_69922	*Tyrosine-protein kinase fynb*	-2.27
L3_75926	*Tyrosine-protein kinase HCK*	2.86
L12_83842	*Tyrosine-protein kinase ITK/TSK*	-2.24
L12_76595	*Tyrosine-protein kinase JAK1*	-1.66
L2_13630	*Tyrosine-protein kinase JAK2-like*	-1.95
L2_62989	*Tyrosine-protein kinase JAK3*	-1.71
L12_87653	*Tyrosine-protein kinase Lck*	-1.87
L12_89607	*Tyrosine-protein kinase Lyn*	-2.47
L12_82194	*Tyrosine-protein kinase Mer*	-2.17
L12_79636	*Tyrosine-protein kinase SYK*	-2.24
L1_41538	*Tyrosine-protein kinase transforming protein erbB*	1.59
L12_85829	*Tyrosine-protein kinase transmembrane receptor ROR2*	-1.84
L12_84775	*Tyrosine-protein phosphatase non-receptor type 13*	-1.56
L1_46084	*Tyrosine-protein phosphatase non-receptor type 21*	-2.18
L1_5938	*Tyrosine-protein phosphatase non-receptor type 3*	1.68
L1_64145	*Tyrosine-protein phosphatase non-receptor type 3-like*	1.59
L2_41087	*Tyrosine-protein phosphatase non-receptor type 6*	2.73
L3_86635	*Tyrosine-protein phosphatase non-receptor type 9*	-1.92
L12_80282	*Tyrosyl-DNA phosphodiesterase 2*	-1.72
L1_21896	*U1 like C2H2 zinc finger, related*	1.84
L12_82499	*U3 small nucleolar RNA-interacting protein 2*	-1.60
L3_41039	*U6 snRNA-associated Sm-like protein LSm7*	-1.74
L1_50597	*U8 snoRNA-decapping enzyme-like*	1.65
L3_80839	*Ubiquilin-4*	3.80
L1_51946	*Ubiquinol-cytochrome c reductase, complex III subunit XI*	1.75
L12_85864	*Ubiquitin carboxyl-terminal hydrolase 3*	127.78
L2_52490	*Ubiquitin carboxyl-terminal hydrolase 38*	-1.54
L3_81496	*Ubiquitin carboxyl-terminal hydrolase 64E-like*	2.91
L2_29161	*Ubiquitin domain-containing protein 2-like*	-3.89
L3_77256	*Ubiquitin thioesterase OTUB2*	-1.80
L12_89778	*Ubiquitin-associated and SH3 domain-containing protein B*	-2.05
L2_46311	*Ubiquitin-associated domain-containing protein 2*	-1.82
L12_37773	*Ubiquitin-conjugating enzyme E2 N*	-1.65
L12_88906	*Ubiquitin-conjugating enzyme E2 W*	-2.41
L12_82114	*Ubiquitin-like modifier-activating enzyme ATG7*	-1.80
L3_39325	*Ubiquitin-like protein 5*	-1.52
L12_88914	*UDP-GlcNAc:betaGal beta-1,3-N-acetylglucosaminyltransferase 2*	4.80
L12_82595	*UDP-GlcNAc:betaGal beta-1,3-N-acetylglucosaminyltransferase 7*	-3.10
L12_87308	*UDP-glucose 6-dehydrogenase*	3.63
L2_68560	*UDP-glucose:glycoprotein glucosyltransferase 1-like*	-1.59
L12_71465	*UDP-glucuronosyltransferase*	4.43
L2_42524	*UDP-glucuronosyltransferase 1-5*	-2.41

L12_87074	*UDP-glucuronosyltransferase 1-7*	1.98
L12_70442	*UDP-glucuronosyltransferase 2B17*	2.07
L1_37136	*UDP-glucuronosyltransferase 2B33*	2.80
L12_80613	*UDP-glucuronosyltransferase 2C1*	2.66
L12_85370	*UDP-N-acetylglucosamine transporter*	-15.37
L12_87383	*UDP-N-acetylhexosamine pyrophosphorylase*	2.62
L12_60648	*UDP-N-acetylhexosamine pyrophosphorylase-like*	1.53
L1_61501	*UDP-N-acetylhexosamine pyrophosphorylase-like protein 1*	-3.32
L12_86521	*UMP-CMP kinase 2, mitochondrial*	2.53
L12_88817	*UNC93-like protein MFSD11*	-2.41
L2_49482	*Uncharacterized aarF domain-containing protein kinase 5*	1.99
L1_57979	*Uncharacterized family 31 glucosidase KIAA1161*	2.03
L1_22190	*Uncharacterized family 31 glucosidase KIAA1161-like*	2.03
L1_53691	*Uncharacterized membrane protein C1orf95*	-1.52
L12_48604	*Uncharacterized membrane protein C1orf95 homolog*	-1.90
L12_67576	*Uncharacterized methyltransferase YdaC*	1.65
L3_73349	*Uncharacterized methyltransferase ydaC-like*	1.61
L2_43028	*Uncharacterized monothiol glutaredoxin ycf64-like*	1.52
L3_73148	*Uncharacterized oxidoreductase C663.06c-like*	2.13
L1_81852	*Uncharacterized oxidoreductase C663.09c-like*	39.71
L12_84619	*Uncharacterized oxidoreductase YjhC*	-2.35
L2_20686	*Uncharacterized protein*	-1.78
L1_48541	*Uncharacterized protein*	-1.87
L2_47831	*Uncharacterized protein*	-1.99
L2_56055	*Uncharacterized protein*	-2.32
L12_76732	*Uncharacterized protein C12orf29 homolog*	-1.85
L12_76176	*Uncharacterized protein C12orf43*	-1.96
L1_8458	*Uncharacterized protein C14orf28 homolog*	1.60
L3_76941	*Uncharacterized protein C15orf26 homolog*	13.96
L3_74829	*Uncharacterized protein C15orf39 homolog*	-1.66
L12_82311	*Uncharacterized protein C17orf62 homolog*	-1.61
L12_47228	*Uncharacterized protein C1orf115-like*	2.19
L1_37045	*Uncharacterized protein C1orf194 homolog*	3.07
L1_50555	*Uncharacterized protein C2orf62 homolog*	-1.55
L12_80073	*Uncharacterized protein C3orf18*	-1.52
L12_87663	*Uncharacterized protein C3orf18 homolog*	-1.53
L2_63867	*Uncharacterized protein C3orf67 homolog*	-5.83
L12_79346	*Uncharacterized protein C4orf50*	1.55
L12_69844	*Uncharacterized protein C5orf4*	10.29
L12_87980	*Uncharacterized protein C5orf4 homolog*	12.41
L12_88267	*Uncharacterized protein C9orf174*	-3.70
L12_79821	*Uncharacterized protein F13E9.13, mitochondrial*	-1.52
L12_74137	*Uncharacterized protein KIAA0226-like*	-1.68
L12_76132	*Uncharacterized protein KIAA1522 homolog*	-1.58

L12_72821	*Uncharacterized protein KIAA1755*	-1.89
L1_46711	*Uncharacterized protein KIAA2013 homolog*	-1.53
L1_41991	*Uncharacterized protein LOC100002243*	692.44
L2_65445	*Uncharacterized protein LOC101061257*	-1.51
L12_89093	*Uncharacterized protein LOC101062984, partial*	4.75
L1_20645	*Uncharacterized protein LOC101064134, partial*	-2.56
L12_81717	*Uncharacterized protein LOC101066364*	-1.80
L1_31033	*Uncharacterized protein LOC101067273*	1.63
L12_89144	*Uncharacterized protein LOC101068298*	2.39
L1_57649	*Uncharacterized protein LOC101071133*	2.54
L2_52949	*Uncharacterized protein LOC101073348*	-1.74
L2_24087	*Uncharacterized protein LOC101078418*	-3.37
L12_41365	*Uncharacterized protein LOC101078886*	1.78
L12_80722	*Uncharacterized protein LOC101080246*	-1.79
L2_34087	*Uncharacterized protein LOC101156258*	-13.64
L1_4711	*Uncharacterized protein LOC101156905*	1.56
L1_42553	*Uncharacterized protein LOC101158559*	2.03
L2_69800	*Uncharacterized protein LOC101158564*	-1.60
L12_87718	*Uncharacterized protein LOC101159963*	-1.69
L12_79230	*Uncharacterized protein LOC101161596*	2.10
L1_48441	*Uncharacterized protein LOC101163127*	7.80
L12_73856	*Uncharacterized protein LOC101164665*	2.43
L12_86728	*Uncharacterized protein LOC101165404*	-1.95
L2_20384	*Uncharacterized protein LOC101165866*	-1.74
L12_73826	*Uncharacterized protein LOC101166209*	17.16
L1_8835	*Uncharacterized protein LOC101166792*	1.63
L12_84858	*Uncharacterized protein LOC101168137*	1.94
L12_31041	*Uncharacterized protein LOC101169195*	3.23
L2_61455	*Uncharacterized protein LOC101170212*	-2.17
L1_4897	*Uncharacterized protein LOC101170263*	1.97
L1_71110	*Uncharacterized protein LOC101171497*	2.25
L12_45147	*Uncharacterized protein LOC101172681*	-1.77
L12_75131	*Uncharacterized protein LOC101172949*	-6.15
L1_34378	*Uncharacterized protein LOC101174322*	-4.02
L2_2686	*Uncharacterized protein LOC101174919*	8.05
L1_23680	*Uncharacterized protein LOC101175024*	5.07
L12_74989	*Uncharacterized protein LOC101175200*	2.26
L12_75821	*Uncharacterized protein LOC101175339*	-5.51
L2_42107	*Uncharacterized protein YbiI*	2.22
L1_16983	*Uncharacterized signaling protein CC 0091*	1.68
L1_5843	*Unconventional myosin-Ib*	2.71
L12_85385	*Unconventional myosin-Ic*	-1.58
L1_45593	*Unconventional myosin-Ic-A*	2.15
L2_51298	*Unconventional myosin-IXb-like*	-1.69
L1_65695	*Unconventional myosin-Va*	-2.50

L2_89984	*Unconventional myosin-Vb*	-229.39
L12_88964	*Unconventional myosin-Vc*	-2.37
L12_89987	*Unconventional myosin-VIIa*	2.89
L12_88851	*Unconventional myosin-XV*	1.78
L4_60550	*Unique cartilage matrix-associated protein*	2.41
L3_59533	*UPF0184 protein C9orf16 homolog*	-2.47
L2_27242	*UPF0235 protein Atu2660*	-4.60
L12_83375	*UPF0444 transmembrane protein C12orf23 homolog*	-2.09
L3_49517	*UPF0449 protein C19orf25-like*	1.51
L12_81573	*UPF0462 protein C4orf33 homolog*	4.19
L12_85219	*UPF0554 protein C2orf43 homolog*	2.36
L1_9085	*UPF0565 protein C2orf69 homolog*	-1.84
L2_29173	*UPF0577 protein KIAA1324-like*	-2.42
L2_55288	*UPF0585 protein C16orf13 homolog A*	-1.57
L12_83694	*UPF0600 protein C5orf51 homolog*	-1.85
L12_80862	*UPF0692 protein C19orf54 homolog*	-1.55
L12_82888	*UPF0704 protein C6orf165 homolog*	-24.73
L12_38783	*UPF0708 protein C6orf162*	3.09
L2_58279	*UPF0764 protein C16orf89 homolog*	-1.80
L12_77806	*UPF0767 protein C1orf212 homolog*	1.51
L2_61962	*Up-regulator of cell proliferation-like*	-2.21
L1_62676	*Uracil nucleotide/cysteinyl leukotriene receptor*	-2.73
L12_85081	*Uridine phosphorylase 1*	-1.60
L1_53247	*Uridine phosphorylase 2*	3.14
L12_81434	*Uromodulin*	107.97
L12_81240	*USP6 N-terminal-like protein*	-1.61
L1_32465	*UvrABC system protein A*	2.03
L2_37164	*UvrABC system protein B*	1.94
L2_67539	*Vacuolar fusion protein MON1 homolog A*	1.70
L12_89642	*Vacuolar protein sorting-associated protein 11 homolog*	-1.69
L1_18367	*Vacuolar protein sorting-associated protein 13A-like*	1.65
L12_86490	*Vacuolar protein sorting-associated protein 16 homolog*	-2.02
L12_60076	*Vacuolar protein sorting-associated protein 18 homolog*	-1.73
L3_70293	*Vacuolar protein sorting-associated protein 29*	-1.65
L12_88444	*Vacuolar protein sorting-associated protein 33A*	-1.60
L12_90074	*Vacuolar protein sorting-associated protein 33B*	-1.92
L12_73846	*Vacuolar protein sorting-associated protein 37B*	1.72
L12_80628	*Vacuolar protein sorting-associated protein 41 homolog*	-2.31
L12_87368	*Vacuolar protein sorting-associated protein 4B*	-2.58
L12_87160	*Vacuole membrane protein 1*	2.86
L12_79217	*Vascular cell adhesion protein 1*	-3.01
L12_89121	*Vascular endothelial growth factor A-A*	1.70
L2_49040	*Vascular endothelial growth factor D-like*	-2.99
L3_47850	*Vascular endothelial growth factor receptor 1*	1.81
L2_20918	*Vascular endothelial growth factor receptor 3*	1.50

L2_27495	*Vasoactive intestinal polypeptide receptor 1*	1.71
L12_84806	*Vasopressin V2 receptor*	-4.45
L2_65738	*Vasorin*	2.08
L2_38066	*Vasoticin receptor type V1a2*	-2.11
L2_53398	*Versican core protein*	-1.58
L3_37326	*Very-long-chain (3R)-3-hydroxyacyl- dehydratase 1*	-2.63
L12_80711	*Very-long-chain (3R)-3-hydroxyacyl- dehydratase 2*	3.66
L12_81846	*Very-long-chain enoyl-CoA reductase*	1.84
L12_78127	*Vesicle transport protein SFT2A*	-1.97
L12_76948	*Vesicle transport protein SFT2B*	1.53
L3_55127	*Vesicle-associated membrane protein 7*	-1.69
L3_74513	*Vesicle-associated membrane protein 8*	-1.78
L12_59157	*Vesicle-associated membrane protein-associated protein A*	2.15
L3_50441	*Vesicle-trafficking protein SEC22b-B*	1.58
L3_83219	*Vigilin*	1.71
L3_77279	*Villin-1*	1.76
L3_68715	*Vimentin*	-1.90
L1_63638	*Vimentin A2*	-1.92
L12_87309	*VIP peptides*	-2.87
L12_82254	*VIP36-like protein*	-1.86
L12_84273	*Viral dihydrofolate reductase*	15.27
L12_74721	*Vitamin D3 receptor A*	1.67
L1_65696	*Vitamin D3 receptor B*	2.99
L12_60921	*Vitelline membrane outer layer protein 1 homolog*	168.35
L4_90209	*Vitellogenin-1*	2.82
L3_78673	*Vitronectin*	1.88
L12_85869	*Voltage-dependent anion-selective channel protein 1*	-1.61
L12_85226	*Voltage-dependent anion-selective channel protein 2*	1.50
L12_88782	*Voltage-dependent calcium channel gamma-like subunit*	12.81
L2_69332	*Voltage-dependent calcium channel subunit alpha-2/delta-3*	1.92
L12_76446	*Voltage-gated hydrogen channel 1*	-12.36
L12_66146	*Von Willebrand factor A domain-containing protein 1*	-2.27
L3_74697	*Von Willebrand factor A domain-containing protein 1-like*	-1.67
L12_89452	*Von Willebrand factor A domain-containing protein 7*	35.62
L1_70152	*Von Willebrand factor C domain-containing protein 2-like*	-1.89
L12_86575	*V-set and immunoglobulin domain-containing protein 10*	1.50
L2_47522	*V-set and transmembrane domain-containing protein 4*	-1.53
L12_84431	*V-set and transmembrane domain-containing protein 5*	-1.64
L12_89400	*V-type proton ATPase 116 kDa subunit a isoform 1*	-1.99
L12_89350	*V-type proton ATPase 116 kDa subunit a isoform 3*	-3.46
L2_28893	*V-type proton ATPase 16 kDa proteolipid subunit*	-3.23
L12_89549	*V-type proton ATPase 21 kDa proteolipid subunit*	-2.44
L12_89125	*V-type proton ATPase catalytic subunit A*	-4.27

L12_88873	*V-type proton ATPase subunit B, brain isoform*	-2.87
L12_83276	*V-type proton ATPase subunit C 1-A*	-2.71
L12_85122	*V-type proton ATPase subunit D*	-1.74
L12_87601	*V-type proton ATPase subunit d 1*	-2.41
L12_85412	*V-type proton ATPase subunit H*	-2.20
L12_87712	*V-type proton ATPase subunit S1*	-2.91
L2_71088	*VWFA and cache domain-containing protein 1*	2.69
L12_88137	*WAP four-disulfide core domain protein 1*	-2.44
L12_81513	*WAP four-disulfide core domain protein 3*	-1.83
L2_48202	*WASH complex subunit 7*	-2.38
L12_86880	*WASH complex subunit CCDC53*	-2.81
L12_72582	*WASH complex subunit FAM21*	-1.86
L12_85193	*WASH complex subunit FAM21B*	-1.61
L1_18837	*WASH complex subunit FAM21-like*	-2.70
L12_82634	*WASH complex subunit strumpellin*	-1.61
L1_54721	*WD repeat- and FYVE domain-containing protein 4*	-2.25
L12_75946	*WD repeat and SOCS box-containing protein 1*	1.60
L3_9622	*WD repeat and SOCS box-containing protein 2-like*	-1.91
L1_28652	*WD repeat domain phosphoinositide-interacting protein 3*	-3.22
L12_82168	*WD repeat, SAM and U-box domain-containing protein 1*	2.81
L12_79913	*WD repeat-containing protein 11*	-1.51
L12_54225	*WD repeat-containing protein 17*	-20.52
L2_74120	*WD repeat-containing protein 25*	-2.54
L12_84427	*WD repeat-containing protein 41*	-1.93
L2_26431	*WD repeat-containing protein 7*	-1.68
L1_49337	*WD repeat-containing protein 78*	11.27
L12_85196	*WD repeat-containing protein 81*	59.20
L1_49803	*WD repeat-containing protein 96*	-1.88
L2_8267	*WD repeat-containing protein mio*	1.63
L12_83325	*WD repeat-containing protein WRAP73*	-2.18
L2_38132	*Wee1-like protein kinase*	-1.67
L2_41738	*Wee1-like protein kinase 1-B*	-1.90
L1_47588	*Wee1-like protein kinase 2*	1.62
L2_36611	*Williams-Beuren syndrome chromosomal region 27 protein-like*	-2.92
L2_50153	*Wiskott-Aldrich syndrome protein*	-1.59
L12_72667	*WW domain binding protein 1-like*	1.51
L12_88499	*WW domain-binding protein 2*	2.04
L12_74108	*WWE*	2.47
L12_88261	*Xaa-Pro aminopeptidase 2*	2.67
L12_85681	*Xanthine dehydrogenase/oxidase*	-4.04
L12_90275	*Xin actin-binding repeat-containing protein 1*	-9.44
L1_18286	*Xin actin-binding repeat-containing protein 1-like*	-4.68
L12_87647	*Xylulose kinase*	1.93
L12_67886	*Yae1 domain-containing protein 1*	1.59

L12_83450	*YrdC domain-containing protein, mitochondrial*	-1.55
L2_6083	*Zf-C3HC4 2*	-1.58
L2_55661	*Zgc:174877 protein*	1.61
L2_25934	*Zinc finger and BTB domain-containing protein 2*	-1.60
L2_47646	*Zinc finger and BTB domain-containing protein 8A*	1.56
L12_89037	*Zinc finger C2HC domain-containing protein 1A*	-1.61
L12_75520	*Zinc finger CCCH domain-containing protein 7B*	1.79
L2_36286	*Zinc finger E-box-binding homeobox 1*	-2.67
L1_56383	*Zinc finger FYVE domain-containing protein 26*	-2.19
L1_39511	*Zinc finger protein 135*	1.53
L2_64828	*Zinc finger protein 185*	2.68
L1_56207	*Zinc finger protein 217*	-1.82
L1_26395	*Zinc finger protein 292-like*	-1.60
L2_43723	*Zinc finger protein 295*	-1.66
L12_73593	*Zinc finger protein 331-like*	-1.55
L3_77204	*Zinc finger protein 516*	-1.82
L1_8316	*Zinc finger protein 516-like*	-1.53
L2_53640	*Zinc finger protein 536*	-1.54
L1_11715	*Zinc finger protein 536-like*	-1.94
L12_76697	*Zinc finger protein 541*	-2.02
L12_63440	*Zinc finger protein 595*	-1.61
L12_30387	*Zinc finger protein 64*	1.64
L2_36926	*Zinc finger protein 7*	1.96
L1_56908	*Zinc finger protein 800*	-1.59
L1_24852	*Zinc finger protein 821*	2.23
L2_70531	*Zinc finger protein 878-like*	-1.52
L1_42132	*Zinc finger protein basonuclin-2*	3.54
L12_71483	*Zinc finger protein castor homolog 1*	1.90
L2_66342	*Zinc finger protein Gfi-1*	-3.15
L12_86942	*Zinc finger protein Helios*	-1.66
L12_81341	*Zinc finger protein ZFAT*	1.96
L2_41725	*Zinc finger protein ZFPM1*	2.33
L12_88661	*Zinc finger protein-like 1*	1.64
L2_52709	*Zinc finger transcription factor Trps1*	-2.10
L1_48076	*Zinc finger with UFM1-specific peptidase domain protein*	-1.63
L1_21643	*Zinc fingers and homeoboxes protein 1-like*	2.11
L1_54718	*Zinc fingers and homeoboxes protein 3*	-1.63
L1_71077	*Zinc fingers and homeoboxes protein 3-like*	-1.76
L3_32588	*Zinc phosphodiesterase ELAC protein 2*	1.73
L2_42691	*Zinc transporter 2*	-3.44
L12_88849	*Zinc transporter 4*	-2.95
L3_36953	*Zinc transporter 7*	1.57
L12_83994	*Zinc transporter 8*	-51.39
L12_84926	*Zinc transporter ZIP1*	-2.53
L2_43374	*Zinc transporter ZIP10*	-1.64

L12_80618	*Zinc transporter ZIP13*	1.53
L1_42684	*Zinc transporter ZIP4*	4.67
L12_68504	*Zinc transporter ZIP4-like*	4.47
L12_85588	*Zinc transporter ZIP8*	41.93
L1_22513	*Zona pellucida sperm-binding protein*	2.91
L1_14436	*Zona pellucida sperm-binding protein 3-like*	55.10
L12_88408	*Zonadhesin*	11.74
L12_54131	*Zonadhesin-like*	14.82
L12_46270	*Zonadhesin-like*	13.23
L12_76912	*Zyxin*	-1.63
